# Years of Life Lost (YLL) Due to Short-Term Exposure to Ambient Air Pollution in China: A Systematic Review and Meta-Analysis

**DOI:** 10.3390/ijerph182111467

**Published:** 2021-10-31

**Authors:** Yang Ni, Wang Song, Yu Bai, Tao Liu, Guoxing Li, Ying Bian, Qiang Zeng

**Affiliations:** 1 Tianjin Centers for Disease Control and Prevention, Tianjin 300011, China; ni1989yang@163.com (Y.N.); wang.song@connect.um.edu.mo (W.S.); 2 State Key Laboratory of Quality Research in Chinese Medicine, Institute of Chinese Medical Sciences, University of Macau, Macao 999078, China; bianyingumac@126.com; 3 School of Public Health, Tianjin Medical University, Tianjin 300070, China; berrypei1996@126.com (Y.B.); gwyxstt@163.com (T.L.); 4 Department of Occupational and Environmental Health, School of Public Health, Peking University, Beijing 100191, China; liguoxing@bjmu.edu.cn

**Keywords:** ambient air pollution, years of life lost, meta-analysis, short-term exposure, disease burden, health effects

## Abstract

(1) Background: Years of life lost (YLL) as a surrogate of health is important for supporting ambient air pollution related policy decisions. However, there has been little comprehensive evaluation of the short-term impact of air pollution on cause-specific YLL, especially in China. Hence in this study, we selected China as sentinel region in order to conduct a meta-analysis to evaluate disease-specific YLL due to all the main ambient air pollutants. (2) Methods: A meta-analysis was conducted to evaluate disease-specific YLL due to the main ambient air pollutants in China, and 19 studies were included. We conducted methodological quality and risk of bias assessment for each included study as well as for heterogeneity and publication bias. Subgroup analysis and sensitivity analysis were also performed. (3) Results: Meta-analysis indicated that increases in PM_2.5_, PM_10_, SO_2_ and NO_2_ were associated with 1.99–5.84 years increase in YLL from non-accidental diseases. The increase in YLL to cardiovascular disease (CVD) was associated with PM_10_ and NO_2_, and the increase in YLL to respiratory diseases (RD) was associated with PM_10_. (4) Conclusions: Ambient air pollution was observed to be associated with several cause-specific YLL, increasing especially for elderly people and females.

## 1. Introduction

As the largest developing country in the world, China has experienced a rapid economic growth in recent decades. However, along with this, China is facing a problem of serious ambient air pollution caused by energy consumption from coal combustion, as well as increasing industrial waste and motor vehicle use, which make it a country with one of the most serious levels of air pollution in the world [1,2]. Therefore, China is at the most important stage of ambient air pollution control and more and more synthetically epidemiologic evidence is needed to support ambient air pollution related policy-making [3]. Previous comprehensive epidemiologic studies in China have linked ambient air pollution with increased mortality from all of kinds of disease [4,5,6]. However, the approach, using mortality as a surrogate of human health, gives equal weight to each death and may fail to consider the effects of the death age [7], so is less sensitive in assessing the premature death, which is a major factor to be considered in policy-making and resource allocation [8]. In contrast, years of life lost (YLL) as an indicator of disease burden can take premature death and life expectancy into consideration [7,9]. Therefore, studies using YLL as a surrogate for health might be important for supporting ambient air pollution related policy decisions in a timely manner.

Several previous environmental studies in countries worldwide have used YLL as a health indicator to investigate the association of ambient air pollution with health outcomes [10,11,12,13,14,15,16,17,18,19]. However, most studies, such as Global burden of disease (GBD) research, have been on the effects of long-term exposure to air pollution on YLL [13,14,15,16,17,18,19]. GBD collaborators reported the association between YLL and ambient air pollution in China in 2010 [17,18,19]. However, GBD studies have focused more on the long-term trends or annual trends of air pollution exposure, thereby the comprehensive associations between short-term exposure to air pollution and YLL in China are still scarce. To deal with this concern, in 2013, a Chinese researcher used the time-series study design to retrospectively investigate the effects of short-term exposure to ambient air pollution on YLL [7]. Since then, several studies have been conducted to clarify the associations between short-term exposure to ambient air pollution and YLL in China [20,21,22,23,24,25]. However, results were inconsistent and contradictory. For instance, some studies detected specific associations between positive particulate matter smaller than 10 μm in aerodynamic diameter (PM_10_) and cardiovascular disease (CVD) [26,27]. However, others found no association [21,28,29]. To our knowledge, there has been little comprehensive evaluation of the short-term impact of air pollution on cause-specific YLL in China.

Hence, this study selected China as sentinel region to conduct this systematic review and meta-analysis, in order to evaluate and compare disease-specific YLL due to short-term exposure to some of the main ambient air pollutants, including particulate matter smaller than 2.5 μm in aerodynamic diameter (PM_2.5_), PM_10_, sulfur dioxide (SO_2_), nitrogen dioxide (NO_2_), carbon monoxide (CO) and ozone (O_3_).

## 2. Materials and Methods

### 2.1. Search Strategy

This systematic review and meta-analysis complied with the Preferred Reporting Items for Systematic Reviews and Meta-Analyses (PRISMA) guidelines [30]. We formulated the specific research question according to the PECO statement (Population, Exposure, Comparator and Outcomes) [31]. The definition of the research question of this systematic review and meta-analysis is as follows: “Is short-term exposure to ambient air pollution associated with changes in YLL?” All papers were identified in four English databases (PubMed, EMBASE, Web of science and MEDLINE) and two Chinese databases (China National Knowledge Infrastructure and Wanfang Data) by using the combinations of ambient air pollution terms, YLL terms and location terms up to 10 April 2020. The full details of the search strategies are presented in the Supplementary Material Part A. The literature search was limited to the Chinese and English languages. We also searched references of potential interest using “snowballing” methods including backward and forward citations search, for the purpose of screening the reference lists of eligible articles and checking articles that have cited other eligible articles [32].

### 2.2. Selection Criteria

**Figure 1:**
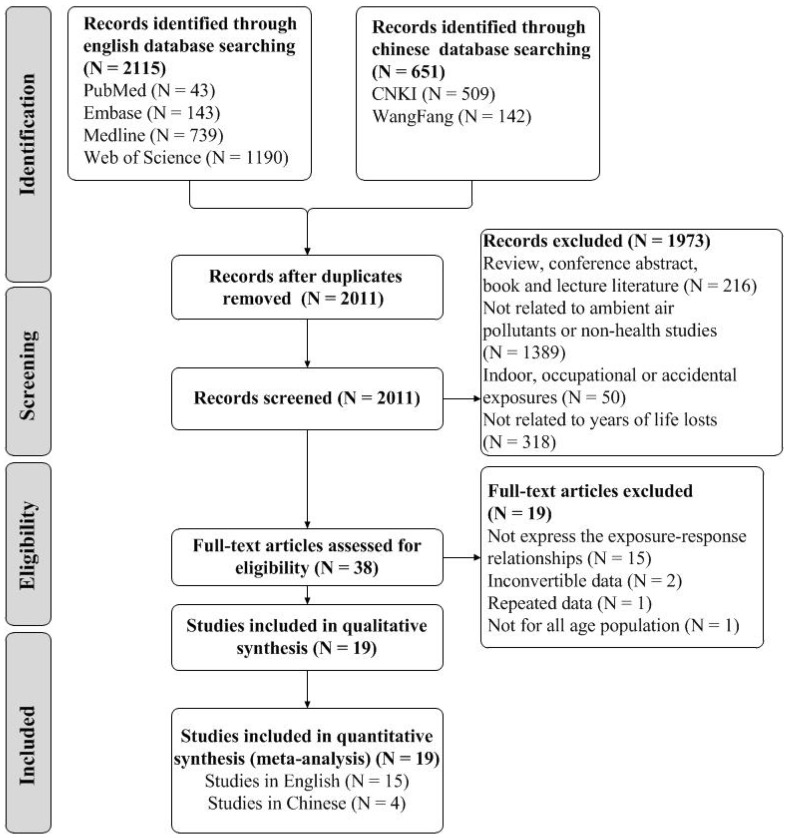
Flowchart of literature for search and identification.

The PRISMA flow diagram is shown in Figure 1. After removing the duplicates, all the titles, abstracts and full texts were screened by two authors to assess for eligibility (Y.N. and Yu Bai), independently. When there was disagreement, the adjudication of the third author (Q.Z.) was used.

The inclusion criteria were as follows: (1) epidemiologic studies on the health impact of short-term exposure to PM_2.5_, PM_10_, SO_2_, NO_2_, O_3_ or CO; (2) the health outcomes were related to YLL; (3) original studies expressed quantitative exposure–response relationships between mentioned pollutants and YLL; (4) the estimated relationships were expressed in terms of changes in YLL with 95% confidence intervals (CI) associated with unit change in pollutant mass concentration; (5) exposure to ambient air pollution, not including indoor, occupational or accidental exposure.

The exclusion criteria were as follows: (1) reviews, systematic reviews, meta-analyses, lecture literature, conference reorts, book and conference abstracts; (2) not related to air pollutants or non-health studies; (3) exposure to indoor, occupational or accidental pollution; (4) not related to YLL; (5) not expressing the exposure–response relationship; (6) inconvertible data or repeated data; (7) not for the whole age population.

### 2.3. Data Collection

For each study included, the following information was collected: author′s name, publication year, city, study period, design and size, health outcome classification standards (International Classification of Disease, ICD, code), YLL calculation method, studied pollutants and concentrations, exposure assessment methods, lag pattern used, stratified data setting, adjusted confounders, effect estimates and 95% CI. In the case of the multi-district studies, if the overall effect estimates were not reported, each district-specific effect estimate was extracted as an independent data set [33]. For lag patterns, a lag selection proposed by previous studies was adopted [34,35]: (1) if only one lag estimate was reported, it was directly used; (2) if multiple lags were reported, we chose, in order of priority, the lag that the studies focused on or stated, the lag that was statistically significant, or the lag that was the largest effect estimate. Furthermore, in this study, we focused mainly on the single-pollutant model. Two authors (Y.N. and T.L.) independently extracted the information of each study, and in any conflict, the adjudication of the third author (Q.Z.) was used.

### 2.4. Methodological Quality Assessment

To the best of our knowledge, there has been no established quality assessment tool to assess the quality of time-series studies [36]. Therefore, we adapted the quality assessment tool, including three items, which was applied in several previous studies [4,37,38,39]. In this study, 0–3 points were assigned for the following three items: validation of the health outcome classified (if ICD code was used as the health outcome classified standards, 1 point was scored, otherwise 0 point was adapted), the quality of air pollution exposure measurements (if the measurements were performed as an average daily value from 24-hourly monitoring with less than 25% missing data, 1 point was scored, or else 0 points) and the extent of confounder adjustments (if all the confounders including long-term trends, seasonality and temperature were controlled, 1 point was scored; if not, 0 point was scored; 1 point was added with each additional confounder adjustment until the total point for this item was 3, and the additional confounder included the day of week, relative humidity, air pressure and public holidays) [9]. A score of five was considered as a high quality study; otherwise it is considered a low quality study.

### 2.5. Risk of Bias Assessment

The risk of bias for each study was further assessed according to the Office of Health Assessment and Translation (OHAT) tool by the National Institutes of Environmental Health Sciences-National Toxicology Program (NIEHS-NTP), and the Navigation Guide by the University of California, San Francisco [40,41], as there were no established tools for time-series studies. Seven items, i.e., selecting bias, confounding, exposure assessment, outcome assessment, incomplete outcome data, selective reporting and conflict of interest were assessed for each study according to specific criteria used by previous studies [42]. The full details of the assessment criteria are shown in Table S1. Five levels (“low”, “probably low”, “probably high”, “high” and “not applicable”) were classified for each item. Two authors (Y.N. and G.L.) independently assessed the risk of bias for each study. According to the OHAT guidelines, if any studies were evaluated as “high” or “probably high” risk for most of the key items (exposure assessment, outcome assessment and confounding) or other items, we excluded these studies by following the research methodology [42].

### 2.6. Statistical Analysis

Our analysis focused on the associations of short-term exposure to the main ambient air pollutants (including PM_2.5_, PM_10_, SO_2_, NO_2_, and O_3_) with the cause-specific YLL for the entire population (all ages). Firstly, we standardized the effect estimates with 95% CI, extracted from the studies with a 10 μg/m^3^ change as the increasing unit (the detailed information for the standardized method was shown in the Supplementary Material Part B). For O_3_, we converted the estimates expressed in parts per billion (ppb) to 10 μg/m^3^ using 1.96 μg/m^3^ per 1 ppb for the standard temperature and pressure [43,44]. Secondly, a heterogeneity test, by providing the *p*-values of the Cochran Q test, and I^2^ were conducted to assess the between-study heterogeneity [45]. If *p* < 0.05 or I^2^ ≥ 50%, it indicated that there was a larger heterogeneity between the studies, so the random-effects model was used to calculate the pooled effect estimates [46,47]; otherwise, a fixed-effects model was used [48]. The full details of the selection strategies are presented in the Supplementary Material Part C. Publication bias was assessed using Doi plot for results in which the number of studies was more than 5 and the Luis Furuya-Kanamori (LFK) index as in previous studies [4,49]. LFK index < indicates “no asymmetry”, LFK index between indicates “minor asymmetry”, LFK index > indicates “major asymmetry” (according to MetaXL User Guide, www.epigear.com, accessed on 10 April 2020) [50]. The trim and fill method was also conducted. Then, we conducted sensitivity analysis to examine the stability of the pooled estimates by excluding one study one time. Furthermore, to compare the differences of the associations between subgroups, stratified analysis was conducted by age and gender, and the effect estimates with 95% CI for each subgroup were calculated, respectively. For the subgroup analysis, we only focused on the associations of the ambient air pollution with the YLL of non-accidental diseases, CVD and respiratory diseases (RD). As there was heterogeneity in the cut-off age for different studies included, we focused on the elderly people and younger people in the subgroup analysis of age, rather than a specific age group such as ≥75 years group. All statistical analyses were conducted using STATA software (version 14.0) for all tests. *p* < 0.05 (2-tailed) was considered statistically significant.

## 3. Results

### 3.1. Literature Retrieval

The flowchart for the literature search and identification is shown in Figure 1. A total of 2766 peer-reviewed records were identified from our search, with 2115 records via English database searching and 651 records via Chinese database searching, respectively. After duplicates were removed, 2011 records were left for screening for titles and abstracts, along with 1973 records excluded. Then, we reviewed the full-text of the remaining 38 records, and 19 full-text articles were excluded for not expressing exposure–response relationships (N = 15), inconvertible data (N = 2), repeated data (N = 1) or not for the whole age population (N = 1). Finally, 19 studies (15 studies in English [7,21,22,23,24,25,26,27,28,29,51,52,53,54,55] and four studies in Chinese [56,57,58,59]) met the stated inclusion criteria and were included in the meta-analysis. Among the included studies, there were two conducted by the same group at different times with the same pollutants and population [26,54]. However, as the two studies reported different results, one expressing the overall effect estimates [26] and the other expressing the subgroup effect estimates by age and gender [54], these two studies were both selected.

### 3.2. Characteristics and Quality of the Included Studies

The baseline characteristics of the 19 included studies are illustrated in Figure S1 with more details in Table S2. Of the 19 included studies, most investigated the effects of ambient air pollution on YLL due to non-accidental disease (N = 11), followed by CVD (N = 8), RD (N = 6), ischemic heart disease (IHD, N = 4), chronic obstructive pulmonary disease (COPD) and stroke (N = 3), acute myocardial infarction (AMI) and diabetes mellitus (N = 1). Eighteen studies used the ICD10 code as the outcome assessment standard, and another study did not mention the standard [7]. For exposure assessment, most studies considered PM_10_ (N = 13), followed by SO_2_ (N = 8), NO_2_ (N = 8), PM_2.5_ (N = 7) and O_3_ (N = 2). The exposure measurements for all included studies were performed daily with less than 25% missing data. The major confounders including long-term trends, seasonality, temperature, relative humidity and day of week were adjusted for most studies, except for one study without adjusting seasonality and one study without adjusting seasonality and temperature [51,56]. Six studies adjusted air pressure [7,24,27,28,53,56] and four studies controlled for public holiday [23,24,53,56].

According to the quality assessment tool, sixteen studies were regarded as ‘high quality’, whereas three studies were regarded as ‘low quality’ for the reasons that one study did not use the ICD 10 code for outcome assessment [7], and two studies did not control the confounders of seasonality and temperature [51,56].

### 3.3. Risk of Bias Assessment

The risk of bias for the individual studies is presented in Figure S2; most of the studies were regarded as ‘low risk’ for most items, apart exposure assessment which was usually assessed as ‘probably low risk’ and outcome assessment, which was ‘probably high risk’ in one study. The more analytical details of each study’s risk of bias assessment are provided in Supplementary Material Part B. None of the included studies were regarded as ‘high risk’ or ‘probably high risk’ for all three key criteria (Figure S2); hence, we included all the studies for analysis.

### 3.4. Associations between Ambient Air Pollution and Cause-Specific YLL

**Figure 2:**
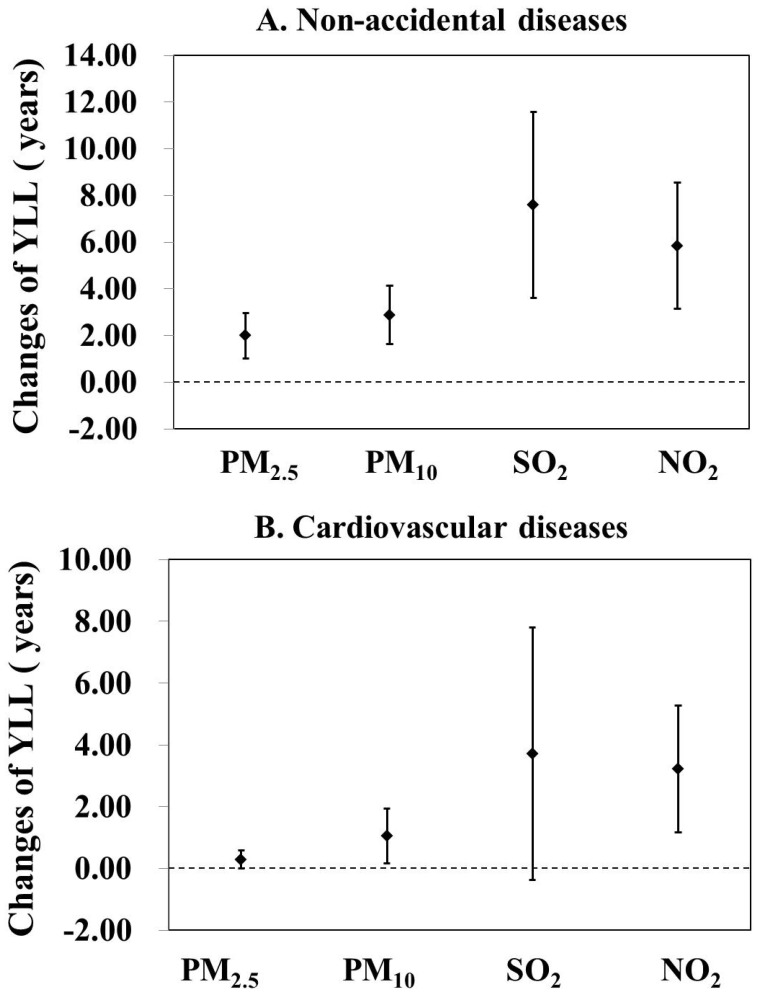

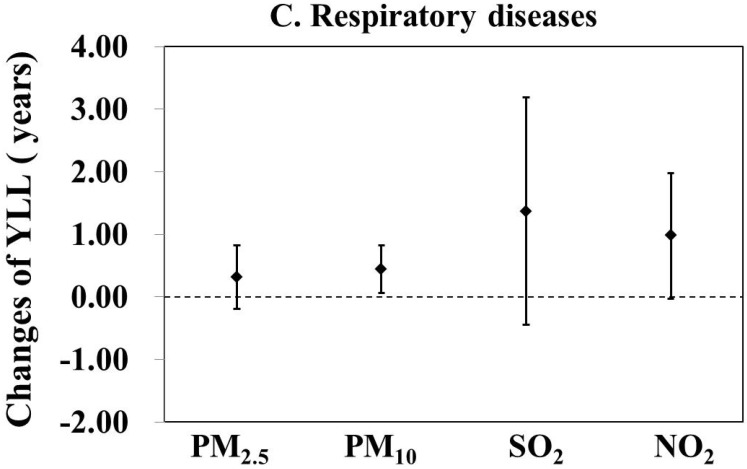
Meta-analysis results for the association between major ambient air pollutants and cause-specific YLL in China. Results are expressed in changes in disease-specific YLL per 10 μg/m^3^ increase in PM_2.5_, PM_10_, SO_2_ and NO_2_ with 95% confidence intervals. PM_2.5_: particle with aerodynamic diameter ≤ 2.5 μm; PM_10_: particle with aerodynamic diameter ≤ 2.5 μm; SO_2_: sulfur dioxide; NO_2_: nitrogen dioxide. (**A**) Non-accidental diseases, NPM_2.5_ = 3, NPM_10_ = 8, NSO_2_ = 6, NNO_2_ = 6. (**B**). cardiovascular diseases, NPM_2.5_ = 3, NPM_10_ = 5, NSO_2_ = 3, NNO_2_ = 2. (**C**). respiratory diseases, NPM_2.5_ = 2, NPM_10_ = 5, NSO_2_ = 3, NNO_2_ = 2.

The associations between ambient air pollution and YLL are shown in Figure 2. As only two studies were included in our study for O_3_, one of these investigating the effects of O_3_ on the YLL of COPD [22], the other focusing on the effects of O_3_ on the YLL of AMI [23], we did not pool the effect estimates of O_3_ in Figure 2. As shown in Figure 2, we found that 10 μg/m^3^ increases in PM_2.5_ (N = 3, *p* = 0.519, τ^2^ = 0.000, I^2^ = 0.0%), PM_10_ (N = 8, *p* < 0.001, τ^2^ = 3.046, I^2^ = 79.9%), SO_2_ (N = 6, *p* < 0.001, τ^2^ = 17.754, I^2^ = 88.9%) and NO_2_ (N = 6, *p* < 0.001, τ^2^ = 6.885, I^2^ = 86.7%) were associated with 1.99 (95% CI: 1.00, 2.97) years, 3.33 (95% CI: 1.85, 4.81) years, 7.59 (95% CI: 3.61, 11.57) years and 5.84 (95% CI: 3.15, 8.54) years increasing for YLL of non-accidental diseases, respectively. For CVD, we found that 10 μg/m^3^ increases in PM_10_ (N = 5, *p* = 0.005, τ^2^ = 0.642, I^2^ = 73.1%) and NO_2_ (N = 2, *p* = 0.781, τ^2^ = 0.000, I^2^ = 0.0%) were significantly associated with 1.05 (95% CI: 0.17, 1.94) years and 3.22 (95% CI: 1.16, 5.27) years increasing for YLL of CVD, respectively. No apparent associations between PM_2.5_ (N = 3, *p* = 0.845, τ^2^ = 0.000, I^2^ = 0.0%), SO_2_ (N = 3, *p* < 0.001, τ^2^ = 10.974, I^2^ = 87.4%) and YLL of CVD were found. For RD, we found 0.44 (95% CI: 0.06, 0.82) years increasing for YLL of RD with per 10 μg/m^3^ increases in PM_10_ (N = 5, *p* = 0.019, τ^2^ = 0.101, I^2^ = 66.2%). However, no significant effects were observed in PM_2.5_ (N = 2, *p* = 0.923, τ^2^ = 0.000, I^2^ = 0.0%), SO_2_ (N = 3, *p* = 0.005, τ^2^ = 1.9823, I^2^ = 81.2%) and NO_2_ (N = 2, *p* = 0.871, τ^2^ = 0.000, I^2^ = 0.0%). Table 1 shows the associations between ambient air pollutants and YLL of other diseases (including COPD, IHD, stroke, AMI and diabetes mellitus). The pooled effect estimates of PM_10_ on COPD (N = 2, *p* = 0.079, τ^2^ = 0.088, I^2^ = 67.7%), IHD (N = 3, *p* = 0.001, τ^2^ = 0.349, I^2^ = 86.2%) and stroke (N = 3, *p* = 0.285, τ^2^ = 0.018, I^2^ = 20.2%) and the pooled effect estimates of PM_2.5_ on COPD (N = 2, *p* = 0.124, τ^2^ = 0.401, I^2^ = 57.6%) were statistically significant. No significant pooled effect estimates were found for other diseases. The forest plots are shown in Figures S3–S6. Due to the limited number of the studies, we did not generate a pooled effect estimate for air pollution exposure on AMI and diabetes mellitus, SO_2_ or NO_2_ exposure and stroke, PM_2.5_ exposure and IHD, or O_3_ exposure and COPD.

### 3.5. Subgroup Analysis by Gender and Age

The associations of YLL for non-accidental diseases, CVD and RD with ambient air pollution stratified by gender and age are shown in Table 2. We found that the effects of most of the ambient air pollution on YLL for non-accidental diseases, CVD and RD in elderly people were stronger than those for younger people. The effects of PM_2.5_ and PM_10_ with the YLL of CVD in younger people were stronger than those in elderly people, but statistically significant associations were found only in the elderly. For different genders, the effects of most of the ambient air pollution on YLL for non-accidental diseases, CVD and RD in females were higher than those for males, except for the associations of PM_10_ with the YLL of non-accidental diseases and NO_2_ with the YLL of CVD, in which contrary results were found. The forest plots are shown in Figures S7 and S8.

### 3.6. Publication Bias and Sensitivity Analysis

The publication bias was assessed using a Doi plot for results in which the number of studies were more than 5, and the LFK index in Figure S9. For the YLL of non-accidental diseases, Doi plot and LFK index indicated major asymmetry for PM_10_, SO_2_ and NO_2_ (Figure S9A–C), the LFK index were 7.10, 6.15 and 7.50, respectively. Similar major asymmetry (LFK index = 5.05) was showed for PM_10_ on the YLL of RD (Figure S9D). However, for YLL of CVD, minor asymmetry (LFK index = −1.40) was found for PM_10_ (Figure S9F). The results before and after adjusting by the trim and filled method are shown in Table S3. No significant changes were found for most of the effects estimates for the case study of China, except the effects estimates of NO_2_ on the YLL of non-accidental diseases and PM_10_ on the YLL of RD, which changed from significant to non-significant. Sensitivity analyses, excluding one single study, did not materially change the pooled effect estimates (Figure S10). Due to limited data, we did not perform the Doi plot with LFK index and sensitivity analyses for the pooled effect estimates for which the numbers of studies were less than 5.

## 4. Discussion

In this meta-analysis, we synthesized 19 studies to comprehensively assess the associations of some of the main ambient air pollutants with multiple cause-specific YLL. We observed that short-term exposure to ambient air pollution was associated with increasing risk of YLL for several diseases, especially for non-accidental diseases, CVD, RD, COPD and stroke. The effects were stronger in elderly people and females than those of younger people and males. As the number of included studies was small, confidence in the pooled estimates needs to be further confirmed by future meta-analysis to draw more definitive conclusions.

In our study, the highest short-term impact on YLL has was found to be higher for the gaseous pollutants (SO_2_ and NO_2_) than that for PM, which is consistent with the meta-analysis of mortality in China [60,61]. Nowadays, public concern and awareness about ambient air pollution and health have risen to an unprecedented level in China [62]. However, compared with PM, less attention has been paid to gaseous pollutants and their control. Although there is a possibility that the observed effects of gaseous pollutants on YLL might be due to PM or other unmeasured pollutants such as ultrafine-particle [63], these results also suggest that gaseous pollutants might also play an important role in the YLL effects of the air pollution mixture. From the perspective of pollution control, our findings indicated that the control efforts and policies regarding gaseous pollution require further attention and should be strengthened. Of note, due to the limitation of the existing research, our study only focused on some of the main ambient air pollutants. Other ambient air pollution agents such as ultrafine-particles [64], CO [65] and the constituents of PM [66], which may also have significant effects on YLL, were not included in this study due to the lack of relative research. Therefore, further study is warranted and more efforts should be paid to fill this research gap.

Our results on the cause specific risk found that short-term exposure to ambient air pollution has the greatest effect on non-accidental disease-specific YLL. For the cardiorespiratory diseases specific risk, the highest pooled effect estimates were found between PM_10_ and CVD. Meanwhile, no changes of this association were found by applying the trim and fill method and sensitivity analyses (Table S3 and Figure S10E). The consistency of the association between PM_10_ and CVD in these analyses is convincing. These results were confirmed by studies in Colombia, which estimated YLL [10]. However, these results were inconsistent with the meta-analysis of mortality in China [67]. This heterogeneity may be attributed to the fact that YLL accounts for the age at which death occurs and is capable of detecting premature death [29]. Compared with RD, CVD may cause more premature death. Although pending further confirmation, these results may provide insights for identifying susceptible diseases. Public health studies and resources should be more beneficial if explicitly directed at the major cause of YLL in order to substantially impact general life expectancy [10]. Meanwhile, from the perspective of air pollution control, the effect estimates might help to assess benefits relating to disease burden by reducing the short-term exposure levels (10 μg/m^3^).

Participants′ characteristics such as gender and age were supposed to modify the associations between air pollution and health outcomes. We conducted a subgroup analysis stratified by age and gender and found that most of the adverse effects of ambient air pollution on cause-specific YLL were stronger in females and elderly people than those in males and younger people. Meta-analysis concerning the associations of ambient air pollution with mortality also confirmed these results [6,68]. The heterogeneity of the effects may be attributed to disparities in physiological structures and biological factors. The diameter of the airway in females is narrower, and the levels of immunologic function in females might be lower, than those of males, so may increase the sensitivity of females [69]. Regarding age-specific effects, previous studies show inconsistent results. For instance, the study conducted by Zhu et al. found higher estimates of air pollutant-associated YLL for the elderly than for the young population [29], though opposite results were found in other study [7]. By pooling the effects of these inconsistent results, we confirmed that the air pollutant-associated YLL for the elderly was higher than that for the young population, especially for air pollutant-associated non-accidental specific YLL. The reason for this result may be that preexisting morbid conditions such as decreasing immunologic function and increased allergic sensitization are more prevalent among the elderly [70,71], so elderly people may be more susceptible and vulnerable than younger people. The opposite trends were found for the effects of PM_2.5_ and PM_10_ with the YLL of CVD, but statistically significant associations were found only in elderly people. This discrepancy may be attributed to the small number of studies (N ≤ 3) and other unclear factors. Otherwise, the younger people in our study referred to the whole population, not only adults but also children, based on the 19 included studies. However, none of the included studies were analyzed for the effects of ambient air pollution on the YLL of children separately, so we could not pool the effect estimates for children. As we know, children may be vulnerable, therefore further studies are warranted to clarify the effects of ambient air pollution on the YLL of children. Meanwhile, because of the absence of significant results in younger people, more research related to the association between ambient air pollution and YLL in younger people is needed to determine these trends. Although pending further confirmation, this study might help to shed light on the protecting of susceptible people, and indicated that putting more efforts into their protection and conducting measures to strengthen their knowledge could produce more benefits.

YLL as a surrogate for health has been used in several environmental studies in countries worldwide [10,11,12,13,14,15,16]. However, most efforts have concentrated on the effects of long-term exposure to air pollution on YLL [13,15,16]. To our knowledge, few studies have been conducted to evaluate the effects of short-term exposure to air pollution on cause-specific YLL in other countries than China. A recent study in Tehran focused on the effects of short-term exposure to PM_2.5_ on CVD-specific YLL and found that an interquartile range increase in daily average concentration of PM_2.5_ was associated with 35.21 (95% CI: 10.85–59.58) years increase in CVD-specific YLL [72]. However, in subgroup studies (including gender and age), opposite trends were found when compared with our study. This discrepancy may be attributed to differences in population structure and exposure levels in different countries.

As YLL for each death was calculated by matching age and sex to the extracted life table and daily YLL were obtained by summing the YLL for all deaths on the same day, findings may be dependent on the population size of the studied region [9]. So in regions with large populations there may be more YLL than in those with small population size. To make the results more comparable across studies, population-standardized YLL is more suitable, as in GBD studies [73]. In our study, we also attempted to recalculate the absolute changes in population-standardized YLL for each included study, but failed due to the unavailability of population size for each study. Therefore, our results were expressed as absolute changes of non-standardized YLL related to 10 μg/m^3^ increase in the concentration of ambient air pollutants. As a limitation of our study, this may have contributed to the heterogeneity of this meta-analysis and cause bias in the result to some extents. Although our study did not adjust the confounder of population size, this result may also provide evidence for policy-making, air pollution control, identification of susceptible diseases and susceptible population protection as mentioned above. For further studies on the associations between air pollution and YLL, population standardized YLL are recommended to make the results more comparable and further confirm our results.

The heterogeneity of most pooled effects estimated in our studies was high with I^2^ ≥ 50%. However, limited by the numbers of included studies, we did not conduct analyses such as meta-regression to evaluate the source of heterogeneity. However, sensitivity analysis and trim and fill method were both conducted to assess the robustness of the results. Several factors apart from population size mentioned above may have contributed to heterogeneity in this meta-analysis. First, the associations between ambient air pollution and YLL may be varied by region. In contrast to significant associations in Tianjin [54], no significant associations were observed in Chengdu [28]. This may be partly due to the differences of the ambient air pollution concentrations in the north and south areas of China. The disparities of age and gender structures, which are very important for calculating YLL, might also make important contributions to heterogeneity. Second, the diagnosis classification standard of the outcomes may be another factor. Most of the included studies used ICD10, except one study without an accurate classification standard of diseases (Figure S1) [7]. Third, the differences in the potential confounders adjusted by the studies may also make a contribution to heterogeneity. Although major factors such as temperature, relative humidity, long-term trends and so on were adjusted by most of the studies, insufficient consideration or adjustment will lead to deviation [74]. Fourth, for different studies, we selected different lag days according to the selection standards mentioned above. This may also add to the heterogeneity of the effect estimates. Fifth, limited by the availability of the population data, our results were expressed as absolute changes of non-standardized YLL. Caution should therefore be exercised while interpreting the pooled risk estimates and the need for more air pollution associated population-standardized YLL data is highlighted, in order to increase the certainty of these risk estimates. Overall, more efforts should be made to handle these heterogeneities in order to obtain more evidence.

## 5. Strengths and Limitations

This study has several strengths. First, to the best of our knowledge, this is the first meta-analysis to explore the association between short-term exposure to multiple ambient air pollutants and multiple cause-specific YLL in China. The total number of participants was large, and we not only assessed the effects of ambient PM, but also ambient gaseous pollutants. Furthermore, we estimated the effects of multiple ambient air pollutants on the YLL of more diseases, including non-accidental diseases, CVD, RD, etc. Second, we conducted a quality and risk of bias assessment for each included study based on validated scales used by previous studies [4,9,15,73,75]. In addition, we conducted a sensitivity analysis with no substantially changed summary estimates. These all showed that the pooled estimates were reliable. Thus, our studies may shed light on future research to identify research gaps and provide valuable evidence for governments on air pollution and diseases burden related policy-making. Third, subgroup analysis stratified by age and gender were also considered in this study, which may be helpful in illustrating the heterogeneities of the effects of ambient air pollution on the cause-specific YLL for different age and gender populations.

This study also has limitations. First, the total included number of studies was small, so the statistical power of the heterogeneity test may be limited. Although subsequent studies are required to further confirm the results of this meta-analysis, the quality and risk of bias assessment and sensitivity analysis also attested to the reliability of the estimates to a certain extent. Second, the number of studies for some air pollutants and cause-specific YLL was small (N < 2), which precluded meta-analysis. Third, as several studies did not report the results of multi-pollutant effects, we focused on the effect estimates of a single-pollutant model. Thus, we were unable to identify the interaction effects of multiple air pollutants on YLL. However, evidence reported that co-linearity could confound the model if multiple air pollutants fitting was included, s might lead to the instability of the multi-pollutants model [76]. Fourth, as there was heterogeneity in the cut-off age for different studies included, we focused on the elderly and younger people groups in the subgroup analysis of age rather than a specific age group such as ≥75 years. Further studies should be conducted to assess the exposure-relationship of different fixed ages. Fifth, limited by the number and the geographical coverage of the included studies, the data was nearly all collected from the eastern region of China according to the regional classification standards of the National Bureau of Statistics of China, and only few of the included studies were conducted in the western and central regions [23,27,28]. Thus generalization of these results to the western and central regions of China should be treated with caution. Finally, for lag patterns, various lag days were selected for different studies according to the lag selection standard mentioned above. This approach may lead to a greater pooled effect estimate [4].

## 6. Conclusions

The evidence reviewed in this meta-analysis illustrated that YLL was associated not only with short-term exposure to PM, but also with short-term exposure to gaseous pollutants, especially SO_2_ and NO_2_. The increases in several diseases including non-accidental diseases, CVD, RD, COPD and stroke specific YLL were associated with the short-term exposure to ambient air pollution. The effects were stronger in elderly people and females than those in younger people and males. Our studies may shed light on future research to identify research gaps and provide valuable evidence for governments on air pollution control and diseases burden related policy-making.

## Figures and Tables

**Table 1:** Meta-analysis results for the association between ambient air pollutants and YLL to other diseases in China.

	Air Pollutants	No. of Studies	Effect Estimates (95% CI) (Years)	I^2^ (%)	τ^2^
COPD	PM_2.5_	2	**1.37 (0.24, 2.50) ^R^**	57.6	0.401
	PM_10_	2	**0.50 (0.01, 0.99) ^R^**	67.7	0.088
	O_3_	1	**1.21 (0.12, 2.30)**	—	0.000
IHD	PM_2.5_	1	0.71 (−0.21, 1.64)	—	0.000
	PM_10_	3	**0.84 (0.12, 1.57) ^R^**	86.2	0.349
	SO_2_	2	1.67 (−0.93, 4.27) ^R^	75.6	2.775
	NO_2_	2	1.18 (−0.24, 2.59) ^R^	54.1	0.667
Stroke	PM_10_	3	**0.71 (0.44, 1.00) ^F^**	20.2	0.018
	SO_2_	1	**0.90 (0.10, 1.60)**	—	0.000
	NO_2_	1	**0.60 (0.10, 1.20)**	—	0.000
AMI	PM_2.5_	1	**1.69 (0.01, 3.37)**	—	0.000
	SO_2_	1	**4.97 (0.28, 9.66)**	—	0.000
	NO_2_	1	0.62 (−0.92, 2.17)	—	0.000
	O_3_	1	−0.15 (−1.28, 0.09)	—	0.000
Diabetes mellitus	PM_2.5_	1	0.06 (−0.31, 0.43)	—	0.000
	PM_10_	1	0.01 (−0.29, 0.30)	—	0.000
	SO_2_	1	0.11 (−0.98, 1.20)	—	0.000
	NO_2_	1	0.94 (−0.04, 1.92)	—	0.000

Results are expressed in changes in disease-specific YLL per 10 μg/m^3^ increase in air pollutants with 95% confidence intervals. PM_2.5_: particle with aerodynamic diameter ≤ 2.5 μm; PM_10_: particle with aerodynamic diameter ≤ 2.5 μm; SO_2_: sulfur dioxide; NO_2_: nitrogen dioxide; O_3_: ozone. COPD: chronic obstructive pulmonary disease; AMI: acute myocardial infarction; IHD: ischemic heart disease. CI: confidence intervals; I^2^, I-square statistic; **τ^2^**, tau-squared statistic. ^R^ represents random effects models; ^F^ represents fix effects models. Effect estimates in bold are statistically significant.

**Table 2:** Meta-analysis results for the association of YLL for non-accidental diseases, cardiovascular disease (CVD) and respiratory disease (RD) with ambient air pollution stratified by gender and age in China.

Diseases	Air Pollutants	Subgroup	n	Effect Estimate (95% CI) (Years)	I^2^(%)	τ^2^
Non-accidental diseases	PM_2.5_	Female	3	**1.30 (0.70, 1.90) ^F^**	0.0%	0.000
		Male	3	0.67 (−0.05, 1.39) ^F^	0.0%	0.000
		Younger	3	**1.16 (0.32, 2.00) ^F^**	0.0%	0.000
		Elder	3	**1.80 (0.02, 3.58) ^R^**	83.5%	1.919
	PM_10_	Female	7	**0.97 (0.61, 1.33) ^F^**	28.3%	0.113
		Male	7	**1.48 (0.64, 2.32) ^R^**	58.7%	0.626
		Younger	8	**0.45 (0.16, 0.75) ^R^**	56.2%	0.127
		Elder	8	**0.75 (0.54, 0.97) ^R^**	60.4%	0.082
	SO_2_	Female	5	**4.71 (1.59, 7.83) ^R^**	83.8%	9.104
		Male	5	**4.39 (1.83, 6.96) ^R^**	63.3%	4.477
		Younger	5	**4.58 (1.76, 7.41) ^R^**	58.4%	5.135
		Elder	5	**4.87 (2.23, 7.50) ^R^**	90.4%	6.969
	NO_2_	Female	5	**4.27 (1.93, 6.61) ^R^**	67.1%	4.342
		Male	5	**3.98 (2.57, 5.39) ^F^**	34.6%	1.659
		Younger	5	**4.26 (2.59, 5.93) ^F^**	0.0%	0.000
		Elder	5	**4.33 (1.93, 6.74) ^R^**	85.8%	5.845
CVD	PM_2.5_	Female	2	**0.62 (0.16, 1.09) ^F^**	0.0%	0.000
		Male	2	0.39 (−0.87, 1.65) ^R^	74.6%	0.621
		Younger	2	0.88 (−0.15, 1.91) ^F^	0.0%	0.000
		Elder	2	**0.48 (0.10, 0.86) ^F^**	0.0%	0.000
	PM_10_	Female	3	**0.84 (0.45, 1.23) ^F^**	0.0%	0.000
		Male	3	1.16 (−0.54, 2.87) ^R^	86.9%	1.648
		Younger	3	1.04 (−0.42, 2.50) ^R^	79.4%	1.055
		Elder	3	**0.63 (0.29, 0.96) ^F^**	44.1%	0.092
	SO_2_	Female	2	**2.39 (0.38, 4.39) ^R^**	77.1%	1.652
		Male	2	**1.00 (0.08, 1.91) ^F^**	0.0%	0.000
		Younger	2	**1.32 (0.29, 2.34) ^F^**	0.0%	0.000
		Elder	2	**1.39 (0.82, 1.96) ^F^**	0.0%	0.000
	NO_2_	Female	2	1.95 (−0.32, 4.23) ^R^	86.1%	2.337
		Male	2	**0.75 (0.00, 1.49) ^F^**	5.9%	0.043
		Younger	2	**0.77 (0.38, 1.17) ^F^**	0.0%	0.000
		Elder	2	**1.82 (0.12, 3.51) ^R^**	69.4%	1.077
RD	PM_2.5_	Female	1	0.25 (−0.06, 0.56)	—	0.000
		Male	1	0.07 (−0.33, 0.47)	—	0.000
		Younger	1	−0.01 (−0.35, 0.34)	—	0.000
		Elder	1	0.33 (−0.04, 0.69)	—	0.000
	PM_10_	Female	3	0.42 (−0.00, 0.84) ^R^	77.1%	0.096
		Male	3	0.17 (−0.19, 0.53) ^R^	51.7%	0.047
		Younger	3	0.14 (−0.04, 0.31) ^F^	43.9%	0.025
		Elder	3	0.49 (−0.03, 1.01) ^R^	74.2%	0.128
	SO_2_	Female	1	0.50 (−0.41, 1.41)	—	0.000
		Male	1	0.37 (−0.82, 1.54)	—	0.000
		Younger	1	−0.24 (−1.25, 0.77)	—	0.000
		Elder	1	**1.11 (0.04, 2.18)**	—	0.000
	NO_2_	Female	1	**0.91 (0.09, 1.73)**	—	0.000
		Male	1	0.02 (−1.04, 1.09)	—	0.000
		Younger	1	−0.37 (−1.29, 0.54)	—	0.000
		Elder	1	**1.30 (0.34, 2.27)**	—	0.000

Results are expressed in changes in disease-specific YLL per 10 μg/m^3^ increase in air pollutants with 95% confidence intervals. PM_2.5_: particle with aerodynamic diameter ≤ 2.5 μm; PM_10_: particle with aerodynamic diameter ≤ 2.5 μm; SO_2_: sulfur dioxide; NO_2_: nitrogen dioxide; CI: confidence intervals; I^2^, I-square statistic; **τ^2^**, tau-squared statistic. ^R^ represents random effects models; ^F^ represents fix effects models. Effect estimates in bold are statistically significant.

## Supplementary Materials

The following are available online at https://www.mdpi.com/article/10.3390/ijerph182111467/s1, Figure S1: Characteristics of the included studies according to quality assessments, exposure assessment, health outcomes of YLL, adjustment factors, and other characteristics. Colored grid indicated that these results were expressed by the study. High quality: the study was high quality (score = 5) according to the methodological quality assessments; Low quality: the study was low quality (score < 5) according to the methodological quality assessments. PM_2.5_: particle with aerodynamic diameter ≤2.5 μm; PM_10_: particle with aerodynamic diameter ≤2.5 μm; SO_2_: sulfur dioxide; NO_2_: nitrogen dioxide; _O3_: ozone; RD: respiratory disease; CVD: cardiovascular disease; COPD: chronic obstructive pulmonary disease; AMI: acute myocardial infarction; IHD: Ischemic Heart Disease; ICD: International Classification of Disease, Figure S2: Risk of bias assessment for each study, Figure S3: Forest plot and pooled effect estimates of the ambient air pollution on the YLL of non-accidental disease, Figure S4: Forest plot and pooled effect estimates of the ambient air pollution on the YLL of CVD, Figure S5: Forest plot and pooled effect estimates of the ambient air pollution on the YLL of RD, Figure S6: Forest plot and pooled effect estimates of the ambient air pollution on the YLL of other diseases, Figure S7: Forest plot and pooled effect estimates of the ambient air pollution on YLL for non-accidental diseases, cardiovascular disease (CVD) and respiratory disease (RD) stratified by gender, Figure S8: Forest plot and pooled effect estimates of the ambient air pollution on YLL for non-accidental diseases, cardiovascular disease (CVD) and respiratory disease (RD) stratified by age, Figure S9: Doi plot and LFK index of the ambient air pollution on the disease-specific YLL, Figure S10: Sensitivity analyses of the ambient air pollution on the disease-specific YLL, Table S1: Criteria for the quality assessment of each study basing on the OHAT and Navigation Guide tool, Table S2: The summaries of the included studies in the Meta-analysis. Table S3: The results before and after applying the trim and fill method.
