# Supplementary material for: Years of Life Lost (YLL) Due to Short-Term Exposure to Ambient Air Pollution in China: A Systematic Review and Meta-Analysis
Source: Int J Environ Res Public Health. 2021 Oct 31;18(21):11467. doi: 10.3390/ijerph182111467 (PMC8582650; doi:10.3390/ijerph182111467)
Supplement: Supplementary file 1 [file ijerph-18-11467-s001.zip › ijerph-1433445-supplementary.pdf]

Supplementary Material

Years of life lost (YLL) due to short-term exposure to ambient air pollution in China: a systematic review and meta-analysis

Table of contents:

Part A Search strategies

Part B Standardized method

Part C Selection strategies for the used pooled effect models

Table S1 Criteria for the quality assessment of each study basing on the OHAT and Navigation Guide tool

Table S2 The summaries of the included studies in the Meta-analysis

Table S3 The results before and after applying the trim and fill method

Part B Risk of bias assessment for each of the included studies

Figure S1 Characteristicsof the included studies according to quality assessments, exposure assessment, health outcomes of YLL, adjustment factors, and other characteristics. Colored grid indicated that these results were expressed by the study. High quality: the study was high quality (score=5) according to the methodological quality assessments; Low quality: the study was low quality (score<5) according to the methodological quality assessments. PM2.5: particle with aerodynamic diameter ≤ 2.5μm; PM10: particle with aerodynamic diameter ≤ 2.5μm;

SO2: sulfur dioxide; NO2: nitrogen dioxide; O3: ozone; RD: respiratory disease; CVD: cardiovascular disease; COPD: chronic obstructive pulmonary disease; AMI: acutemyocardialinfarction; IHD: Ischemic Heart Disease; ICD: International Classification of Disease.

Figure S2 Risk of bias assessment for each study

Figure S3 Forest plot and pooled effect estimates of the ambient air pollution on the YLL of non-accidental disease

Figure S4 Forest plot and pooled effect estimates of the ambient air pollution on the YLL of CVD

Figure S5 Forest plot and pooled effect estimates of the ambient air pollution on the YLL of RD

Figure S6 Forest plot and pooled effect estimates of the ambient air pollution on the YLL of other diseases

Figure S7 Forest plot and pooled effect estimates of the ambient air pollution on YLL for non-accidental diseases, cardiovascular disease (CVD) and respiratory disease (RD) stratified by gender

Figure S8 Forest plot and pooled effect estimates of the ambient air pollution on YLL for non-accidental diseases, cardiovascular disease (CVD) and respiratory disease (RD) stratified by age

Figure S9 Doi plot and LFK index of the ambient air pollution on the

disease-specific YLL

Figure S10 Sensitivity analyses of the ambient air pollution on the disease-specific YLL

Part A Search strategies

We searched Pubmed，web of science，medline，Embase，Wan Fang and CNKI databases for literature published in English or Chinese，up to April 2020，using the following terms for titles and abstracts：(“air pollutant” OR “particulate matter” OR “PM10” OR “PM(10)” OR “PM2.5” OR “PM(2.5)” OR “PM” OR “nitrogen dioxide” OR “NO2” OR “NO(2) ” OR “sulfur dioxide” OR “sulphur dioxide” OR “SO2” OR “SO(2) ” OR “ozone” OR “O3” OR “O(3) ” OR “carbon monoxide” OR “CO”) AND (“years of life lost” OR “YLL” OR “burden of disease” OR “disease burden” OR “burden of death”) AND (“China” OR “Chinese”).

The names of some major Chinese locations were also added in the terms. The names of the major Chinese locations were as following: Anshan, Beijing, Chongqing, Foshan, Fuzhou, Guangzhou, Hangzhou, Hong Kong, Kaohsiung, Lanzhou, Nanjing, Quanzhou, Shanghai, Shenyang, Shenzhen, Suzhou, Taichung, Taipei, Taiyuan, Tangshan, Tianjin, Urumqi, Wuhan, Zhongshan, Zhuhai, Anqing, Beihai, Bengbu, Changchun, Changde, Changsha, Changzhou, Chengdu, Dali, Dalian, Dandong, Ganzhou, Guilin, Guiyang, Haikou, Harbin, Hefei, Hohhot, Hsinchu, Huizhou, Jilin, Jinan, Jinhua, Jining, Jinzhou, Jiujiang, Kunming, Luoyang, Luzhou, Macau, Mudanjiang Nanchang, Nanchong, Nanning, Ningbo, Pingdingshan, Qinhuangdao, Sanya, Shaoguan, Shijiazhuang, Tainan, Tangshan, Taoyuan, Wenzhou, Wuxi, Xiamen, Xiangyang, Xining, Xi'an, Xuzhou, Yangzhou, Yantai, Yichang, Yinchuan, Yueyang, Zhanjiang, Zunyi, River delta, and Taiwan.

For the Wan Fang and CNKI databases, we both used the Chinese and English names of the above terms for literature searching.

Part B Standardized method

We standardized the effect estimates with 95%CI that extracted from each study to a 10μg/m³ change as the increasing unitusing the equation as follows:

$$\text{ES}_{Standardized} = \frac{ES_{Origin}}{Increment_{Origin}} \times Increment_{Standardized}$$

Where $\text{ES}_{Standardized}$ is the standardized effect estimates; ES_{Origin} is the original effect estimates that extracted from each study; $Increment_{Origin}$ is the original increment of air pollution that extracted from each study; $Increment_{Standardized}$ is the standardized increment of air pollution, in our study, we used 10 μ g/m³.

The standard error of each study was calculated according to the effect estimates with 95%CI that extracted from each study to a 10μg/m³ change as the increasing unit using the equation as follows:

$$SE_{Standardized} = \frac{UCI_{Origin} - ES_{Origin}}{Increment_{Origin} \times 1.96} \times Increment_{Standardized}$$

Where$SE_{Standardized}$ is the standard error of the standardized effect estimate; $UCI_{Origin}$is the upper confidence interval of original effect estimate that extracted from each study..

Part C Selection strategies for the used pooled effect models

We chose the pooled effects model according to the p-values of the I^2-based Cochran Q testand I^2 metrics. If $P<0.05$ or $I^2 \geqslant 50\%$, it indicates that there is a larger heterogeneity between the studies, we chose the random-effects model to calculate the pooled effect estimates. If $P \geqslant 0.05$and$I^2<50\%$, the fixed-effects modelswere used to calculate the pooled effect estimates.

Table S1 Criteria used for the quality assessment of each study, according to the OHAT and Navigation Guide tool[1]

Bias criteria		Risk of Bias Question/Domains	Answer
**Key criteria**	**Exposure assessment**	Includes measurement error or measurement limitations.<br><br>List of major considerations:<br>1) air pollution measurements were performed daily,<br>2) < 25% missing data,<br>3) more than one monitoring station per a large geographical area	-LOW risk: There is high confidence that the exposure to PM and its constituents is the true average population exposure.<br><br>-PROBABLY LOW: There is indirect evidence that suggests low risk of bias, or one of the three listed considerations is not applied.<br><br>-PROBABLY HIGH risk: There is insufficient information to permit a judgment of high risk of bias, but there is indirect evidence that suggests t high risk of bias. Additionally, two out of the three listed considerations are not applied.<br><br>-HIGH risk: There is direct evidence of high risk of misclassification bias, or all three of the listed considerations are not applied.<br><br>-NOT APPLICABLE: exposure assessment methods of study are not expected to introduce bias in the study
	**Outcome**	Outcome assessment	-LOW risk: mortality cause classified based on diagnosis standard criteria (International Classification System – ICD code) and provided

Bias criteria		Risk of Bias Question/Domains	Answer
	**assessment**	methods lack accuracy	by a National or RegionalDatabase.<br><br>-PROBABLY LOW: outcome was assessed based on diagnosis standard criteriaand collected by researcher<br><br>-PROBABLY HIGH risk: outcome was not assessed based on standard diagnosis criteria AND is accompanied by validation sub-study or sensitivity analysis to suggest that the risk is minimum.<br><br>-HIGH risk: Outcome was assessed based on self-reports (parents, family) and data collected by the researcher.
	**Confounding bias**	Study appropriately accounted for all important well studied potential confounders (long-term trends, seasonality, day of week, temperature,	-LOW risk: study accounted for all important confounders which were measured consistently<br><br>-PROBABLY LOW: study accounted for most of confounders AND is not expected to introduce bias

Bias criteria		Risk of Bias Question/Domains	Answer
		relative humidity)	-PROBABLY HIGH risk: study accounted for some but not all of confounders AND is expected to introduce bias<br><br>-HIGH risk: study did not account for potential confounders OR were inappropriately measured<br><br>-NOT APPLICABLE: assessment of outcome cannot introduce bias in the study
**Other criteria**	**Selection bias**	Does the selection of participants into the study was done in a manner that might introduce bias in the study? e.g. study only certain days (for examples, only Fridays), and not all days, seasons were included.	-LOW risk: The descriptions of the studied population were sufficiently detailed to support the assertion that risk of selection effects was minimal.<br><br>-PROBABLY LOW risk: There is insufficient information about population selection to permit a judgment of low risk of bias, but there is indirect evidence that suggests low risk of bias.<br><br>-PROBABLY HIGH risk: There is insufficient information about population selection to permit a judgment of high risk of bias, but there

Bias criteria		Risk of Bias Question/Domains	Answer
			is indirect evidence that suggests high risk of bias.<br><br>- HIGH risk: There were indications from descriptions of the studied population of high risk of bias.<br><br>- NOT APPLICABLE: There is evidence that selection is not an element of study design capable of introducing risk of bias in the study.
	**Incomplete outcome data**	Was incomplete data adequately addressed?	-LOW risk: no missing outcome data or missing data is unrelated to true outcome<br><br>-PROBABLY LOW: there was insufficient information about incomplete data to judge for low risk, but indirect evidence that suggests low risk of bias<br><br>-PROBABLY HIGH risk: there was insufficient information about incomplete data to judge for high risk, but indirect evidence that suggests high risk

Bias criteria		Risk of Bias Question/Domains	Answer
			-HIGH risk: missing outcome data is related to true outcome<br><br>-NOT APPLICABLE: there is evidence that incomplete outcome data is not introducing bias in the study
	**Selective reporting**	Selective reporting of outcomes or analyses.	-LOW risk: all of the studies pre-specified outcomes and findings are reported<br><br>-PROBABLY LOW: there was insufficient information about selective outcome to judge for low risk, but indirect evidence that suggests study was free of selective report<br><br>-PROBABLY HIGH risk: there was insufficient information about selective reporting to judge for high risk, but indirect evidence suggests that study was not free of selective reporting<br><br>-HIGH risk: not all pre-specified outcomes and findings were reported,

Bias criteria		Risk of Bias Question/Domains	Answer
			or one/more of the primary outcomes or analyses were assessed or executed with other methods than the pre-specified one, or one/more of the reported outcomes/findings was/were not pre-specified<br><br>-NOT APPLICABLE: there is evidence that selective outcome reporting is not introducing bias in the study
	**Conflict of interest**	Potential source of bias in reporting through source of funding	-LOW risk: the study did not receive funding from an entity with financial interest in the outcome of study<br><br>-PROBABLY LOW: there is insufficient information to judge for low risk, but indirect evidence suggests study was free of financial interest<br><br>-PROBABLY HIGH risk: there is insufficient information to judge for high risk, but indirect evidence suggests study was not free of financial interest

Bias criteria		Risk of Bias Question/Domains	Answer
			-HIGH risk: study received support from an entity with financial interest in the outcome of study
	**Other**	Bias due to other problems not covered elsewhere	-LOW risk: No other sources of bias<br><br>-PROBABLY LOW: there is insufficient information to judge for low risk, but indirect evidence suggests study was free of other problems<br><br>-PROBABLY HIGH risk: there is insufficient information to judge for high risk, but indirect evidence suggests study was not free of other problems<br><br>-HIGH risk: at least one important risk of bias

1. Achilleos, S.; Kioumourtzoglou, M. A.; Wu, C. D.; Schwartz, J. D.; Koutrakis, P.; Papatheodorou, S. I., Acute effects of fine particulate matter constituents on mortality: A systematic review and meta-regression analysis. *Environ Int* **2017,** *109*, 89-100.

Table S2 The summaries of the included studies in the Meta-analysis

Study	Location	Study period	Type of study	studied deaths size	Exposure	Source of exposure	Health outcome of YLL	Diagnosis standard	YLL calculate method	Adjustment factors	Lag paterns	Stratified analysis	Main findings
Guo et al. 2013	8 urban districts in Beijing	2004 -2008	Time-series study	80515 deaths	$PM_{2.5}$, PM_{10}, SO_2, NO_2	$PM_{2.5}$ was monitored at the main campus of Peking University; PM_{10}, SO_2, NO_2 were from eight fixed monitoring sites distributed in different part of the urban area from Beijing municipal environmental	Non-accidental disease	The Beijing death classification system	WHO life tables	Long-term trends, seasonality, the day of week, temperature, relative humidity, air pressure	1.Moving average concentration over two days (Lag01) (main analysis); 2.Single day lag: lag0, lag1, lag2, lag3	1.Gender: female and male; 2.Age: ≤65years and >65years	1. An IQR increase in $PM_{2.5}$ (94μg/m³),PM_{10}(106μg/m³),SO_2(49μg/m³) and NO_2 (30μg/m³)in lag01 was related to YLL increases of 15.8 (95%CI:5.3-26.3), 15.8(95%CI:6.1-25.5), 16.2 (95%CI:4.1-28.4) and 15.1(95%CI:4.7-25.6) years. 2. An IQR increase in $PM_{2.5}$,PM_{10},SO_2 and NO_2 in lag01 was related to YLL increases of 11.1 (95%CI:4.7-17.5), 9.3(95%CI:3.3-15.2), 5.6(95%CI:-1.9-13.1), 6.7(95%CI:0.3-13.1) years for female and 4.7 (95%CI:-2.9-12.3), 6.5 (95%CI: -0.5-13.5), 10.6 (95%CI:1.8-19.4), 8.4 (95%CI:0.8-16.0) years for male . 3. An IQR increase in $PM_{2.5}$,PM_{10},SO_2 and NO_2 in lag01

Study	Location	Study period	Type of study	studied deaths size	Exposure	Source of exposure	Health outcome of YLL	Diagnosis standard	YLL calculate method	Adjustment factors	Lag paterns	Stratified analysis	Main findings
						monitoring centre							was related to YLL increases of 12.0 (95%CI:2.9- 21.0), 10.3 (95%CI:2.0-18.6), 10.8 (95%CI:0.3-21.3), 10.1 (95%CI:1.1-19.1) years for the youngers and 3.8 (95%CI:-0.9-8.6), 5.5 (95%CI:1.1-9.9), 5.4 (95%CI:-0.1-10.9), 5.0 (95%CI:0.3-9.8) years for the elders .

Study	Location	Study period	Type of study	studied deaths size	Exposure	Source of exposure	Health outcome of YLL	Diagnosis standard	YLL calculate method	Adjustment factors	Lag paterns	Stratified analysis	Main findings
Lu et al. 2015	9 urban districts in Nanjing	2009-2013	Time-series study	147956 deaths	PM_{10}, SO_2, NO_2	From 9 state-controlled monitoring stations from the Nanjing environmental monitoring center	Non-accidental disease	ICD 10: A00-R99	Coale-Demeny West model life table	Long-term trends, seasonal trends, temperature, relative humidity, barometric pressure, day of the week, public holidays	Lag01(main analysis), lag02, lag03;<br>Lag0-lag5	1.Gender: female and male;<br>2.Age: ≤75years and >75years	1. An IQR increase in PM_{10}(66.3 μg/m³),SO_2(23.3μg/m³) and NO_2 (25.3μg/m³)in lag01 was related to YLL increases of 20.5 (95%CI: 6.3-34.8), 30.3(95%CI: 12.2-48.4), 34.9 (95%CI:16.9-52.9) years.<br>2. An IQR increase in PM_{10},SO_2 and NO_2 in lag01 was related to YLL increases of5.3 (95%CI:-3.5- 14.0), 10.2(95%CI:-0.9-21.4), 13.5(95%CI: 2.4-24.6) years for female and 15.3 (95%CI:4.7-25.9), 20.1 (95%CI: 6.6-33.5), 21.4 (95%CI:8.0-34.8) years for male .<br>3. An IQR increase in PM_{10},SO_2 and NO_2 in lag01 was related to YLL increases of 11.4 (95%CI:-1.4- 24.1), 19.6 (95%CI:3.5-35.8), 20.0 (95%CI: 3.9-36.1) years for the youngers and 9.2 (95%CI:4.2-14.2), 10.7 (95%CI: 4.3- 17.0), 14.9 (95%CI:8.6-21.2) years for the elders .

Study	Location	Study period	Type of study	studied deaths size	Exposure	Source of exposure	Health outcome of YLL	Diagnosis standard	YLL calculate method	Adjustment factors	Lag paterns	Stratified analysis	Main findings
He et al. 2016	Ningbo	2009-2013	Time-series study	163704 deaths	$PM_{2.5}$, PM_{10}, SO_2, NO_2	From 11 monitoring sites that cover all districts and counties of Ningbo, from Environment monitoring center of Ningbo	Non-accidental disease	ICD10	WHO life tables	Secular trends, seasonality, temperature, relatively humidity, air pressure, wind speed, day of week	Lag03 (main analysis);<br>lag0-lag06	1.Gender: female and male;<br>2.Age: ≤65years and >65years	1. A 10μg/m³ increase in $PM_{2.5}$,PM_{10},SO_2 and NO_2in lag03 was related to YLL increases of 2.97 (95%CI:-2.01-7.95), 4.27(95%CI: 1.17-7.38), 29.98 (95%CI: 19.21-40.76) and 16.58(95%CI:8.19-24.97) years.<br>2. A10μg/m³ increase in $PM_{2.5}$,PM_{10},SO_2 and NO_2 in lag03 was related to YLL increases of 0.71 (95%CI:-2.36- 3.79), 1.94(95%CI:0.02-3.86), 17.19 (95%CI:10.55-23.83), 8.67(95%CI: 3.51-13.85) years for female and 2.26 (95%CI:-1.61-6.13), 2.33 (95%CI: -0.01-4.68), 12.79 (95%CI:4.62-20.97), 7.90 (95%CI:1.57-14.24) years for male .<br>3. A 10μg/m³ increase in $PM_{2.5}$,PM_{10},SO_2 and NO_2 in lag03 was related to YLL increases of -1.18 (95%CI:-5.34- 2298), 1.32 (95%CI:

Study	Location	Study period	Type of study	studied deaths size	Exposure	Source of exposure	Health outcome of YLL	Diagnosis standard	YLL calculate method	Adjustment factors	Lag paterns	Stratified analysis	Main findings
													-1.25-3.88), 14.23 (95%CI:5.31-23.15), 7.33 (95%CI:0.41- 14.25) years for the youngers and 4.15 (95%CI:1.62-6.68), 2.96 (95%CI: 1.40- 4.52), 15.75 (95%CI:10.34-21.16), 9.25 (95%CI:5.04-13.46) years for the elders .
Yang et al. 2016	6 central urban districts in Guangzhou	2004-2007	Time-series study	——	PM_{10}, SO_2, NO_2	From Guangzhou Bureau of Environmental Protection	Non-accidental disease, cardiovascular disease, ischemic heart disease, stroke	ICD-10: A00-R99, I00-I99, I20-I25, I60-I69	WHO life tables	Long-term trends, seasonality, temperature, relative humidity, atmospheric pressure, day of	Lag01 (main analysis), Lag04; Lag0-lag4	1.Gender: female and male; 2.Age: <75years and ≥75years	1. For non-accidental diseases, a 10μg/m³ increase in PM_{10},SO_2 and NO_2in lag01 was related to YLL increases of 6.5(95%CI: 3.8-9.1), 4.4 (95%CI: 1.8-7.1) and 5.3(95%CI:2.4-8.1) years. For CVD, a 10μg/m³ increase in PM_{10},SO_2 and NO_2in lag01 was related to YLL increases of 2.8(95%CI: 1.7-3.8), 2.6 (95%CI: 1.2-4.0) and 1.8(95%CI:0.8- 2.9) years. 2. For non-accidental diseases, a 10μg/m³ increase inPM_{10},SO_2 and NO_2 in lag01 was related to YLL

Study	Location	Study period	Type of study	studied deaths size	Exposure	Source of exposure	Health outcome of YLL	Diagnosis standard	YLL calculate method	Adjustment factors	Lag paterns	Stratified analysis	Main findings
										the week, public holidays			increases of2.8 (95%CI:1.3-4.4), 2.0(95%CI:0.4-3.5), 1.9(95%CI: 0.3-3.6) years for female and3.6(95%CI: 1.7-5.5), 2.5(95%CI:0.5- 4.4), 3.3 (95%CI:1.3-5.4) years for male . For CVD, a 10μg/m³ increase inPM_{10},SO_2 and NO_2 in lag01 was related to YLL increases of 1.1 (95%CI:0.4-1.8), 1.5(95%CI: 0.7-2.4), 0.9(95%CI: 0.3-1.6) years for female and 1.7 (95%CI: 0.9-2.5), 1.1(95%CI:0.1- 2.1), 0.9 (95%CI: 0.1-1.7) years for male .<br><br>3. For non-accidental diseases, a 10μg/m³ increase inPM_{10},SO_2 and NO_2 in lag01 was related to YLL increases of 4.9 (95%CI:2.5-7.3),2.8(95%CI:0.4-5.2), 3.8(95%CI:1.3-6.3) years for the youngers and 1.6(95%CI:0.8-2.4),1.6(95%CI:0.8-2.4),1.5(95%CI:0.6-2.3) years for the

Study	Location	Study period	Type of study	studied deaths size	Exposure	Source of exposure	Health outcome of YLL	Diagnosis standard	YLL calculate method	Adjustment factors	Lag paterns	Stratified analysis	Main findings
													elders. For CVD, a 10μg/m³ increase inPM_{10},SO_2 and NO_2 in lag01 was related to YLL increases of 1.9(95% CI:1.1-2.8),1.2(95%CI:0.1-2.4),1.1(95%CI:0.3-2.0)years for the youngers and 0.8(95%CI:0.3-1.2), 1.3(95%CI: 0.7-1.9), 0.8(95%CI: 0.4-1.2) years for the elders .
Chen et al. 2017	Chengdu	2010-2014	Time-series study	366353 deaths	PM_{10}	From 12 monitoring stations of Sichuan Environmental Monitoring Center	Non-accidental disease, respiratory disease, cardiovascular disease	ICD-10: A00-R99, J00-J99, I00-I99	Abridged life table	Long-term trends, seasonality, temperature, relative humidity, air pressure, wind speed, day of	Lag0-lag5	1.Gender: female and male; 2.Age: <60years and ≥60years	1. A 1μg/m³increase in $PM_{10}$was related to YLL increases of 0.26893 (95%CI:-1.34023-1.87809) for non-accidental diseases, 0.30437 (95%CI:-0.16546-0.77421) for RD, 0.21924(95%CI:-0.32917-0.76765) for CVD. 2. A 1μg/m³increase in $PM_{10}$was related to YLL increases of 0.01711 (95%CI:-1.43261-1.46683) in female and 0.85985(95%CI:-0.38123 -2.10094), 0.05980(95%CI: -0.19997-0.24774) in the youngers and 0.14495 (95%CI: -0.00285-

Study	Location	Study period	Type of study	studied deaths size	Exposure	Source of exposure	Health outcome of YLL	Diagnosis standard	YLL calculate method	Adjustment factors	Lag paterns	Stratified analysis	Main findings
										the week			0.23071) in the elders for non-accidental diseases, 0.08367(95%CI: -0.00906-0.17640) in females and 0.31315(95%CI:-0.04772-0.67402) in males, 0.15029(95%CI: -0.87797 -0.60053) in the youngers and 0.22671(95%CI:0.00902-0.39550) in the elders for RD, 0.00445(95%CI: -0.35882-0.36779) in females and 0.33841(95%CI:-0.01519-0.69201) in males, 0.10349(95%CI:-0.73862-0.29345) in the youngers and 0.26600 (95%CI:-0.27786-0.31021) in the elders for CVD.
Li et al. 2017	Ningbo	2011-2015	Time-series study	18472 COPD deaths	$PM_{2.5}$, PM_{10}	From 11 monitoring sites from the Environmental Monitoring Center of	Chronic obstructive pulmonary desease	ICD-10: J40-44	WHO life table	Secular trends, seasonality, temperature, relatively	Lag04 (main analysis), Lag01-lag07; Lag0-lag7	1.Gender: female and male; 2.Age: <75years and ≥75years	1. A 10μg/m³ increase in $PM_{2.5}$,PM_{10}in lag04 was related to YLL increases of 0.91 (95%CI:0.16-1.66), 0.81 (95%CI:0.30-1.33) years. 2. A10μg/m³ increase in $PM_{2.5}$ in lag04 was related to YLL increases of 0.45 (95%CI:-0.04-0.94)years for

Study	Location	Study period	Type of study	studied deaths size	Exposure	Source of exposure	Health outcome of YLL	Diagnosis standard	YLL calculate method	Adjustment factors	Lag paterns	Stratified analysis	Main findings
						Ningbo				humidity, day of week			female and 0.47 (95%CI:-0.08-1.01) years for male .<br>3. A10μg/m³ increase in $PM_{2.5}$ in lag04 was related to YLL increases of -0.06 (95%CI:-0.54-0.42)years for the youngers and 0.98 (95%CI: 0.42-1.54) years for the elders .
Ni et al. 2017a	6 urban districts in Tianjin	2001-2010	Time-series study	——	NO_2	From 6 monitoring sites from the Environmental Monitoring Center of Tianjin	Non-accidental disease	ICD-10: A00-R99	WHO life table	Long-term trends, seasonality, the day of week, temperature, relative humidity	Lag 0-lag1; Lag 01	——	1. A 10μg/m³ increase in NO_2in lag01 was related to YLL increases of 1.62 (95%CI:0.69-2.54) years.

Study	Location	Study period	Type of study	studied deaths size	Exposure	Source of exposure	Health outcome of YLL	Diagnosis standard	YLL calculate method	Adjustment factors	Lag paterns	Stratified analysis	Main findings
Ni et al. 2017b	Nankai district in Tianjin	2001-2010	Time-series study	53554 deaths, 27290 cardiovascular deaths, 4597 respiratory deaths	SO_2	From 1 monitoring sites from the Environmental Monitoring Center of Tianjin	Non-accidental disease, cardiovascular disease, respiratory disease	ICD-10: A00-R99, I00-I99, J00-J99	WHO life tables	Long-term trends, seasonality, the day of week, temperature, relative humidity	Lag 0-lag1; Lag 01	——	1. A 10μg/m³ increase in SO_2in lag01 was related to YLL increases of 1.38 (95%CI:0.43-2.34) years for non-accidental diseases, 0.56(95%CI:-0.07-1.18) for CVD, 0.17(95%CI:-0.05-0.38) for RD.
Zeng et al. 2017a	6 urban districts in Tianjin	2001-2010	Time-series study	270277 deaths	PM_{10}	From 6 monitoring sites from the Environmental Monitoring Center of Tianjin	Non-accidental disease	ICD-10: A00-R99	WHO life table	Long-term trends, seasonality, the day of week, temperature, relative humidity	Lag 0-lag1; Lag 01	1.Gender: female and male; 2.Age: <65years and ≥65years	1. A 10μg/m³ increase in PM_{10}in lag01 was related to YLL increases of 0.4(95%CI:-0.0-0.9), 0.3(95%CI:-0.2-0.8), -0.0(95%CI:-0.5-0.5),0.5 (95% CI: 0.1-1.0), 0.2(95%CI:-0.1-0.5), -0.1 (95%CI:-0.6-0.3) for the youngers and 0.6(95%CI:0.3-1.0), 0.7(95%CI:0.3-1.1),0.5(95%CI:0.1-0.9),0.9(95%CI:0.5-1.3),0.3(95%CI:0.0-0.6), 0.6(95%CI:0.2-1.0) for the elders.

Study	Location	Study period	Type of study	studied deaths size	Exposure	Source of exposure	Health outcome of YLL	Diagnosis standard	YLL calculate method	Adjustment factors	Lag paterns	Stratified analysis	Main findings
Zeng et al. 2017b	6 urban districts in Tianjin	2001-2010	Time-series study	25908 respiratory deaths	PM_{10}	From 6 monitoring sites from the Environmental Monitoring Center of Tianjin	Respiratory disease	ICD-10	WHO life table	Long-term trends, seasonality, the day of week, temperature, relative humidity	Lag 0-lag1; Lag 01	1.Gender: female and male; 2.Age: <65years and ≥65years	1.A 10μg/m³ increase in PM_{10}in lag01 was related to YLL increases of 0.84 (95%CI:0.45-1.23) years. 2. A 10μg/m³ increase in PM_{10}in lag01 was related to YLL increases of 0.59 (95%CI:0.34-0.83) for females and 0.26 (95%CI:-0.01-0.52) for males. 3. A 10μg/m³ increase in PM_{10}in lag01 was related to YLL increases of 0.26(95%CI:0.03-0.48) for the youngers and 0.59 (95%CI:0.28-0.89) for the elders.
Zhang et al. 2017	2 urban districts in Wuhan	2009-2012	Time-series study	——	PM_{10}	From Wuhan Environmental Monitoring Center	Non-accidental disease, cardiovascular disease, respiratory	ICD-10: A00-R99, I00-I99, J00-J99, I60-I69, I20-I25	WHO life table	Long-term trends, seasonality, the day of week,	Lag 01 (main analysis); Lag0-lag3; Lag01-lag03	1.Gender: female and male; 2.Age: <75years and	1.A 10μg/m³ increase in PM_{10}in lag01 was related to YLL increases of 1.23 (95%CI:0.15-2.31) for non-accidental diseases, 0.88(95%CI: 0.36-1.40) for CVD, 0.18(95%CI: -0.04-0.41) for RD. 2. A 10μg/m³ increase in PM_{10}in

Study	Location	Study period	Type of study	studied deaths size	Exposure	Source of exposure	Health outcome of YLL	Diagnosis standard	YLL calculate method	Adjustment factors	Lag paterns	Stratified analysis	Main findings
							disease<br>Stroke, ischemic heart disease			temperature, relative humidity, public holiday		≥75years	lag01 was related to YLL increases of 0.59 (95%CI:-0.04-1.23) for females, 0.63(95%CI: -0.21-1.48) for males, 0.77(95%CI: -0.25-1.79) for the youngers, 0.46 (95%CI: 0.14-0.77) for the elders.
Zhu et al. 2017	Wuxi	2012-2015	Time-series study	117418 deaths	$PM_{2.5}$, PM_{10}, SO_2, NO_2	From 7 state-controlled monitoring stations from the Wuxi Municipal Environmental Monitoring Center	Non-accidental disease, cardiovascular disease, respiratory disease<br>Diabetes mellitus	ICD-10: A00-R99, I00-I99, J00-J99,<br>E10-E14	WHO life table	Time, the day of week, temperature, relative humidity	Lag02 (main analysis);<br>lag0-lag6	1.Gender: female and male;<br>2.Age: ≤75years and >75years	1. An IQR increase in $PM_{2.5}$(39 μg/m³)PM_{10}(56 μg/m³),SO_2(23 μg/m³) and NO_2 (21μg/m³)in lag02 was related to YLL increases of 12.09 (95%CI: 2.98-21.2), 13.69(95%CI: 3.32-24.07), 24.39 (95%CI: 8.62 -40.15), 26.95 (95%CI: 13.99 -39.91) years.<br>2. For females, an IQR increase in$PM_{2.5}$, PM_{10},SO_2 and NO_2 in lag02 was related to YLL increases of 7.57 (95%CI:1.99-13.14), 7.67(95%CI: 1.32-14.02), 12.80(95%CI:3.14-22.45),14.64(95%CI:6.70-22.58) years for non-accidental diseases, 3.04(95%CI:0.72-5.37), 4.10(95%

Study	Location	Study period	Type of study	studied deaths size	Exposure	Source of exposure	Health outcome of YLL	Diagnosis standard	YLL calculate method	Adjustment factors	Lag paterns	Stratified analysis	Main findings
													CI:1.46-6.75), 8.20(95%CI: 4.19-12.22), 6.79(95%CI:3.48-10.10) years for CVD, 0.98(95%CI:0.72-5.37), 0.58(95%CI:-0.80-1.96), 1.16 (95%CI:-0.94-3.25), 1.91(95%CI: 0.18-3.63) years for RD. For males, an IQR increase in$PM_{2.5}$, PM_{10},SO_2 and NO_2 in lag02 was related to YLL increases of 4.53(95%CI:-2.26-11.31), 6.02(95%CI:-1.71-13.75), 11.69(95%CI:-0.16-23.34), 12.31 (95%CI:2.63-21.99) years for non-accidental diseases, -0.83(95% CI:-3.84-2.19), -0.81(95%CI:-4.25-2.62),1.09(95%CI:-4.13-6.32), -0.54 (95%CI:-4.85-3.76) years for CVD, 0.28(95%CI:-1.29-1.84), 0.02(95% CI:-1.76-1.81), 0.84(95%CI:-1.88-3.55),0.04(95%CI:-2.19-2.28) years for RD.<br><br>3. For the youngers, an IQR increase in$PM_{2.5}$, PM_{10},SO_2 and NO_2 in lag02 was related to YLL increases of 4.62

Study	Location	Study period	Type of study	studied deaths size	Exposure	Source of exposure	Health outcome of YLL	Diagnosis standard	YLL calculate method	Adjustment factors	Lag paterns	Stratified analysis	Main findings
													(95%CI:-2.68-11.91), 6.18(95%CI: -2.12-14.49), 11.86(95%CI:-0.77-24.49), 10.15(95%CI:-0.26-20.55) years for non-accidental diseases, 7.34(95%CI:-21.74-38.71), 1.37(95% CI:-2.01-4.74), 4.04(95%CI:-1.10-9.17), 0.24(95%CI:-3.99-4.47) years for CVD, -0.02(95%CI:-1.36-1.32), -0.28(95%CI:-1.80-1.25), -0.56(95% CI:-2.88-1.76), -0.78(95%CI:-2.70-1.13) years for RD. For the elders, an IQR increase in$PM_{2.5,}$ PM_{10},SO_2 and NO_2 in lag02 was related to YLL increases of 7.48(95%CI:2.61-12.34), 7.51(95%CI:1.97-13.05), 12.53(95% CI:4.09-20.96),16.81(95%CI:9.89-23.72) years for non-accidental diseases, 1.49(95%CI:-1.04-4.02), 1.92(95%CI:-0.95-4.80), 5.26(95% CI:0.89-9.63), 6.01(95%CI:2.41-9.60) years for CVD, 1.28(95%CI: -0.14-2.71), 0.88(95%CI:-0.74-2.50), 2.56(95%CI:0.10-5.02), 2.73(95%

Study	Location	Study period	Type of study	studied deaths size	Exposure	Source of exposure	Health outcome of YLL	Diagnosis standard	YLL calculate method	Adjustment factors	Lag paterns	Stratified analysis	Main findings
													CI:0.71-4.76) years for RD.
Huang et al. 2018a	Ningbo	2011-2015	Time-series study	10322 ischemic heart deaths	$PM_{2.5}$, SO_2, NO_2	From 8 monitoring sites from the Environmental Monitoring Center of Ningbo	Ischemic heart disease	ICD-10: I20-I25	WHO life table	Secular trends, day of the week, temperature, relative humidity,	Lag 3 (main analysis); Lag0-lag7, lag01-lag07	1.Gender: female and male; 2.Age: <75years and ≥75years	1. A 10μg/m³ increase in $PM_{2.5}$, SO_2, NO_2 in lag3 was related to YLL increases of 0.71 (95%CI:-0.21-1.64), 3.31(95%CI:0.78-5.84), 2.27 (95%CI:0.26-4.28) years. 2. A 10μg/m³ increase in $PM_{2.5}$, SO_2, NO_2 in lag3 was related to YLL increases of 0.34 (95%CI:-0.71-0.86), 0.43(95%CI:-0.98-1.83), 0.08 (95%CI:-1.01-1.20) years for the females, 0.37(95%CI:-0.39-1.13), 2.88(95%CI:0.80-4.97), 2.19(95% CI:0.83-3.84) years for the males. 3. A 10μg/m³ increase in $PM_{2.5}$, SO_2, NO_2 in lag3 was related to YLL increases of 0.28 (95%CI:-0.55-1.10), 1.90(95%CI:-0.35-4.16), 1.85 (95%CI:0.06-3.64) years for the youngers, 0.44(95%CI:-0.01-1.13), 1.41(95%CI:0.18-2.64), 0.42(95%

Study	Location	Study period	Type of study	studied deaths size	Exposure	Source of exposure	Health outcome of YLL	Diagnosis standard	YLL calculate method	Adjustment factors	Lag paterns	Stratified analysis	Main findings
													CI:-0.56-1.40) years for the elders.
Huang et a. 2018b	Ningbo	2011-2015	Time-series study	18472 chronic obstructive pulmonary disease deaths	O_3	From 8 monitoring sites from the Environmental Monitoring Center of Ningbo	Chronic obstructive pulmonary disease	ICD-10: J40-J44	WHO life table	Secular trend, seasonality, temperature, relative humidity, day of the week	Lag02 (main analysis); Lag0-lag5; Lag01-lag02	1.Gender: female and male; 2.Age: <75years and ≥75years	1. A 10μg/m³ increase in $PM_{2.5}$in lag02 was related to YLL increases of 2.09 (95%CI:0.78-3.39) years. 2. A 10ppb increase in O_3in lag02 was related to YLL increases of 2.37 (95%CI:0.23-4.50) years.
Liang et al. 2018	Hong Kong	2011-2015	Time-series study	9252 acute myocardial infraction deaths	$PM_{2.5}$, SO_2, NO_2, O_3	From 14 monitoring stations from the Hong Kong Environmental Protection Department	acute myocardial infraction disease	ICD-10: I21	Hong Kong Census and Statistics Departments life table	Secular trend, seasonality, temperature, relative humidity, day of the week,	Lag01 (main analysis) Lag0-lag3;	1.Gender: female and male; 2.Age: ≤65years and >65years	1. A 10μg/m³ increase in $PM_{2.5}$, SO_2, NO_2, O_3in lag01 was related to YLL increases of 1.69 (95%CI:0.01-3.37), 4.97(95%CI:0.28-9.66), 0.62(95% CI:-0.92-2.17), -0.15(95%CI:-1.28-0.99) years. 2. A 10μg/m³ increase in $PM_{2.5}$in lag01 was related to YLL increases of 0.68 (95%CI:-0.19-1.55) years for females and 1.01 (95%CI:-0.39-2.41)

Study	Location	Study period	Type of study	studied deaths size	Exposure	Source of exposure	Health outcome of YLL	Diagnosis standard	YLL calculate method	Adjustment factors	Lag paterns	Stratified analysis	Main findings
										public holiday			years for the males.<br>3. A 10μg/m³ increase in $PM_{2.5}$in lag01 was related to YLL increases of 0.63(95%CI:-0.74-2.01) years for the youngers and 0.93 (95%CI:-0.01-1.87) years for the elders.
Zeng et al. 2018a	Tianjin	2001-2010	Time-series study	——	PM_{10}	From 6 monitoring sites from the Environmental Monitoring Center of Tianjin	Cardiovascular disease;<br>stroke<br>Ischemic heart disease	ICD-10:<br>I00-I99;<br>I60-I69;<br>I20-I25	WHO life table	Time, the day of week, temperature, relative humidity	Lag0-lag1;<br>Lag01	——	1. A 10μg/m³ increase in PM_{10}in lag01 was related to YLL increases of 2.86 (95%CI:1.79-3.93) years for the CVD, 1.07(95%CI:0.43-1.71) years for the stroke, 1.59(95%CI:0.95-2.23) years for the IHD.
Zeng et al. 2018b	6 urban districts in Tianjin	2001-2010	Time-series study	——	PM_{10}	From 6 monitoring sites from the Environmental Monitoring	Chronic obstructive pulmonary disease	ICD-10: J40-J44	WHO life table	Long-term trends, seasonality, dayof the week,	Lag0-lag1;<br>Lag01	1.Gender: female and male;<br>2.Age: <65years and ≥	1. A 10μg/m³ increase in PM_{10}in lag01 was related to YLL increases of 0.30 (95%CI:0.06-0.54) years.<br>2. A 10μg/m³ increase in PM_{10}in lag01 was related to YLL increases of0.10(95%CI:-0.06-0.27) years for

Study	Location	Study period	Type of study	studied deaths size	Exposure	Source of exposure	Health outcome of YLL	Diagnosis standard	YLL calculate method	Adjustment factors	Lag paterns	Stratified analysis	Main findings
						Center of Tianjin				temperature, relative humidity		65years	the females and 0.19(95%CI:0.03-0.36) years for the males.<br>3. A 10μg/m³ increase in PM_{10}in lag01 was related to YLL increases of 0.02(95%CI:-0.09-0.14) years for the youngers and 0.27(95%CI:0.06-0.48) years for the elders.
Li et al 2018	Changping district in Beijing	2014-2017	Time-series study	——	$PM_{2.5}$	Environmental Protection Department of Changping district in Beijing	Cardiovascular disease	ICD-10: I00-I99	WHO life table	Long-term trends, day of the week, public holiday, relative humidity, air pressure	Lag1, lag7, lag14, lag0-21	1.Gender: female and male;<br>2.Age: <75years and ≥75years	1. A 10μg/m³ increase in $PM_{2.5}$in lag1 was related to YLL increases of 0.26 (95%CI:-0.04-0.56) years.<br>2. A 10μg/m³ increase in $PM_{2.5}$in lag1 was related to YLL increases of 0.08(95%CI:-0.10-0.26) years for the females and 0.18(95%CI:-0.07-0.42) years for the males.<br>3. A 10μg/m³ increase in $PM_{2.5}$in lag1 was related to YLL increases of 0.26(95%CI:-0.01-0.53) years for the youngers and 0.00(95%CI:-0.01-0.12) years for the elders.

Study	Location	Study period	Type of study	studied deaths size	Exposure	Source of exposure	Health outcome of YLL	Diagnosis standard	YLL calculate method	Adjustment factors	Lag paterns	Stratified analysis	Main findings
Zeng et al. 2019	6 urban districts in Tianjin	2001-2010	Time-series study	——	PM_{10}	From 6 monitoring sites from the Environmental Monitoring Center of Tianjin	Non-accidental disease, cardiovascular disease, respiratory disease	ICD-10: A00-R99, I00-I99, J00-J99,	WHO life table	Long-term trends, seasonality, day of the week, temperature, relative humidity	Lag01	——	1. A 10μg/m³ increase in PM_{10}in lag01 was related to YLL increases of5.83 (95%CI:4.13-7.54) years for the non-accidental diseases, 2.86 (95% CI:1.79 -3.93) years for the CVD, 0.84 (95%CI:0.45-1.23) years for the RD.

Table S3 The results before and after applying the trim and fill method

Diseases	Air pollutants	Effect estimates (95%CI) (Years)		Changes
		Before	After	
non-accidental diseases				
	$PM_{2.5}$	**1.99(1.00,2.97)**	**1.68(0.79,2.58)**	Significance unchanged, effect estimates decreased
	PM_{10}	**3.33(1.85,4.81)**	**3.33(1.85,4.81)**	No changes
	SO_2	**7.59(3.61,11.57)**	**7.59(3.61,11.57)**	No changes
	NO_2	**5.84(3.15,8.54)**	2.31(-0.58,5.19)	Changing from significant to non-significant
CVD				
	$PM_{2.5}$	0.28(-0.00,0.57)	0.28(-0.00,0.57)	No changes
	PM_{10}	**1.05(0.17,1.94)**	**1.05(0.17,1.94)**	No changes
	SO_2	3.71(-0.37,7.80)	0.56(-2.79,3.91)	Non-significance unchanged, effect estimates decreased
	NO_2	**3.22(1.16,5.27)**	**2.98(1.24,4.71)**	Significance unchanged, effect estimates decreased
RD				

$PM_{2.5}$	0.32(-0.19,0.82)	0.32(-0.19,0.82)	No changes
PM_{10}	**0.44(0.06,0.82)**	0.26(-0.16,0.67)	Changing from significant to non-significant
SO_2	1.37(-0.45,3.19)	1.37(-.45,3.19)	No changes
NO_2	0.96(-0.03,1.96)	0.96(-0.03,1.96)	No changes

Results expressed in changes in disease-specific YLL per 10μg/m³ increase in air pollutants with 95% confidence intervals. $PM_{2.5}$: particle with aerodynamic diameter ≤ 2.5μm; PM_{10}: particle with aerodynamic diameter ≤ 2.5μm; SO_2: sulfur dioxide; NO_2: nitrogen dioxide; CVD: cardiovascular disease;RD: respiratory disease.Effect estimates **bold** were statistically significant.

Part B Risk of bias assessment for each study

# 1 Guo et al. 2013		
**Design**	Time-series study	
**Participants**	Deaths in 8 urban districts in Beijing , 2004-2008	
**Exposure**	$PM_{2.5}$, PM_{10}, SO_2, NO_2	
**Outcomes**	Non-accidental disease	
**Risk of Bias Assessment**		
**Bias Domain**	**Criterion supportive response**	**Judgment**
Exposure Assessment	-$PM_{2.5}$ was monitored at the main campus of Peking University, located in the urban center. PM_{10}, $SO_2$and NO_2 concentrations were obtained from the Beijing municipal environmental monitoring center, which had eight fixed monitoring sites distributed in different part of the urban area.<br>- For $PM_{2.5}$, daily average concentration was computed. The daily mean concentrations of PM_{10}, $SO_2$and NO_2 were calculated by averaging daily data over all monitoring sites.	Probably low
Outcome Assessment	Mortality data for all ages on non-accidental causes were obtained from the death classification system at the Beijing Public Security Bureau.Mortality cause classified standards were not mentioned.	Probably high
Confounding	Adjusted for long-term trends, seasonality, temperature, relative humidity, air pressure, and the day of the week.	Low
Selection	Study included all deaths that were registered residents of urban areas of Beijing city and occurred between 1 January 2004 and 31 December 2008.	Low
Incomplete outcome data	Daily YLL were calculated by summing the YLL for all deaths on that day. However, any potential errors or missing data did not depend on air pollution levels.	Low
Selective outcome reporting	All pre-specified outcomes are reported	Low
Conflict of interest	No potential conflicts of interest were declared.	Low
Other sources of	No other potential sources of bias identified.	Low

# 2Lu et al. 2015	
**Design**	Time-series study
**Participants**	Deaths in 9 urban districts in Nanjing, 2009-2013
**Exposure**	PM_{10}, SO_2, NO_2
**Outcomes**	Non-accidental disease

Risk of Bias Assessment

**Bias Domain**	**Criterion supportive response**	**Judgment**
Exposure Assessment	-The daily data on PM_{10}, SO_2 and NO_2 for 2009-2013 was collected from the Nanjing Environmental Monitoring center.<br>-Daily concentrations were provided as daily mean values measured from 9 stat-controlled monitoring stations.<br>-No information about the missing data of daily average	Probably low
Outcome Assessment	Daily mortality data (excluding accidental deaths) were obtained from the Jiangsu Provincial Center for Disease Control and Prevention. Causes of death were coded according to the ICD-10 (A00-R99) for all ages.	Low
Confounding	Adjusted for long-term trends, seasonality, temperature, relative humidity, barometric pressure, the day of the week and public holidays.	Low
Selection	Study included all non-accidental deaths for all ages thatoccurred between 1 January 2009 and 31 December 2013.	Low
Incomplete outcome data	Daily YLL were calculated by summing the YLL for all deaths on that day. However, any potential errors or missing data did not depend on air pollution levels.	Low
Selective outcome reporting	All pre-specified outcomes are reported	Low
Conflict of interest	No potential conflicts of interest were declared.	Low
Other sources of	No other potential sources of bias identified.	Low

# 3He et al. 2016		
**Design**	Time-series study	
**Participants**	Deaths in Ningbo, 2009-2013	
**Exposure**	$PM_{2.5}$, PM_{10}, SO_2, NO_2	
**Outcomes**	Non-accidental disease	
**Risk of Bias Assessment**		
**Bias Domain**	**Criterion supportive response**	**Judgment**
Exposure Assessment	-The concentrations of $PM_{2.5}$, PM_{10}, SO_2 and NO_2 were monitored consecutively at 11 monitoring sites that cover all districts and counties of Ningbo, including both urban and suburban areas.<br>- The concentrations of $PM_{2.5}$, PM_{10}, SO_2 and NO_2 were from the Environment Monitoring Center of Ningbo. Data for $PM_{2.5}$ were available from 2011-2013, while other pollutants were from 2009-2013.<br>-Daily average concentrations of pollutants for individual monitoring sites and then the average levels for the whole Ningbo city were derived.	Probably low
Outcome Assessment	Data on non-accidental deaths were obtained from the local mortality register based in the Ningbo Municipal Center for Disease Prevention and Control. Causes of	Low
Confounding	Adjusted for long-term trends, seasonality, temperature, relative humidity, wind speed, and the day of the week.	Low
Selection	Study included all non-accidental deaths for the registered residents for all ages thatoccurred between 1 January 2009 and 31 December 2013.	Low
Incomplete outcome data	Daily YLL were calculated by summing the YLL for all deaths on that day. However, any potential errors or missing data did not depend on air pollution levels.	Low
Selective outcome reporting	All pre-specified outcomes are reported	Low
Conflict of interest	No potential conflicts of interest were declared.	Low

Other sources of bias	No other potential sources of bias identified.	Low

# 4Yang et al. 2016	
**Design**	Time-series study
**Participants**	Deaths in 6 central urban districts in Guangzhou, 2004-2007
**Exposure**	PM_{10}, SO_2, NO_2
**Outcomes**	Non-accidental disease, cardiovascular disease, ischemic heart disease, stroke

Risk of Bias Assessment

**Bias Domain**	**Criterion supportive response**	**Judgment**
Exposure Assessment	-Daily data of PM_{10}, SO_2 and NO_2 from 2004-2007 were supplied by Guangzhou Bureau of Environmental Protection.<br>-No information about the missing data of daily average	Probably low
Outcome Assessment	Individual data of deaths were provided by Guangzhou Bureau of Health from 1 January 2004 to 31 December 2007 in six central urban districts of Guangzhou. Causes of death were coded according to the ICD-10 (non-accidental mortality: A00-R99; cardiovascular disease: I00-I99;	Low
Confounding	Adjusted for long-term trends, seasonality, temperature, relative humidity, atmospheric pressure, the day of the week [illegible]	Low
Selection	Study included all deaths from 1 January 2004 to 31 December 2007 in six central urban districts of Guangzhou.	Low
Incomplete outcome data	Daily YLL were calculated by summing the YLL for all deaths on that day. However, any potential errors or missing data did not depend on air pollution levels.	Low
Selective outcome reporting	All pre-specified outcomes are reported	Low
Conflict of interest	No potential conflicts of interest were declared.	Low
Other sources of	No other potential sources of bias identified.	Low

# 5Chen et al. 2017	
**Design**	Time-series study
**Participants**	Deaths in 19 districts in Chengdu, 2010-2014
**Exposure**	PM_{10}
**Outcomes**	Non-accidental disease, respiratory disease, cardiovascular disease

Risk of Bias Assessment

**Bias Domain**	**Criterion supportive response**	**Judgment**
Exposure Assessment	-Daily air pollutant data over the period were obtained from the Sichuan Environmental Monitoring Center.<br>-The daily average concentration of PM_{10} was obtained by averaging the daily mean values from the 12 monitoring stations.<br>-No information about the missing data of daily average	Probably low
Outcome Assessment	Data on daily mortality were obtained from 19 death surveillance sites of the Sichuan Center for Disease Control and Prevention which are located in all the 19 districts. Causes of death were coded according to the ICD-10 (non-accidental disease: A00-R99; respiratory disease:	Low
Confounding	Adjusted for long-term trends, the seasonal trend, temperature, relative humidity, air pressure, wind speed, and	Low
Selection	Study included all daily mortality data for all ages thatoccurred between 1 January 2010 and 31 December	Low
Incomplete outcome data	Daily YLL were calculated for each death. However, any potential errors or missing data did not depend on air	Low
Selective outcome reporting	All pre-specified outcomes are reported	Low
Conflict of interest	No potential conflicts of interest were declared.	Low
Other sources of	No other potential sources of bias identified.	Low

# 6Li et al. 2017	
**Design**	Time-series study
**Participants**	Deaths of chronic obstructive pulmonary disease in Ningbo, 2011-2015
**Exposure**	$PM_{2.5}$, PM_{10}
**Outcomes**	Chronic obstructive pulmonary disease

Risk of Bias Assessment

**Bias Domain**	**Criterion supportive response**	**Judgment**
Exposure Assessment	-Concentrations of $PM_{2.5}$, PM_{10} were monitored continuously at 11 monitoring sites which cover urban and suburban areas of Ningbo.<br>-The Environment Monitoring Center of Ningbo collects the pollutants concentrations from all the monitoring sites of the city and records the hourly concentration, from which the daily average levels for all the individual monitoring sites and the whole city levels were derived.<br>-Number of missing is less than 1% and the missing values were substituted with the mean daily value.	Low
Outcome Assessment	Daily mortality data on deaths of COPD between January 1st 2011 and December 31th 2015 were obtained from Ningbo Municipal Center for Disease Control and Prevention. COPD deaths were identified according to the ICD-10:	Low
Confounding	Adjusted for long-term trends, seasonality, temperature, relative humidity, and the day of the week.	Low
Selection	Study included all COPD deaths for all ages and all registered residents thatoccurred between 1 January 2011 and 31 December 2015.	Low
Incomplete outcome data	Daily YLL were calculated by summing the YLL for all deaths on the same day. However, any potential errors or missing data did not depend on air pollution levels.	Low
Selective outcome reporting	All pre-specified outcomes are reported	Low
Conflict of interest	No potential conflicts of interest were declared.	Low
Other sources of	No other potential sources of bias identified.	Low

# 7Ni et al. 2017a	
**Design**	Time-series study
**Participants**	Deaths in 6 urban districts in Tianjin, 2001-2010
**Exposure**	NO_2
**Outcomes**	Non-accidental disease

Risk of Bias Assessment

**Bias Domain**	**Criterion supportive response**	**Judgment**
Exposure Assessment	-The daily data on NO_2 for 2001-2010 was collected from the Tianjin Environmental Monitoring center.<br>-Daily concentrations were provided as daily mean values from 6 stat-controlled monitoring stations.<br>-No information about the missing data of daily average concentration was reported.	Probably low
Outcome Assessment	Daily mortality data were obtained from the Tianjin Center for Disease Control and Prevention. Causes of death were coded according to the ICD-10 (A00-R99).	Low
Confounding	Adjusted for long-term trends, seasonality, temperature, relative humidity, and the day of the week.	Low
Selection	Study included all non-accidental deaths thatoccurred between 1 January 2001 and 31 December 2010.	Low
Incomplete outcome data	Daily YLL were calculated by summing the YLL for all deaths on that day. However, any potential errors or missing data did not depend on air pollution levels.	Low
Selective outcome reporting	All pre-specified outcomes are reported	Low
Conflict of interest	No potential conflicts of interest were declared.	Low
Other sources of	No other potential sources of bias identified.	Low

# 8Ni et al. 2017b	
**Design**	Time-series study
**Participants**	Deaths in Nankai districts in Tianjin, 2001-2010
**Exposure**	SO_2
**Outcomes**	Non-accidental disease, cardiovascular disease, respiratory disease

Risk of Bias Assessment

**Bias Domain**	**Criterion supportive response**	**Judgment**
Exposure Assessment	-The daily data on SO_2 for 2001-2010 was collected from the Tianjin Environmental Monitoring center (n=3652).<br>-Daily concentrations were provided as daily mean values from the monitoring station located in Nankai district in Tianjin.<br>-No information about the missing data of daily average	Probably low
Outcome Assessment	Daily mortality data were obtained from the Tianjin Center for Disease Control and Prevention. Causes of death were coded according to the ICD-10 (non-accidental disease: A00-R99; cardiovascular disease: I00-I99; respiratory	Low
Confounding	Adjusted for long-term trends, seasonality, temperature, relative humidity, and the day of the week.	Low
Selection	Study included all deaths thatoccurred between 1 January 2001 and 31 December 2010.	Low
Incomplete outcome data	Daily YLL were calculated by summing the YLL for all deaths on that day. However, any potential errors or missing data did not depend on air pollution levels.	Low
Selective outcome reporting	All pre-specified outcomes are reported	Low
Conflict of interest	No potential conflicts of interest were declared.	Low
Other sources of	No other potential sources of bias identified.	Low

# 9Zeng et al. 2017a	
**Design**	Time-series study
**Participants**	Deaths in 6 urban districts in Tianjin, 2001-2010
**Exposure**	PM_{10}
**Outcomes**	Non-accidental disease

Risk of Bias Assessment

**Bias Domain**	**Criterion supportive response**	**Judgment**
Exposure Assessment	-During the study period, the daily average concentration of PM_{10} from the city central air-monitoring stations of each district was obtained from the Tianjin Environmental Monitoring Center.<br>-Each district has one central air-monitoring station.<br>-There was less than 0.5% missing data of PM_{10}. The	Low
Outcome Assessment	Daily mortality data on non-accidental death between January 1st 2001 and December 31st 2010 was obtained from the Tianjin Center for Disease Control and Prevention. The causes of non-accidental death were classified and coded based on ICD10 (A00-R99).	Low
Confounding	Adjusted for long-term trends, seasonality, temperature, relative humidity, and the day of the week.	Low
Selection	Daily mortality data on non-accidental death between January 1st 2001 and December 31st 2010 was obtained from the Tianjin Center for Disease Control and Prevention.	Low
Incomplete outcome data	Daily YLL were calculated by summing the YLL for all deaths on that day. However, any potential errors or missing data did not depend on air pollution levels.	Low
Selective outcome reporting	All pre-specified outcomes are reported	Low
Conflict of interest	The authors declare no conflicts of interest.	Low
Other sources of	No other potential sources of bias identified.	Low

# 10Zeng et al. 2017b	
**Design**	Time-series study
**Participants**	Deaths in 6 urban districts in Tianjin, 2001-2010
**Exposure**	PM_{10}
**Outcomes**	Respiratory disease

Risk of Bias Assessment

**Bias Domain**	**Criterion supportive response**	**Judgment**
Exposure Assessment	-The daily air pollutant monitoring data was obtained from the Tianjin Environmental Monitoring Center.<br>-The air pollutant monitoring data was collected from six urban districts in Tianjin.<br>-No information about the missing data of daily average concentration was reported.	Probably low
Outcome Assessment	Daily mortality data regarding respiratory death (ICD-10: A00-R99) was obtained from the Death Register and Report Information System, Tianjin Center for Disease Control and	Low
Confounding	Adjusted for long-term trends, seasonality, temperature, relative humidity, and the day of the week.	Low
Selection	Study collected mortality data from six urban districts in Tianjin from 1 January 2001 and 31 December 2010. Daily mortality data regarding respiratory death (ICD-10: A00-R99) was obtained from the Death Register and Report Information System, Tianjin Center for Disease Control and Prevention.	Low
Incomplete outcome data	Daily YLL were calculated by summing the YLL for all deaths on the same day. However, any potential errors or missing data did not depend on air pollution levels.	Low
Selective outcome reporting	All pre-specified outcomes are reported	Low
Conflict of interest	The authors declare no conflicts of interest.	Low
Other sources of	No other potential sources of bias identified.	Low

# 11Zhang et al. 2017	
**Design**	Time-series study
**Participants**	Deaths in 2 urban districts in Wuhan, 2009-2012
**Exposure**	PM_{10}, SO_2, NO_2
**Outcomes**	Non-accidental disease, cardiovascular disease, respiratory disease, stroke, [illegible]

Risk of Bias Assessment

**Bias Domain**	**Criterion supportive response**	**Judgment**
Exposure Assessment	-The daily concentration of PM_{10} during 2009-2012 was collected from the Wuhan Environmental Monitoring Center.<br>-Daily concentrations were collected from two urban districts.<br>-No information about the missing data of daily average	Probably low
Outcome Assessment	Daily mortality data from January 1, 2009 through December 31, 2012 were obtained from the Hubei Provincial Center for Disease Control and Prevention. Causes of death were coded according to the ICD-10. Daily non-accidental death cases (ICD-10: A00-R99) were included in databases, and were divided into several mortality categories: cardiovascular (I00-I99), respiratory	Low
Confounding	Adjusted for long-term trends, seasonality, temperature, relative humidity, barometric pressure, the day of the week	Low
Selection	Daily mortality data from January 1, 2009 through December 31, 2012 were obtained from the Hubei Provincial Center for Disease Control and Prevention. Daily non-accidental death cases (ICD-10: A00-R99) were	Low
Incomplete outcome data	Daily YLLs could be consequently obtained by summing the YLL of all death cases on that day. However, any potential errors or missing data did not depend on air	Low
Selective outcome reporting	All pre-specified outcomes are reported	Low
Conflict of interest	The authors declare they have no competing financial	Low
Other sources of	No other potential sources of bias identified.	Low

# 12Zhu et al. 2017	
**Design**	Time-series study
**Participants**	Deaths in Wuxi, 2012-2015
**Exposure**	$PM_{2.5}$, PM_{10}, SO_2, NO_2
**Outcomes**	Non-accidental disease, cardiovascular disease, respiratory disease, diabetes

Risk of Bias Assessment

**Bias Domain**	**Criterion supportive response**	**Judgment**
Exposure Assessment	-In this study, the data of air pollutants were simultaneously collected from seven state-controlled monitoring stations distributed in different administrative districts of Wuxi from 2012-2015.<br>-The data from monitoring sites were obtained from the Wuxi Municipal Environmental Monitoring Center and averaged to calculate the daily 24-h mean concentration of each pollutant.<br>-No information about the missing data of daily average	Probably low
Outcome Assessment	Daily mortality data from 1 January 2012 to 31 December 2015 were collected from the database of the Wuxi Center for Disease Control and Prevention.The data in this study were coded according to ICD-10 and further classified into deaths because of non-accidental causes (A00-A99), cardiovascular diseases (I00-I99), respiratory diseases (J00-J98) and diabetes	Low
Confounding	Adjusted for time, temperature, relative humidity, and the day	Low
Selection	Daily mortality data from 1 January 2012 to 31 December 2015 were collected from the database of the Wuxi Center for Disease Control and Prevention.	Low
Incomplete outcome data	YLL values were calculated for each individual death by matching the age and sex to the life tables and were then summed to yield the YLL corresponding to each day. However, any potential errors or missing data did not depend	Low
Selective outcome reporting	All pre-specified outcomes are reported	Low
Conflict of interest	No potential conflicts of interest were declared.	Low
Other sources of	No other potential sources of bias identified.	Low

# 13Huang et al. 2018a	
**Design**	Time-series study
**Participants**	Deaths of ischemic heart deaths in Ningbo, 2011-2015
**Exposure**	$PM_{2.5}$, SO_2, NO_2
**Outcomes**	Ischemic heart disease

Risk of Bias Assessment

**Bias Domain**	**Criterion supportive response**	**Judgment**
Exposure Assessment	-The daily concentrations of air pollutants were collected from the Environment Monitoring Center of Ningbo between Jan 1st 2011 and December 31st 2015.<br>-The air pollutants were monitored consecutively at eight monitoring sites, which cover eleven districts and counties in Ningbo.<br>-No information about the missing data of daily average	Probably low
Outcome Assessment	Daily IHD mortality data from 2011 to 2015 were obtained from Ningbo Municipal Center for Disease Control and Prevention. ICD-10: I20-I25 was used as the identifying	Low
Confounding	Adjusted for long-term trends, temperature, relative humidity, the day of the week. Seasonality was not included.	Probably low
Selection	Study included all IHD deaths thatoccurred from 2011 to	Low
Incomplete outcome data	Daily YLL were calculated by summing the YLL for all the individuals who died from IHD on the same day.However, any potential errors or missing data did not depend on air	Low
Selective outcome reporting	All pre-specified outcomes are reported	Low
Conflict of interest	The authors declare no conflicts of interest.	Low
Other sources of	No other potential sources of bias identified.	Low

# 14Huang et al. 2018b	
**Design**	Time-series study
**Participants**	Deaths of chronic obstructive pulmonary disease in Ningbo, 2011-2015
**Exposure**	O_3
**Outcomes**	Chronic obstructive pulmonary disease

Risk of Bias Assessment

Bias Domain	Criterion supportive response	Judgment
Exposure Assessment	-The daily air pollution data from Jan 1st 2011 to Dec 31st 2015 were obtained from the Environment Monitoring Center of Ningbo.<br>-There are 8 monitoring sites that cover all districts and counties in Ningbo.<br>-The daily concentration of each air pollutant was calculated [illegible]	Probably low
Outcome Assessment	Daily COPD mortality data during the same period were collected from the Ningbo Municipal Center for Disease Control and Prevention. COPD deaths were identified	Low
Confounding	Adjusted for long-term trends, seasonality, temperature, relative humidity, and the day of the week.	Low
Selection	Daily COPD mortality data during the same period were collected from the Ningbo Municipal Center for Disease Control and Prevention.Daily death count was defined as the number of deaths occurring on a single day, and the data was	Low
Incomplete outcome data	The daily YLL was calculated. However, any potential errors or missing data did not depend on air pollution levels.	Low
Selective outcome reporting	All pre-specified outcomes are reported	Low
Conflict of interest	The authors declared that they have no competing interests.	Low
Other sources of	No other potential sources of bias identified.	Low

**# 15Liang et al. 2018**	
**Design**	Time-series study
**Participants**	Deaths of acute myocardial infraction in Hong Kong, 2011-2015
**Exposure**	$PM_{2.5}$, SO_2, NO_2, O_3
**Outcomes**	Acute myocardial infraction disease

Risk of Bias Assessment

**Bias Domain**	**Criterion supportive response**	**Judgment**
Exposure Assessment	-Hourly monitoring data for $PM_{2.5}$, SO_2, NO_2, O_3 in 14 monitoring stations were collected by the Hong Kong Environmental Protection Department from 2011 to 2015. 10 general stations data was used to represent the general population exposure on a regular basis, after excluding three roadside stations and one general station on a remote island.<br>-The 24 hours mean concentrations were calculated for each station first and then average over ten stations were calculated to represent the daily exposure levels of the whole population.<br>-No information about the missing data of daily average	Probably low
Outcome Assessment	Daily data on mortality due to AMI in Hong Kong from January 1, 2011 to December 31, 2015 were obtained from Hong Kong Census and Statistics Department. The death cause was coded according to ICD-10. In the study, the daily mortality count from AMI (I121) was abstracted.	Low
Confounding	Adjusted for long-term trends, seasonality, temperature, relative humidity, the day of the week and public holidays.	Low
Selection	Study included all AMI deaths in Hong Kong from January 1, 2011 to December 31, 2015.	Low
Incomplete outcome data	Daily total YLL were calculated as the sum of YLL of all deaths due to AMI on the same day. However, any potential errors or missing data did not depend on air pollution levels.	Low
Selective outcome reporting	All pre-specified outcomes are reported	Low
Conflict of interest	No potential conflicts of interest were declared.	Low
Other sources of	No other potential sources of bias identified.	Low

**# 16Zeng et al. 2018a**	
**Design**	Time-series study
**Participants**	Deaths of cardiovascular disease in Tianjin, 2001-2010
**Exposure**	PM_{10}
**Outcomes**	Cardiovascular disease, ischemic heart disease, stroke

Risk of Bias Assessment

**Bias Domain**	**Criterion supportive response**	**Judgment**
Exposure Assessment	-The data on daily mean concentrations of PM_{10} from January 1, 2001 to December 31, 2010 was collected from the Tianjin Environmental Monitoring center.<br>-No information about the missing data of daily average concentration was reported.	Probably low
Outcome Assessment	Daily mortality data were obtained from the Tianjin Center for Disease Control and Prevention. Causes of death were coded according to the ICD-10 (cardiovascular disease: I00-I99, ischemic heart disease: I60-I69, stroke: I20-I25).	Low
Confounding	Adjusted for time, temperature, relative humidity and the	Low
Selection	Study included all cardiovascular disease deaths thatoccurred between 1 January 2001 and 31 December [illegible]	Low
Incomplete outcome data	Daily YLL were calculated by summing the YLL for all deaths on that day. However, any potential errors or missing data did not depend on air pollution levels.	Low
Selective outcome reporting	All pre-specified outcomes are reported	Low
Conflict of interest	No potential conflicts of interest were declared.	Low
Other sources of	No other potential sources of bias identified.	Low

# 17Zeng et al. 2018b	
**Design**	Time-series study
**Participants**	Deaths of chronic obstructive pulmonary disease in 6 urban districts in Tianjin, 2001-2010
**Exposure**	PM_{10}
**Outcomes**	Chronic obstructive pulmonary disease

Risk of Bias Assessment

**Bias Domain**	**Criterion supportive response**	**Judgment**
Exposure Assessment	-The daily air pollutant monitoring data were obtained from Tianjin Environmental Monitoring center.<br>-There were six monitoring stations in urban areas, as for the calculation of daily average concentrations of air pollutants; at least 75% of the 1-h values must be available on that particular day.<br>-The percentage of missing data regarding air pollutants was less than 0.5%.	Low
Outcome Assessment	The daily mortality data on COPD deaths were obtained from the Death Register and Report Information System, Tianjin Centers for Disease Control and Prevention. Based on the ICD-10, COPD was coded J41-J44.	Low
Confounding	Adjusted for long-term trends, seasonality, temperature, relative humidity and the day of the week.	Low
Selection	Study included all COPD deaths thatoccurred between 1 January 2001 and 31 December 2010.	Low
Incomplete outcome data	Daily YLL were calculated by summing the YLL for all deaths on that day. However, any potential errors or missing data did not depend on air pollution levels.	Low
Selective outcome reporting	All pre-specified outcomes are reported	Low
Conflict of interest	No potential conflicts of interest were declared.	Low
Other sources of	No other potential sources of bias identified.	Low

# 18Li et al. 2019	
**Design**	Time-series study
**Participants**	Deaths of cardiovascular disease in Changping district in Beijing, 2014-2017
**Exposure**	$PM_{2.5}$
**Outcomes**	Cardiovascular disease

Risk of Bias Assessment

**Bias Domain**	**Criterion supportive response**	**Judgment**
Exposure Assessment	-The daily data on $PM_{2.5}$ for 2014-2017 was provided by the Environmental Protection Department of Changping district in Beijing<br>-No information about the missing data of daily average	Probably low
Outcome Assessment	Daily mortality data were collected by researcher. Causes of death were coded according to the ICD-10 (I00-I99).	Probably low
Confounding	Adjusted for long-term trends, temperature, relative humidity, air pressure, the day of the week and public holidays. [illegible]	Probably low
Selection	Study included all cardiovascular disease deaths thatoccurred between 2014 and 2017.	Low
Incomplete outcome data	Daily YLL were calculated by summing the YLL for all deaths on that day. However, any potential errors or missing data did not depend on air pollution levels.	Low
Selective outcome reporting	All pre-specified outcomes are reported	Low
Conflict of interest	No potential conflicts of interest were declared.	Low
Other sources of	No other potential sources of bias identified.	Low

# 19Zeng et al. 2019		
**Design**	Time-series study	
**Participants**	Deaths in 6 urban districts in Tianjin, 2001-2010	
**Exposure**	PM_{10}	
**Outcomes**	Non-accidental disease, cardiovascular disease, respiratory disease	
**Risk of Bias Assessment**		
**Bias Domain**	**Criterion supportive response**	**Judgment**
Exposure Assessment	-The daily concentrations of PM_{10} between January 1, 2001 and December 31, 2010 were obtained from the Tianjin Environmental Monitoring center.<br>-The daily concentrations were calculated as the daily average values of the monitoring data from the city central air-monitoring sites of each district, each district has one central air-monitoring site.<br>-The percentage of missing data regarding air pollutants was	Low
Outcome Assessment	The death monitoring data during 2001-2010 was collected from Tianjin Centers for Disease Control and Prevention. The causes of deaths were classified and coded according to the ICD-10, as non-accidental disease (A00-A99), cardiovascular disease (I00-I99), respiratory disease	Low
Confounding	Adjusted for long-term trends, seasonality, temperature, relative humidity, and the day of the week.	Low
Selection	Study included all non-accidental deaths thatoccurred between 1 January 2001 and 31 December 2010.	Low
Incomplete outcome data	Daily YLL were calculated by summing the YLL for all deaths on that day. However, any potential errors or missing data did not depend on air pollution levels.	Low
Selective outcome reporting	All pre-specified outcomes are reported	Low
Conflict of interest	The authors declare that they have no conflict of interest.	Low
Other sources of	No other potential sources of bias identified.	Low

	Exposure					Health outcomes of YLL								Health outcome classified standard		Quality of exposure measurements		Adjustments factors						Quality assessment results	
	$PM_{2.5}$	PM_{10}	SO_2	NO_2	O_3	Non-accidental disease	RD	CVD	COPD	IHD	Stroke	AMI	Diabetes mellitus	ICD10	Others	daily with less than 25% missing data	daily with more than 25% missing data	Temperature	Relative humidity	Long-term trends	seasonality	Day of week	Other factors	High quality	Low quality
Guo et al. 2013	■	■	■	■		■									■	■		■	■	■	■	■	■		■
Lu et al. 2015		■	■	■		■								■		■		■	■	■	■	■	■	■	
He et al. 2016	■	■	■	■		■								■		■		■	■	■	■	■	■	■	
Yang et al. 2016		■	■	■		■		■		■	■			■		■		■	■	■	■	■	■	■	
Chen et al. 2017		■				■	■	■						■		■		■	■	■	■	■	■	■	
Li et al. 2017	■	■							■					■		■		■	■	■	■	■		■	
Ni et al. 2017a				■		■								■		■		■	■	■	■	■		■	
Ni et al. 2017b			■			■	■	■						■		■		■	■	■	■	■		■	
Zeng et al. 2017a		■				■								■		■		■	■	■	■	■		■	
Zeng et al. 2017b		■					■							■		■		■	■	■	■	■		■	
Zhang et al. 2017		■				■	■	■		■	■			■		■		■	■	■	■	■	■	■	
Zhu et al. 2017	■	■	■	■		■	■	■					■	■		■		■	■	■	■	■		■	
Huang et al. 2018a	■		■	■						■				■		■		■	■	■		■			■
Huang et a. 2018b	■				■				■					■		■		■	■	■	■	■		■	
Liang et al. 2018	■		■	■	■							■		■		■		■	■	■	■	■	■	■	
Zeng et al. 2018a		■						■		■	■			■		■		■	■	■	■	■		■	
Zeng et al. 2018b		■							■					■		■		■	■	■	■	■		■	
Li et al 2019	■							■						■		■			■	■		■	■		■
Zeng et al. 2019		■				■	■	■						■		■		■	■	■	■	■		■	

Figure S1 Characteristicsof the included studies according to quality assessments, exposure assessment, health outcomes of YLL, adjustment factors, and other characteristics. Colored grid indicated that these results were expressed by the study. High quality: the study was high quality (score=5) according to the methodological quality assessments; Low quality: the study was low quality (score<5) according to the methodological quality assessments. PM2.5: particle with aerodynamic diameter ≤ 2.5μm; PM10: particle with aerodynamic diameter ≤ 2.5μm; SO2: sulfur dioxide; NO2:

nitrogen dioxide; O3: ozone; RD: respiratory disease; CVD: cardiovascular disease; COPD: chronic obstructive pulmonary disease; AMI: acutemyocardialinfarction; IHD: Ischemic Heart Disease; ICD: International Classification of Disease.

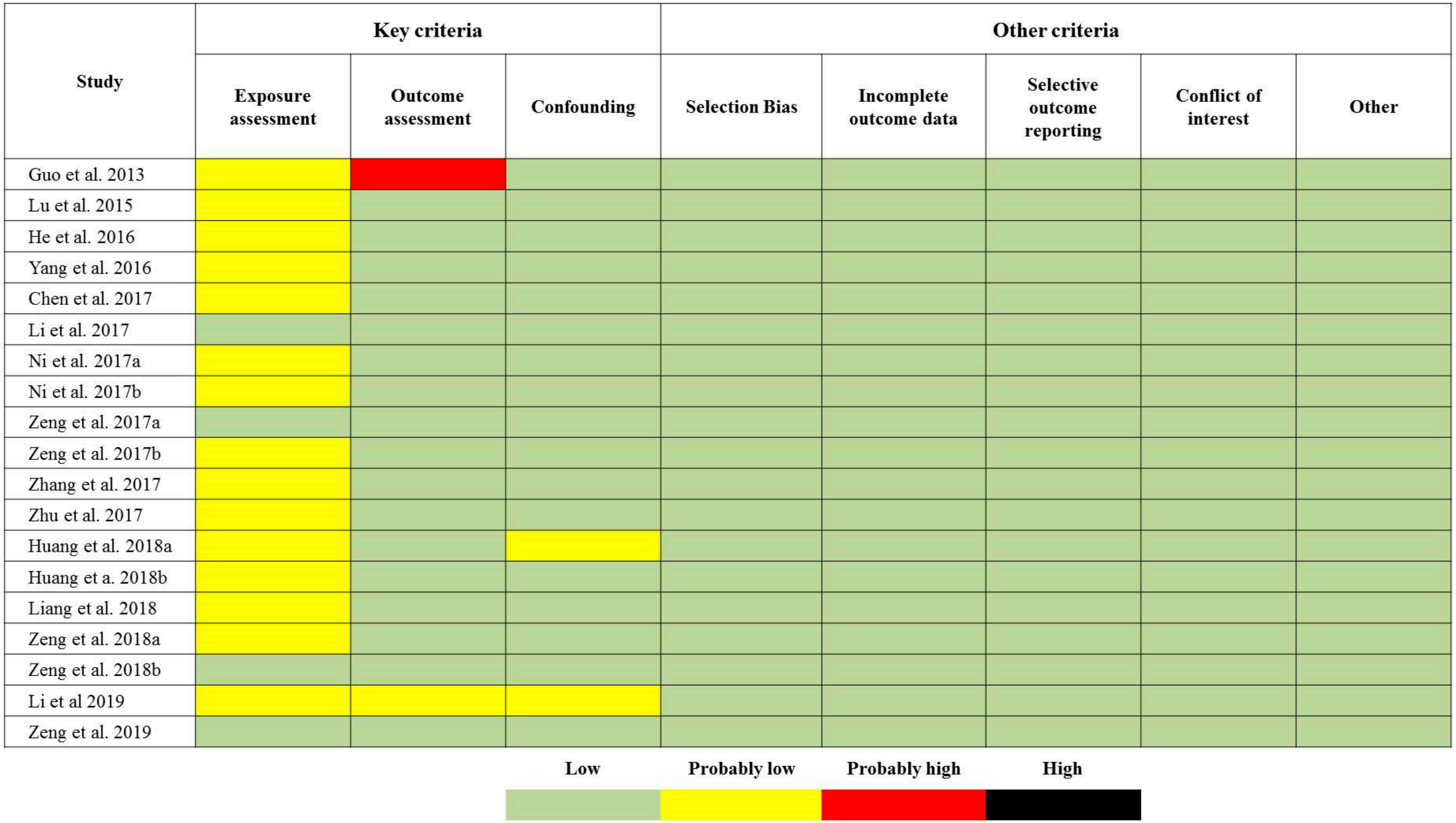

Figure S2 Risk of bias assessment for each study

A

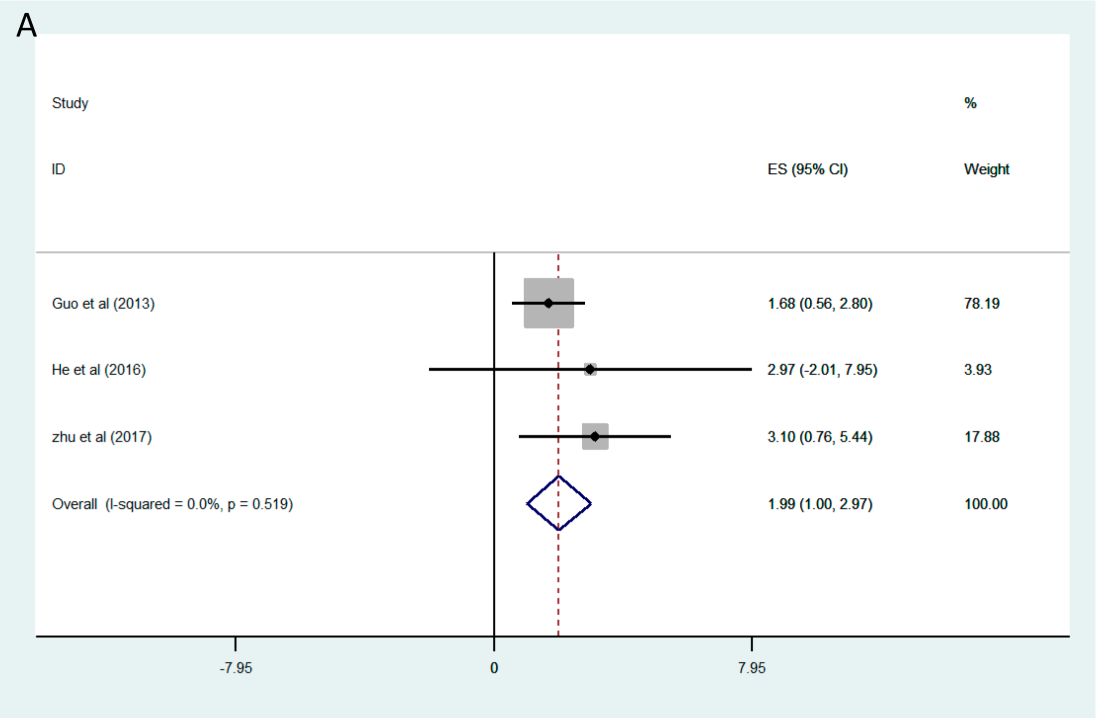
Study
ID
ES (95% CI)
%
Weight
Guo et al (2013)
1.68 (0.56, 2.80)
78.19
He et al (2016)
2.97 (-2.01, 7.95)
3.93
zhu et al (2017)
3.10 (0.76, 5.44)
17.88
Overall (I-squared = 0.0%, p = 0.519)
1.99 (1.00, 2.97)
100.00
-7.95
0
7.95

B

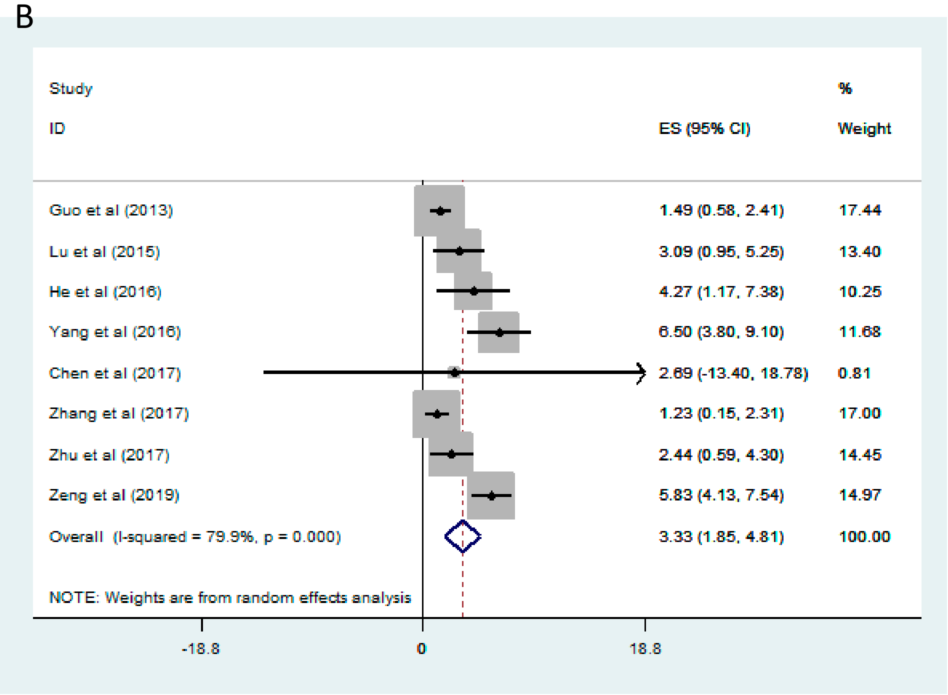
Study
ID
ES (95% CI)
%
Weight
Guo et al (2013)
1.49 (0.58, 2.41)
17.44
Lu et al (2015)
3.09 (0.95, 5.25)
13.40
He et al (2016)
4.27 (1.17, 7.38)
10.25
Yang et al (2016)
6.50 (3.80, 9.10)
11.68
Chen et al (2017)
2.69 (-13.40, 18.78)
0.81
Zhang et al (2017)
1.23 (0.15, 2.31)
17.00
Zhu et al (2017)
2.44 (0.59, 4.30)
14.45
Zeng et al (2019)
5.83 (4.13, 7.54)
14.97
Overall (I-squared = 79.9%, p = 0.000)
3.33 (1.85, 4.81)
100.00
NOTE: Weights are from random effects analysis
-18.8
0
18.8

C

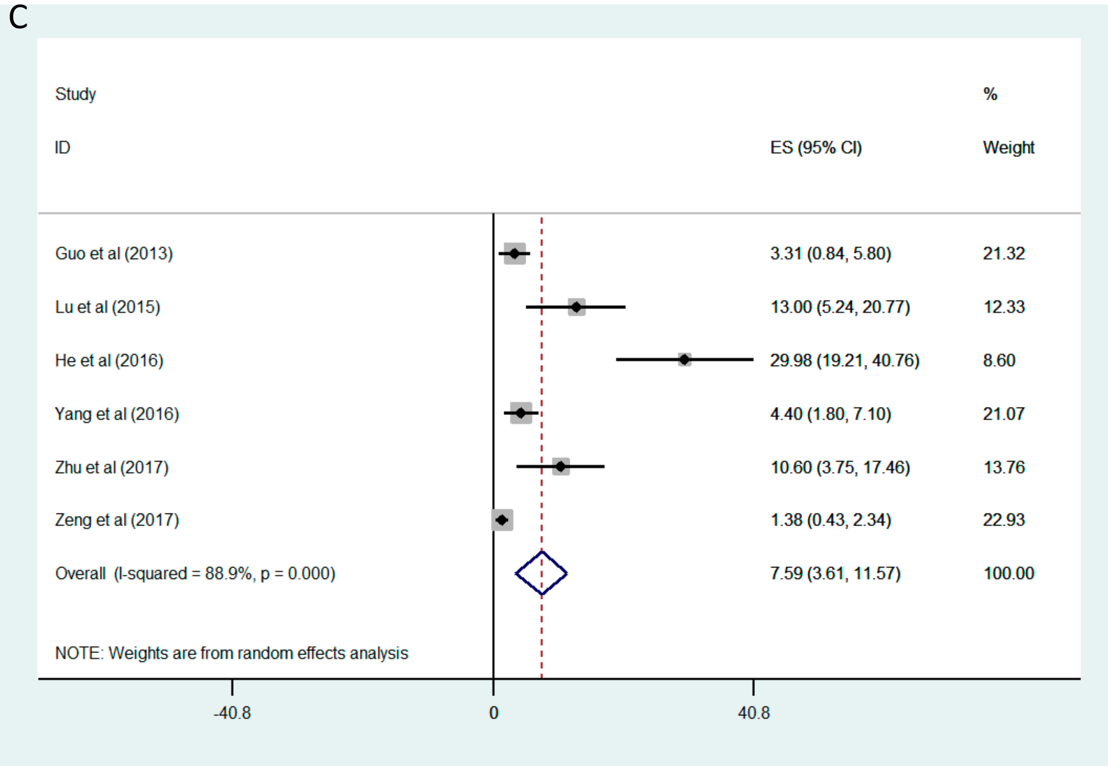

D

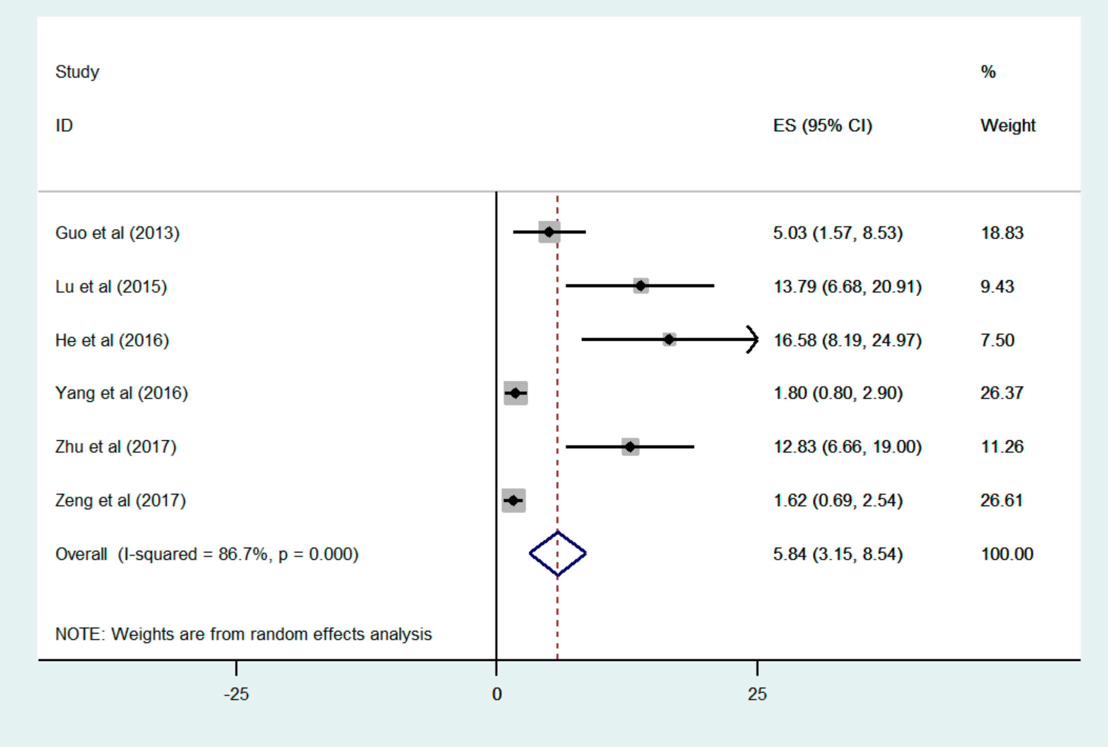

Figure S3 Forest plot and pooled effect estimates of the ambient air pollution on the YLL of non-accidental disease. A. $PM_{2.5}$; B. PM_{10}; C. SO_2; D. NO_2.

A

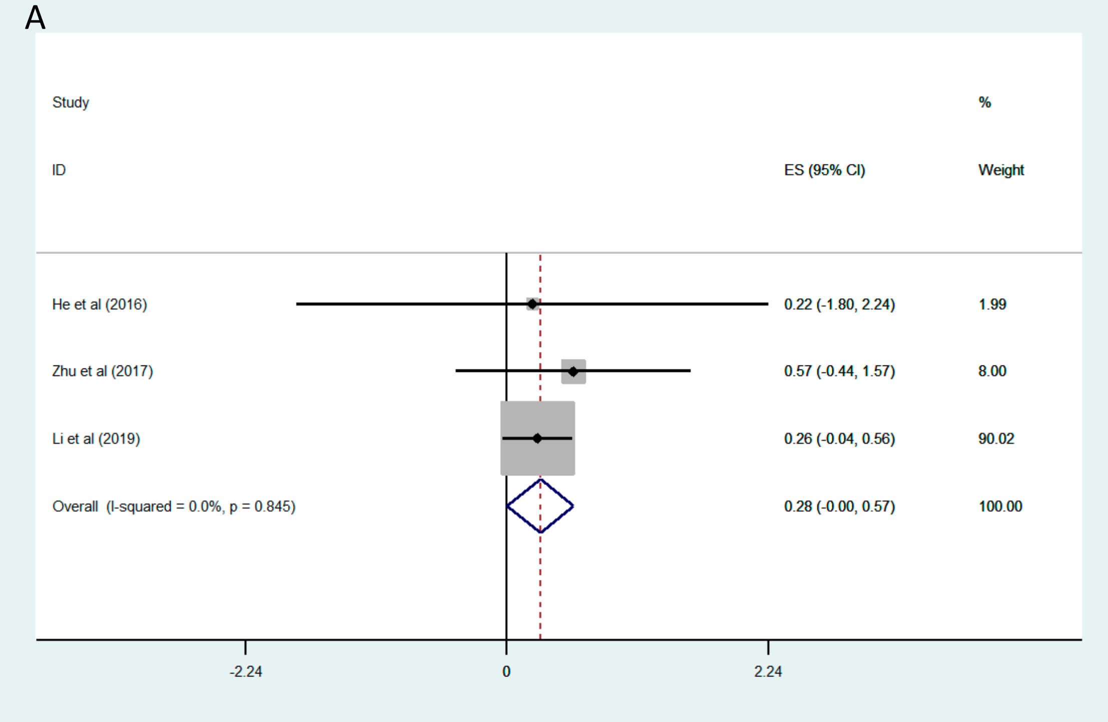

Study
ID
ES (95% CI)
%
Weight
He et al (2016)
0.22 (-1.80, 2.24)
1.99
Zhu et al (2017)
0.57 (-0.44, 1.57)
8.00
Li et al (2019)
0.26 (-0.04, 0.56)
90.02
Overall (I-squared = 0.0%, p = 0.845)
0.28 (-0.00, 0.57)
100.00
-2.24
0
2.24

B

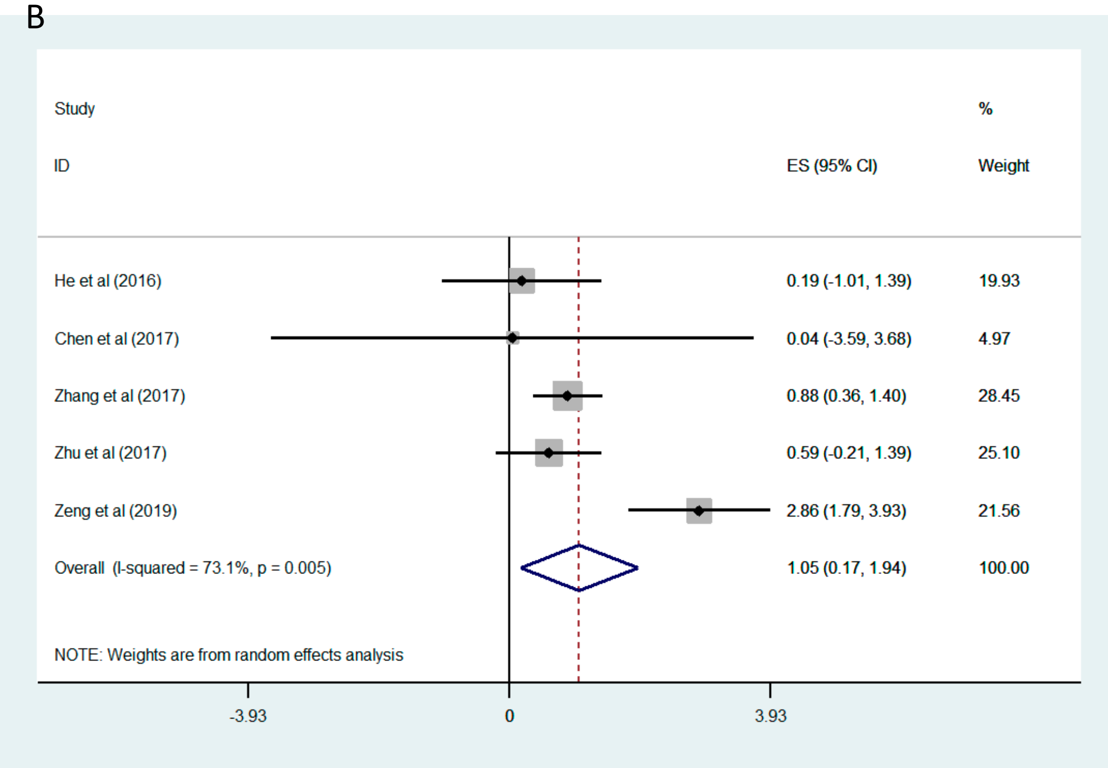

Study
ID
ES (95% CI)
%
Weight
He et al (2016)
0.19 (-1.01, 1.39)
19.93
Chen et al (2017)
0.04 (-3.59, 3.68)
4.97
Zhang et al (2017)
0.88 (0.36, 1.40)
28.45
Zhu et al (2017)
0.59 (-0.21, 1.39)
25.10
Zeng et al (2019)
2.86 (1.79, 3.93)
21.56
Overall (I-squared = 73.1%, p = 0.005)
1.05 (0.17, 1.94)
100.00
NOTE: Weights are from random effects analysis
-3.93
0
3.93

C

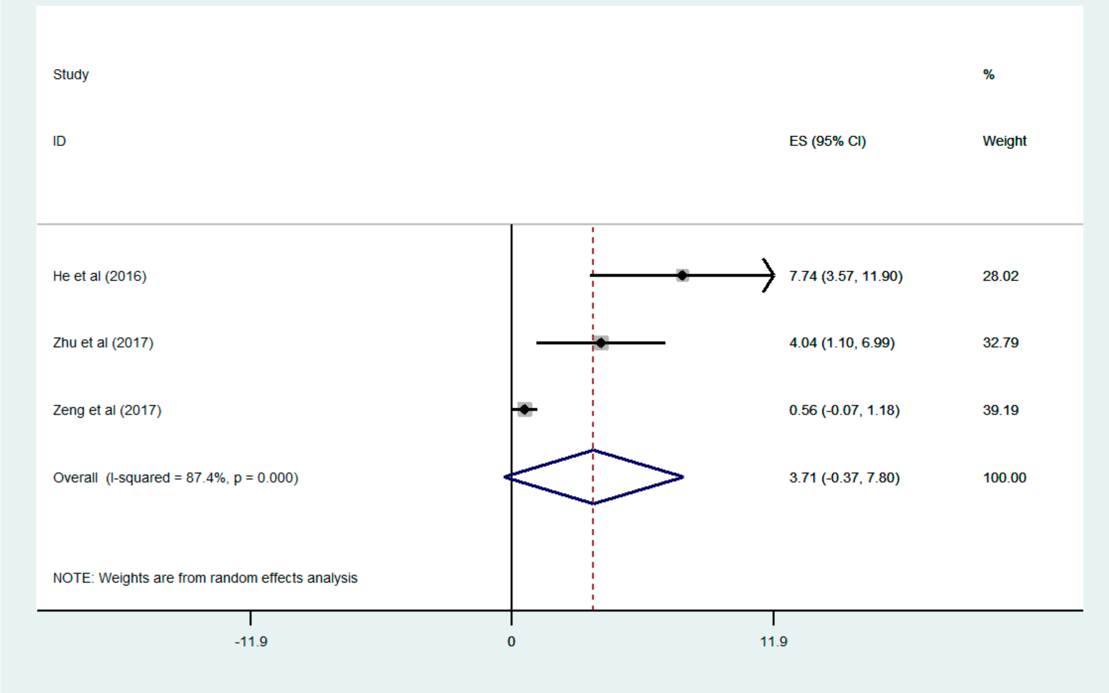

D

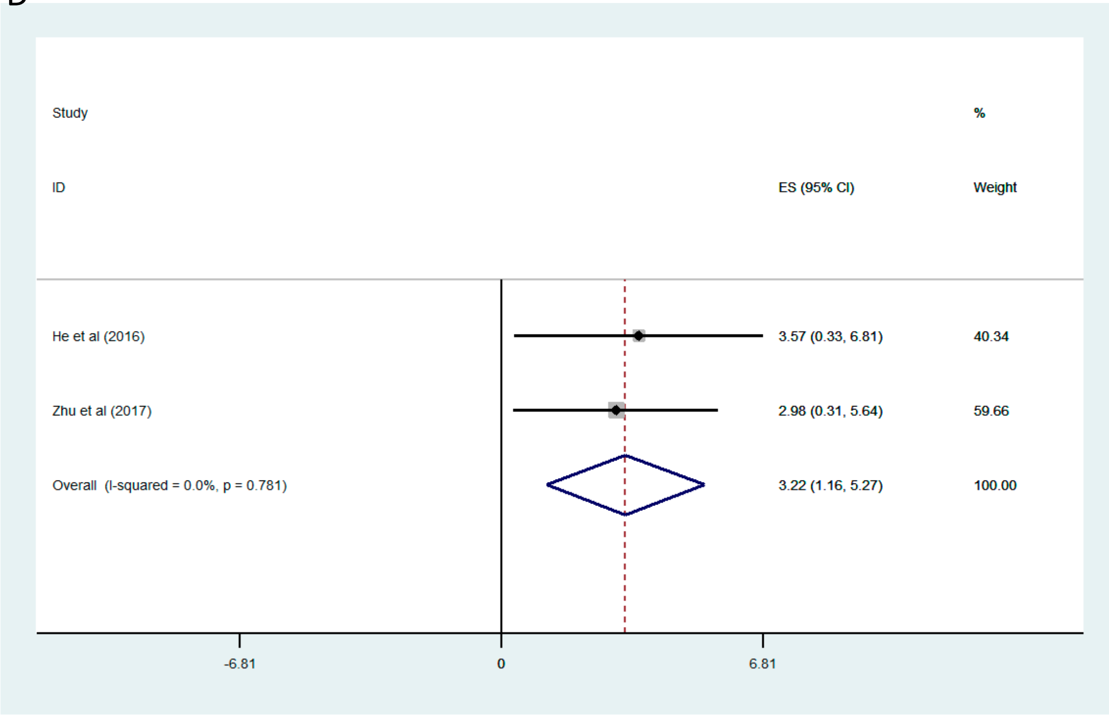

Figure S4 Forest plot and pooled effect estimates of the ambient air pollution on the YLL of CVD. A. $PM_{2.5}$; B. PM_{10}; C. SO_2; D. NO_2.

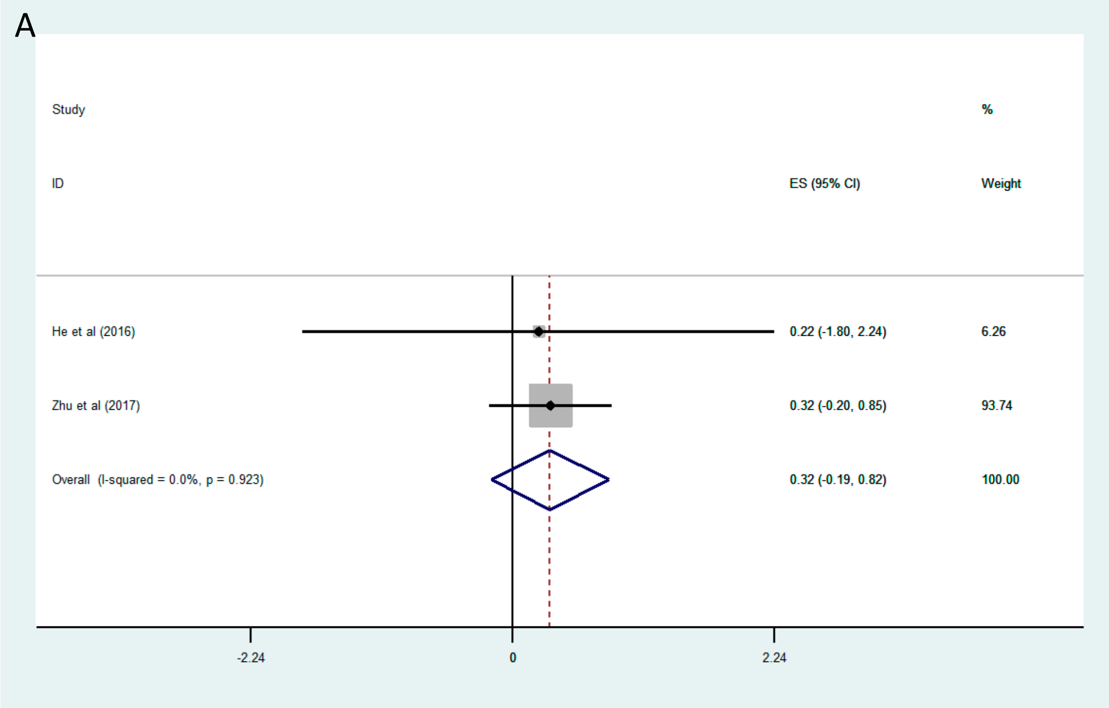
A
Study
ID
ES (95% CI)
%
Weight
He et al (2016)
0.22 (-1.80, 2.24)
6.26
Zhu et al (2017)
0.32 (-0.20, 0.85)
93.74
Overall (I-squared = 0.0%, p = 0.923)
0.32 (-0.19, 0.82)
100.00
-2.24
0
2.24

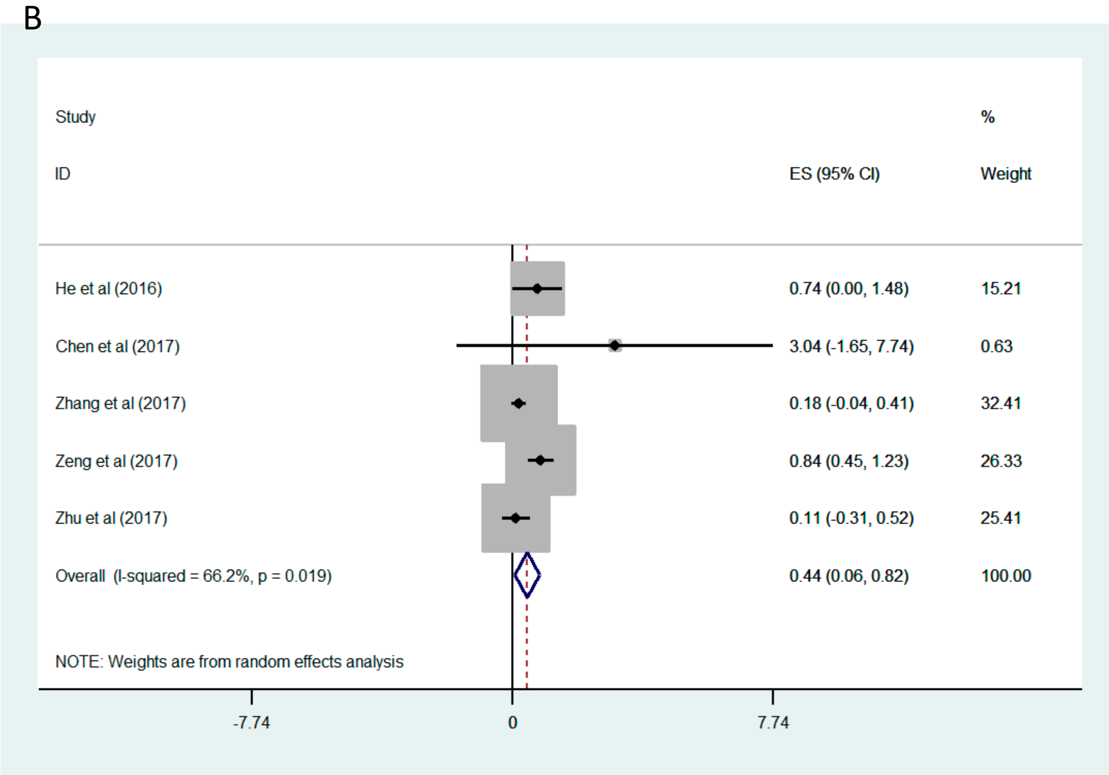
B
Study
ID
ES (95% CI)
%
Weight
He et al (2016)
0.74 (0.00, 1.48)
15.21
Chen et al (2017)
3.04 (-1.65, 7.74)
0.63
Zhang et al (2017)
0.18 (-0.04, 0.41)
32.41
Zeng et al (2017)
0.84 (0.45, 1.23)
26.33
Zhu et al (2017)
0.11 (-0.31, 0.52)
25.41
Overall (I-squared = 66.2%, p = 0.019)
0.44 (0.06, 0.82)
100.00
NOTE: Weights are from random effects analysis
-7.74
0
7.74

C

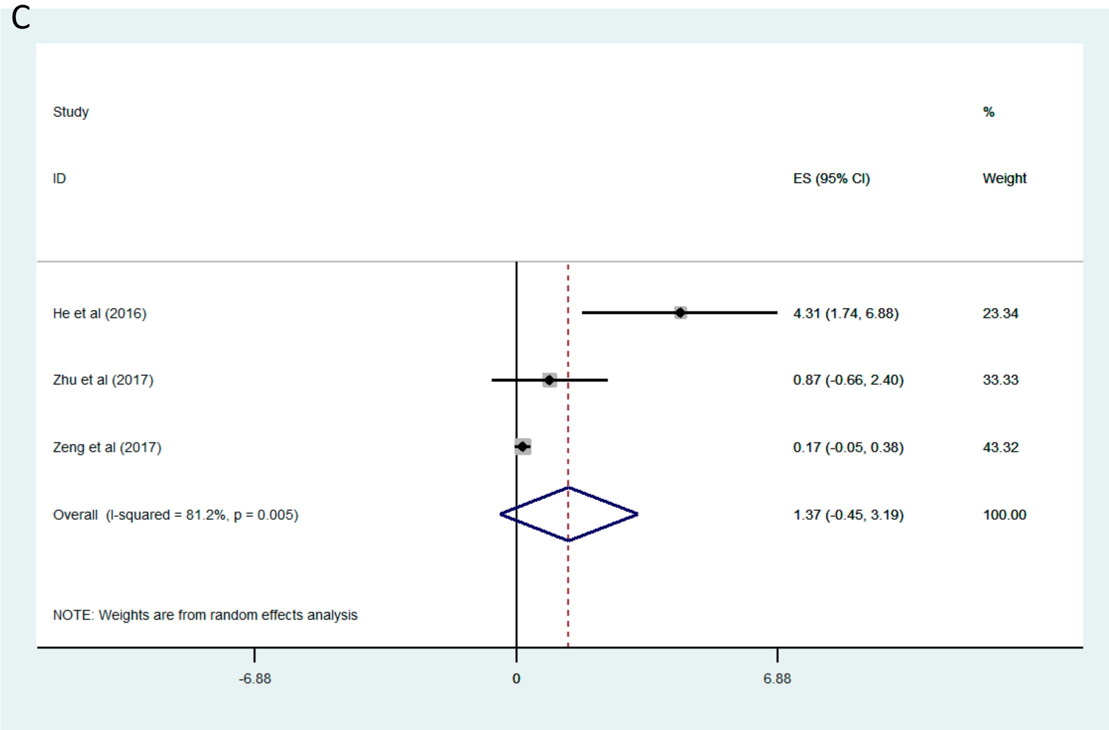

D

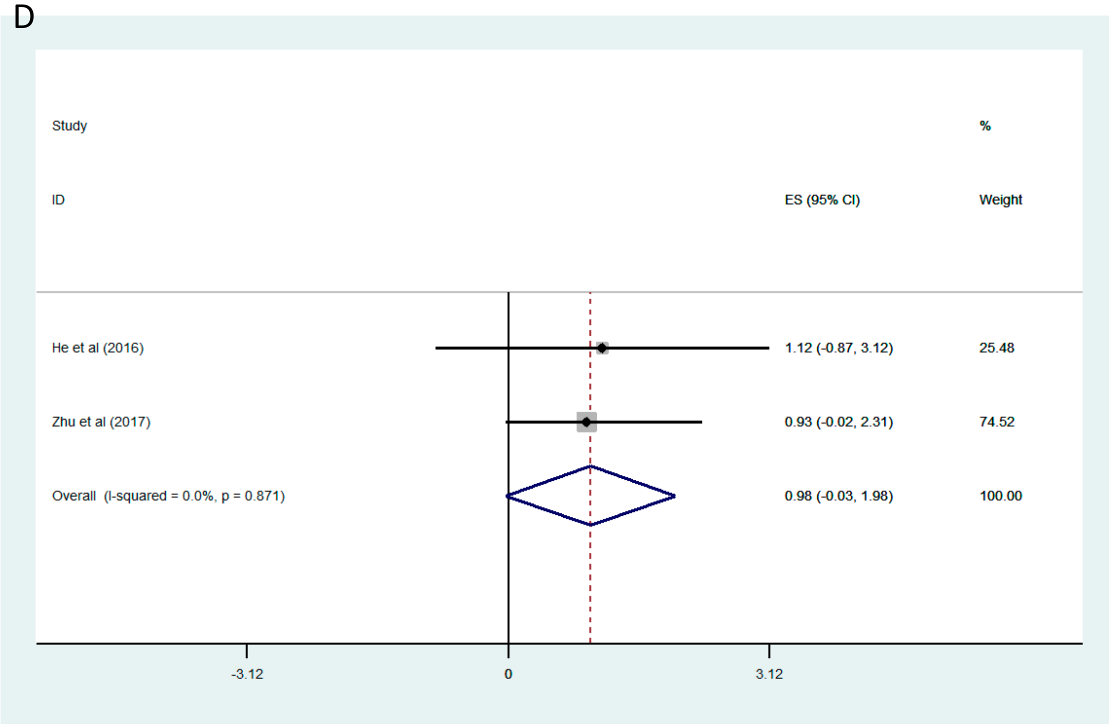

Figure S5 Forest plot and pooled effect estimates of the ambient air pollution on the YLL of RD. A. $PM_{2.5}$; B. PM_{10}; C. SO_2; D. NO_2.

A

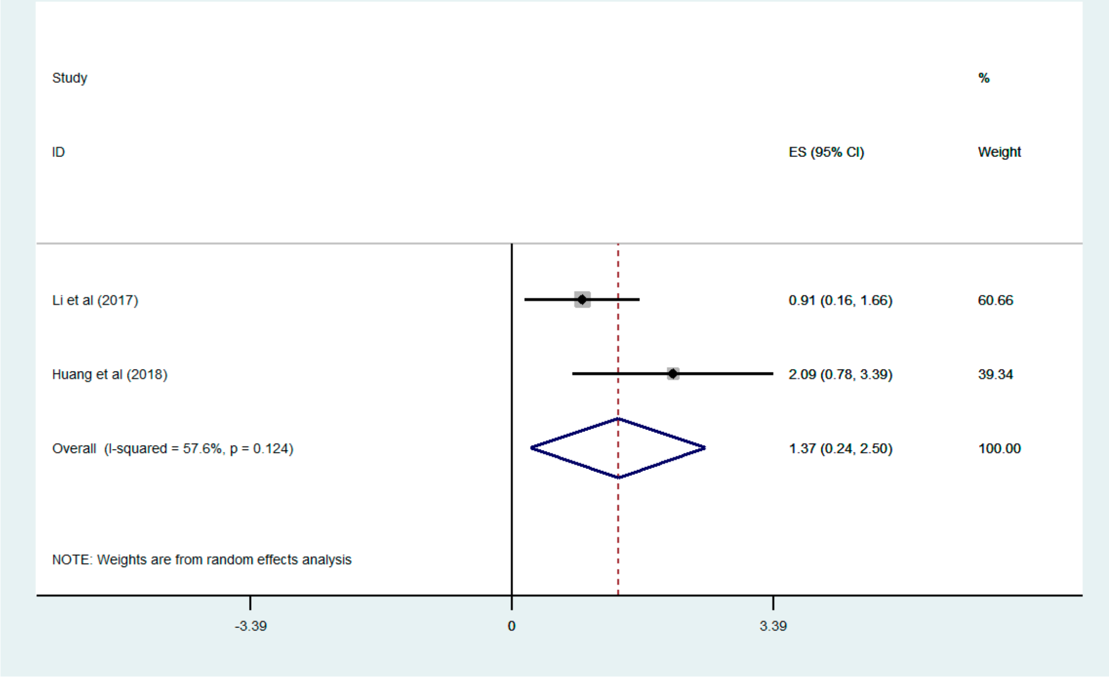
Study
ID
ES (95% CI)
%
Weight
Li et al (2017)
0.91 (0.16, 1.66)
60.66
Huang et al (2018)
2.09 (0.78, 3.39)
39.34
Overall (I-squared = 57.6%, p = 0.124)
1.37 (0.24, 2.50)
100.00
NOTE: Weights are from random effects analysis
-3.39
0
3.39

B

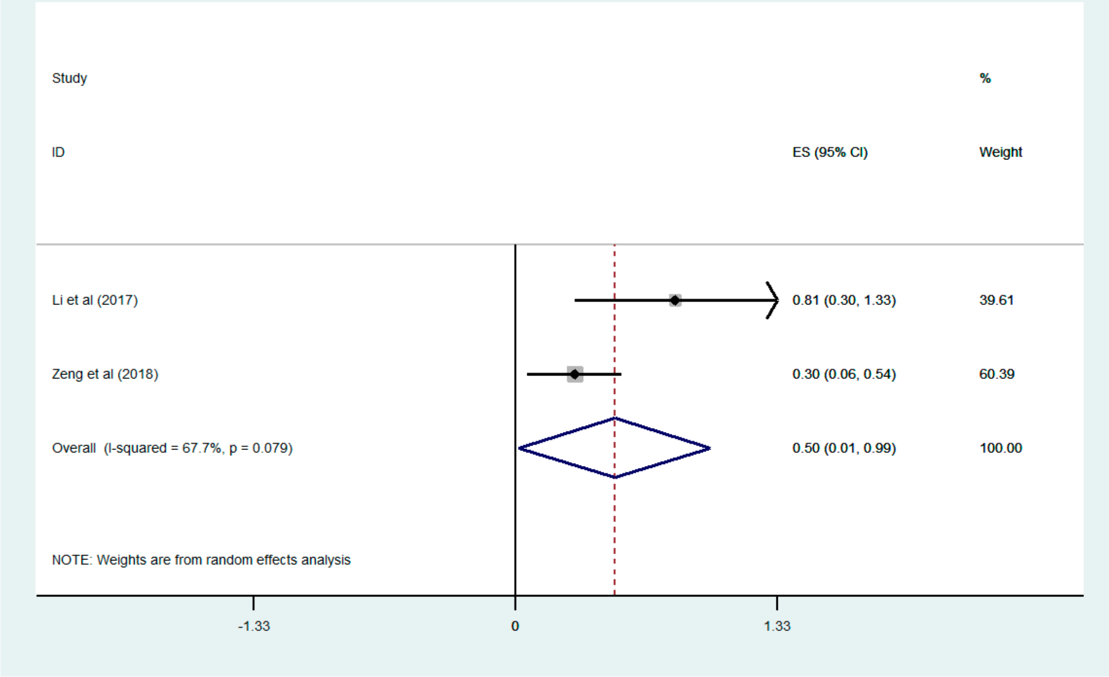
Study
ID
ES (95% CI)
%
Weight
Li et al (2017)
0.81 (0.30, 1.33)
39.61
Zeng et al (2018)
0.30 (0.06, 0.54)
60.39
Overall (I-squared = 67.7%, p = 0.079)
0.50 (0.01, 0.99)
100.00
NOTE: Weights are from random effects analysis
-1.33
0
1.33

C

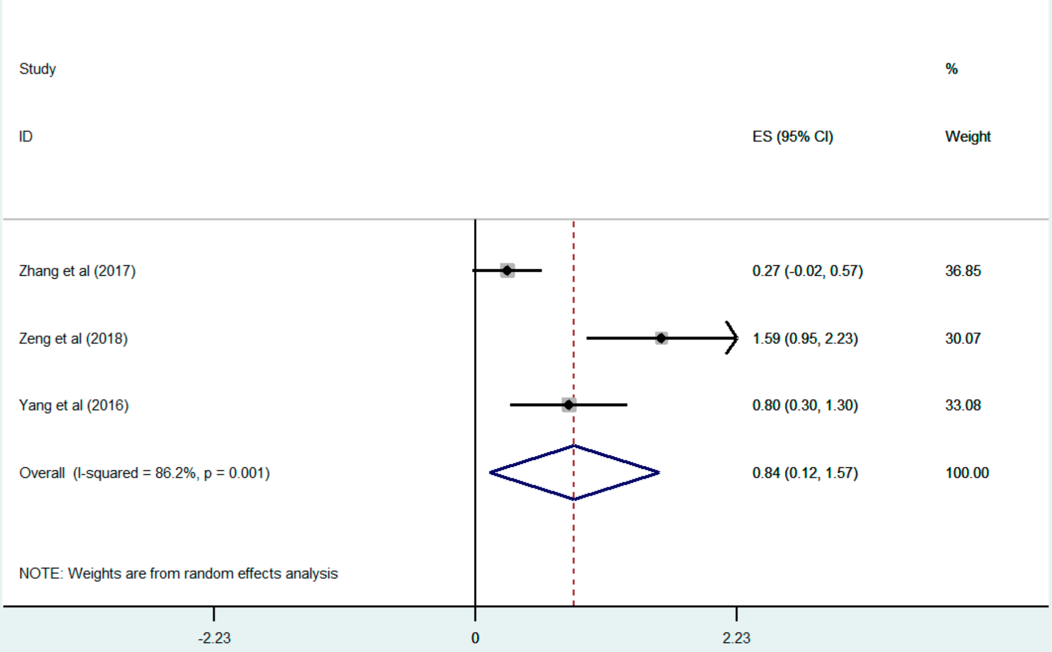
Study
ID
ES (95% CI)
%
Weight
Zhang et al (2017)
0.27 (-0.02, 0.57)
36.85
Zeng et al (2018)
1.59 (0.95, 2.23)
30.07
Yang et al (2016)
0.80 (0.30, 1.30)
33.08
Overall (I-squared = 86.2%, p = 0.001)
0.84 (0.12, 1.57)
100.00
NOTE: Weights are from random effects analysis
-2.23
0
2.23

D

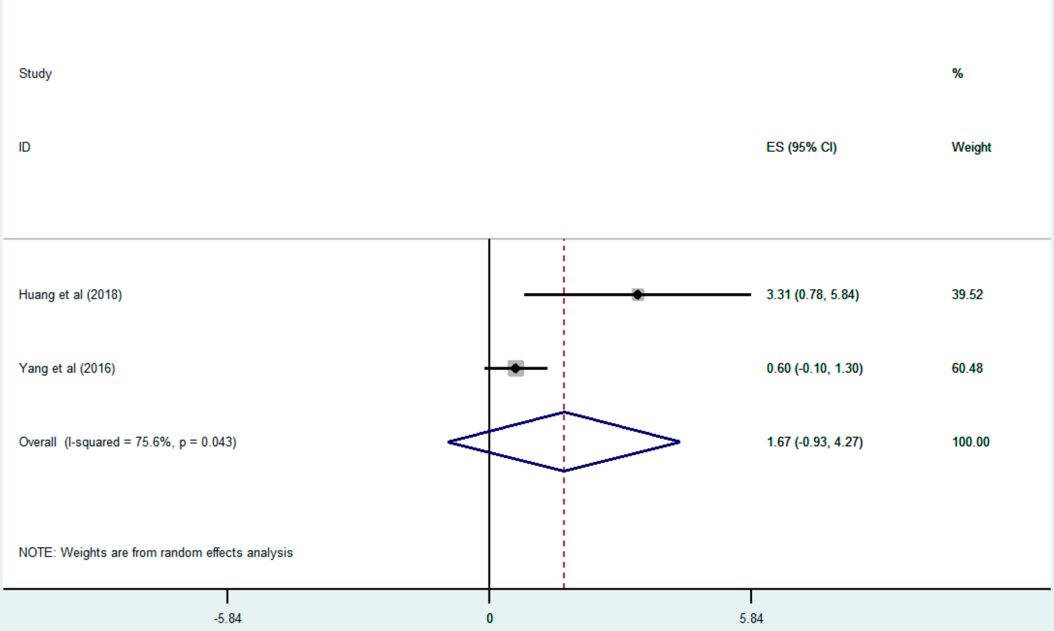
Study
ID
ES (95% CI)
%
Weight
Huang et al (2018)
3.31 (0.78, 5.84)
39.52
Yang et al (2016)
0.60 (-0.10, 1.30)
60.48
Overall (I-squared = 75.6%, p = 0.043)
1.67 (-0.93, 4.27)
100.00
NOTE: Weights are from random effects analysis
-5.84
0
5.84

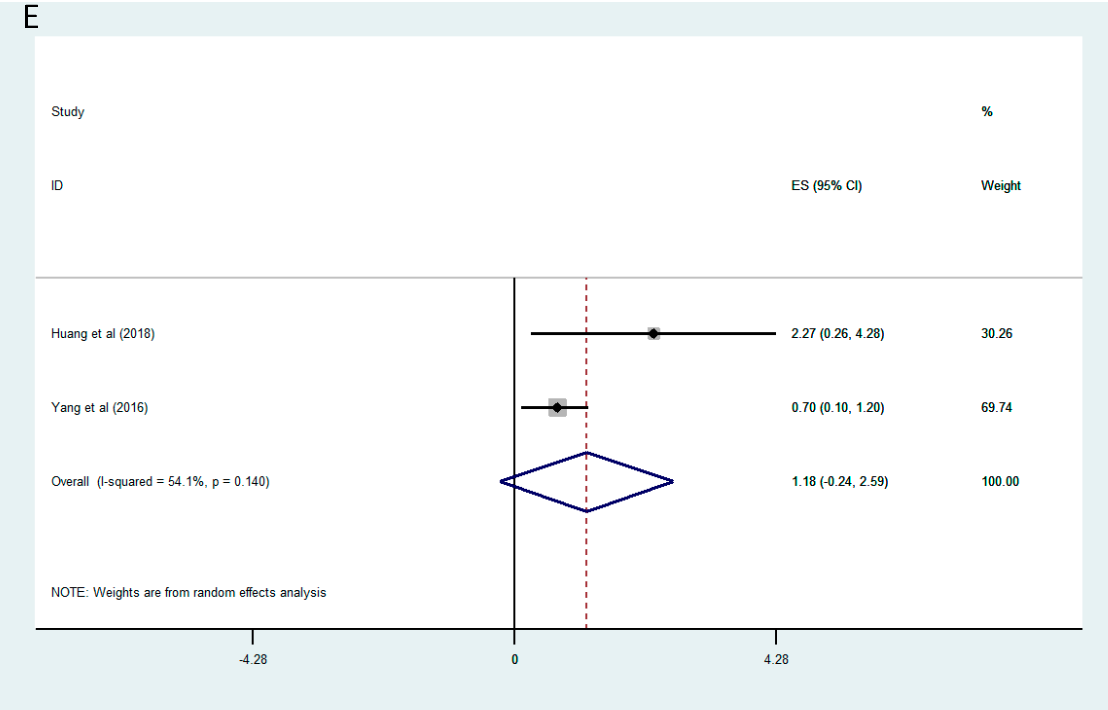

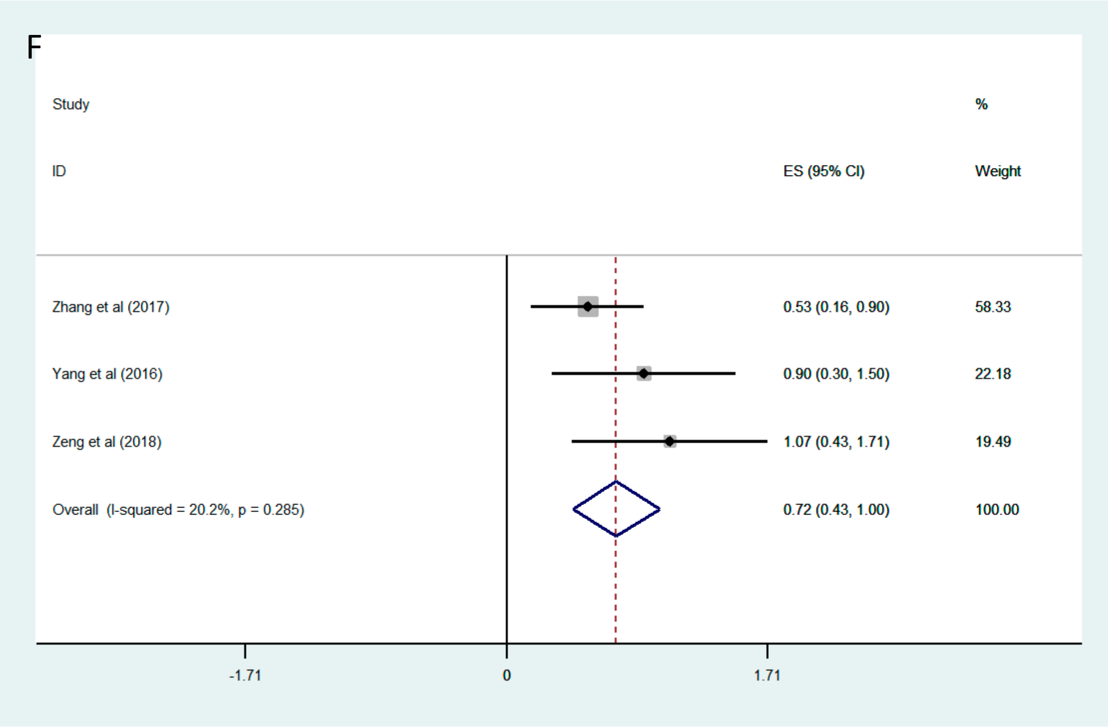

Figure S6 Forest plot and pooled effect estimates of the ambient air pollution on the YLL of other diseases. A. $PM_{2.5}$ and COPD; B. PM_{10} and COPD; C. PM_{10} and IHD; D. SO_2 and IHD; E. NO_2 and IHD; F.PM_{10} and stroke.

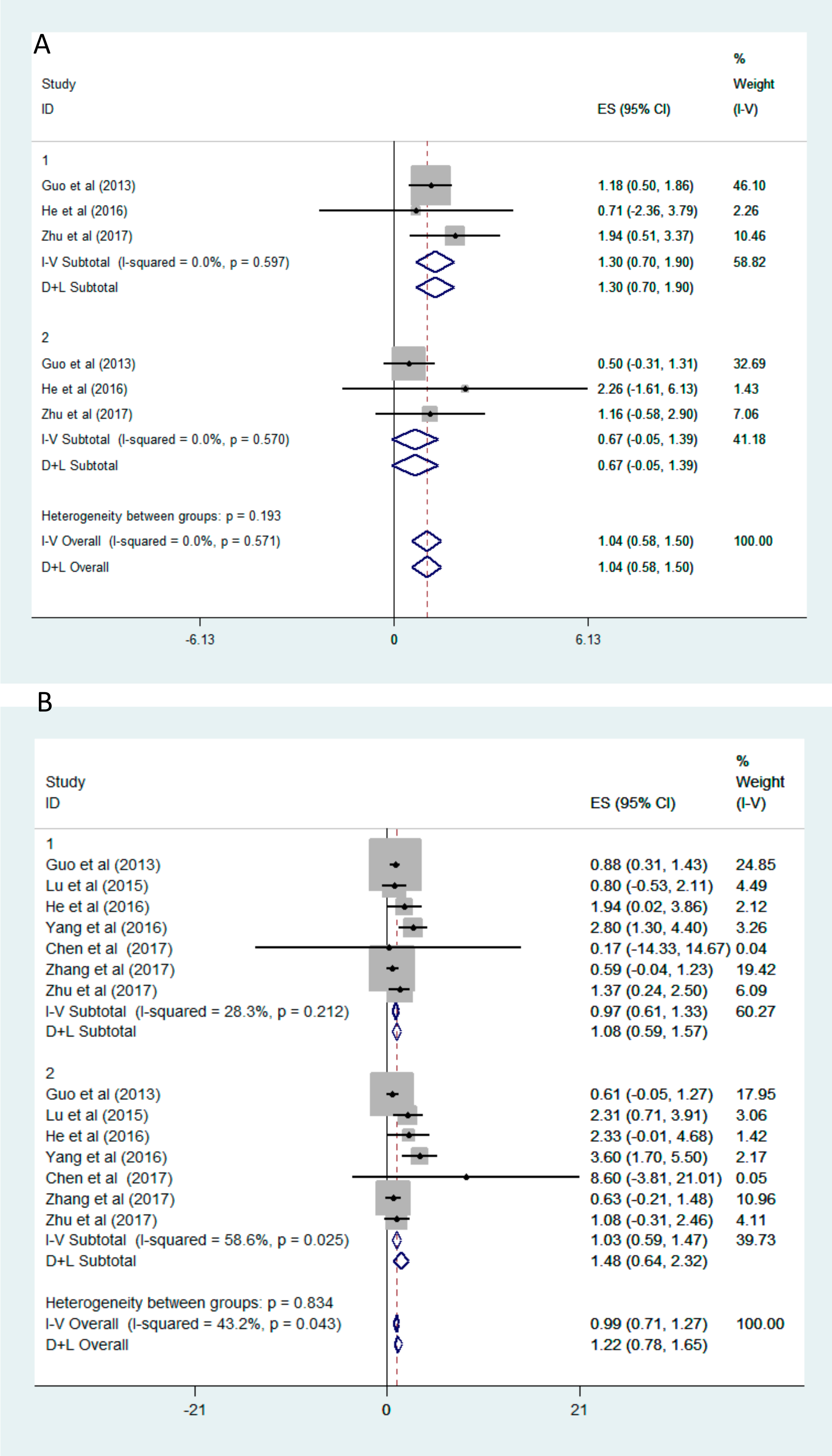
A
Study ID
ES (95% CI)
% Weight (I-V)
1
Guo et al (2013) 1.18 (0.50, 1.86) 46.10
He et al (2016) 0.71 (-2.36, 3.79) 2.26
Zhu et al (2017) 1.94 (0.51, 3.37) 10.46
I-V Subtotal (I-squared = 0.0%, p = 0.597) 1.30 (0.70, 1.90) 58.82
D+L Subtotal 1.30 (0.70, 1.90)
2
Guo et al (2013) 0.50 (-0.31, 1.31) 32.69
He et al (2016) 2.26 (-1.61, 6.13) 1.43
Zhu et al (2017) 1.16 (-0.58, 2.90) 7.06
I-V Subtotal (I-squared = 0.0%, p = 0.570) 0.67 (-0.05, 1.39) 41.18
D+L Subtotal 0.67 (-0.05, 1.39)
Heterogeneity between groups: p = 0.193
I-V Overall (I-squared = 0.0%, p = 0.571) 1.04 (0.58, 1.50) 100.00
D+L Overall 1.04 (0.58, 1.50)
-6.13
0
6.13
B
Study ID
ES (95% CI)
% Weight (I-V)
1
Guo et al (2013) 0.88 (0.31, 1.43) 24.85
Lu et al (2015) 0.80 (-0.53, 2.11) 4.49
He et al (2016) 1.94 (0.02, 3.86) 2.12
Yang et al (2016) 2.80 (1.30, 4.40) 3.26
Chen et al (2017) 0.17 (-14.33, 14.67) 0.04
Zhang et al (2017) 0.59 (-0.04, 1.23) 19.42
Zhu et al (2017) 1.37 (0.24, 2.50) 6.09
I-V Subtotal (I-squared = 28.3%, p = 0.212) 0.97 (0.61, 1.33) 60.27
D+L Subtotal 1.08 (0.59, 1.57)
2
Guo et al (2013) 0.61 (-0.05, 1.27) 17.95
Lu et al (2015) 2.31 (0.71, 3.91) 3.06
He et al (2016) 2.33 (-0.01, 4.68) 1.42
Yang et al (2016) 3.60 (1.70, 5.50) 2.17
Chen et al (2017) 8.60 (-3.81, 21.01) 0.05
Zhang et al (2017) 0.63 (-0.21, 1.48) 10.96
Zhu et al (2017) 1.08 (-0.31, 2.46) 4.11
I-V Subtotal (I-squared = 58.6%, p = 0.025) 1.03 (0.59, 1.47) 39.73
D+L Subtotal 1.48 (0.64, 2.32)
Heterogeneity between groups: p = 0.834
I-V Overall (I-squared = 43.2%, p = 0.043) 0.99 (0.71, 1.27) 100.00
D+L Overall 1.22 (0.78, 1.65)
-21
0
21

C

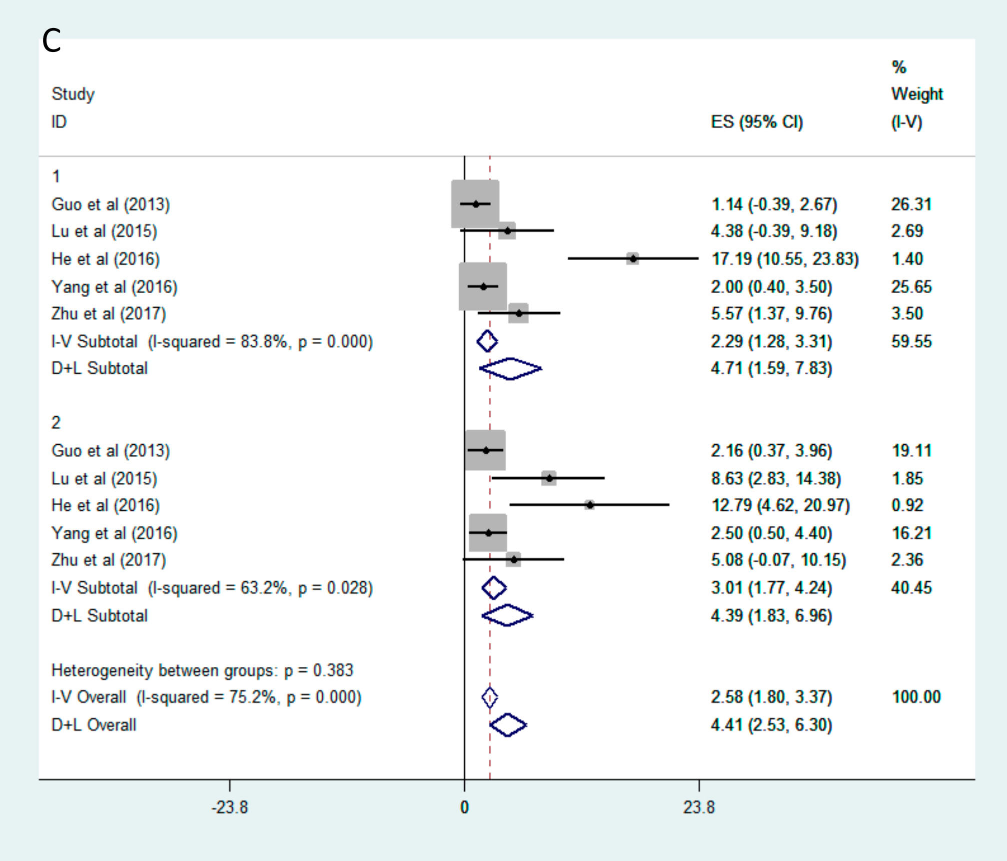
Study
ID
ES (95% CI)
%
Weight
(I-V)
1
Guo et al (2013) 1.14 (-0.39, 2.67) 26.31
Lu et al (2015) 4.38 (-0.39, 9.18) 2.69
He et al (2016) 17.19 (10.55, 23.83) 1.40
Yang et al (2016) 2.00 (0.40, 3.50) 25.65
Zhu et al (2017) 5.57 (1.37, 9.76) 3.50
I-V Subtotal (I-squared = 83.8%, p = 0.000) 2.29 (1.28, 3.31) 59.55
D+L Subtotal 4.71 (1.59, 7.83)
2
Guo et al (2013) 2.16 (0.37, 3.96) 19.11
Lu et al (2015) 8.63 (2.83, 14.38) 1.85
He et al (2016) 12.79 (4.62, 20.97) 0.92
Yang et al (2016) 2.50 (0.50, 4.40) 16.21
Zhu et al (2017) 5.08 (-0.07, 10.15) 2.36
I-V Subtotal (I-squared = 63.2%, p = 0.028) 3.01 (1.77, 4.24) 40.45
D+L Subtotal 4.39 (1.83, 6.96)
Heterogeneity between groups: p = 0.383
I-V Overall (I-squared = 75.2%, p = 0.000) 2.58 (1.80, 3.37) 100.00
D+L Overall 4.41 (2.53, 6.30)
-23.8
0
23.8

D

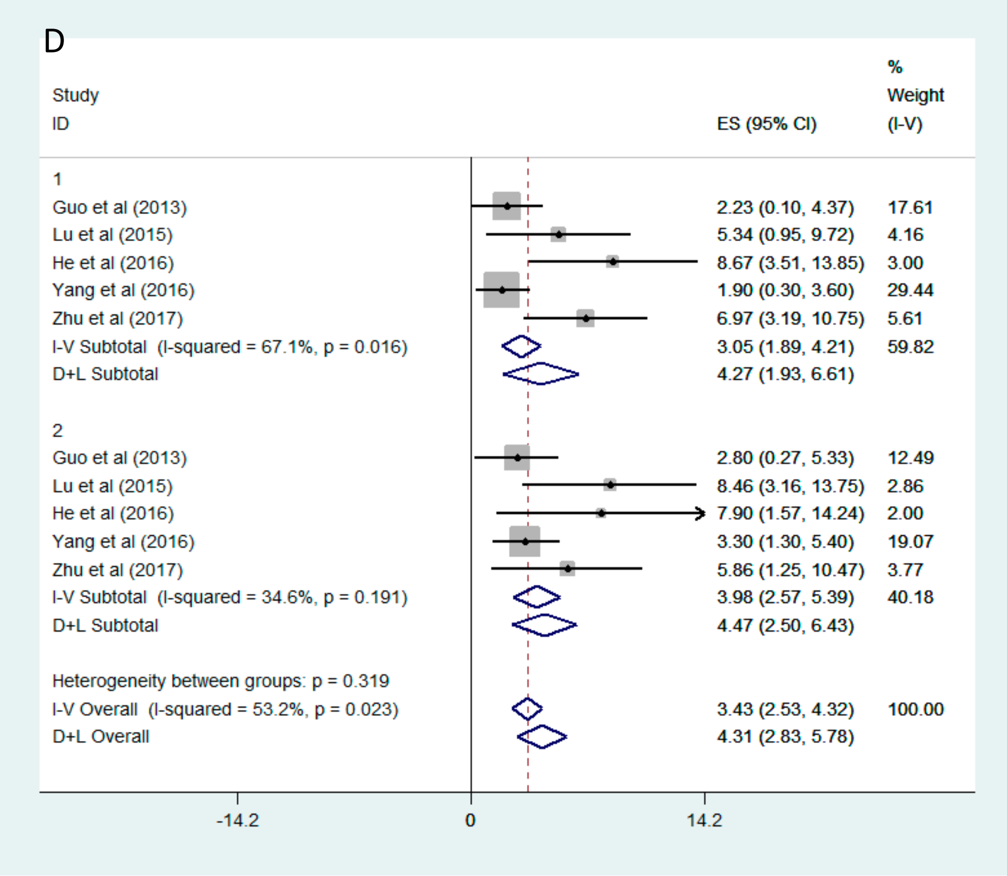
Study
ID
ES (95% CI)
%
Weight
(I-V)
1
Guo et al (2013) 2.23 (0.10, 4.37) 17.61
Lu et al (2015) 5.34 (0.95, 9.72) 4.16
He et al (2016) 8.67 (3.51, 13.85) 3.00
Yang et al (2016) 1.90 (0.30, 3.60) 29.44
Zhu et al (2017) 6.97 (3.19, 10.75) 5.61
I-V Subtotal (I-squared = 67.1%, p = 0.016) 3.05 (1.89, 4.21) 59.82
D+L Subtotal 4.27 (1.93, 6.61)
2
Guo et al (2013) 2.80 (0.27, 5.33) 12.49
Lu et al (2015) 8.46 (3.16, 13.75) 2.86
He et al (2016) 7.90 (1.57, 14.24) 2.00
Yang et al (2016) 3.30 (1.30, 5.40) 19.07
Zhu et al (2017) 5.86 (1.25, 10.47) 3.77
I-V Subtotal (I-squared = 34.6%, p = 0.191) 3.98 (2.57, 5.39) 40.18
D+L Subtotal 4.47 (2.50, 6.43)
Heterogeneity between groups: p = 0.319
I-V Overall (I-squared = 53.2%, p = 0.023) 3.43 (2.53, 4.32) 100.00
D+L Overall 4.31 (2.83, 5.78)
-14.2
0
14.2

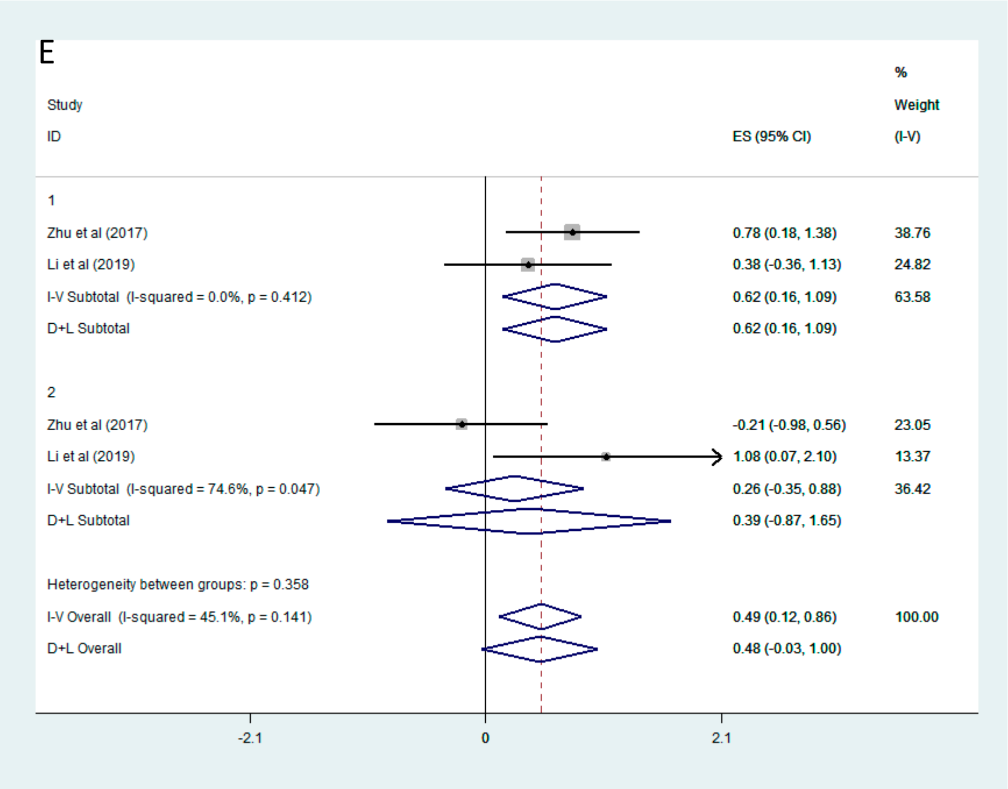
E
%
Study
Weight
ID
ES (95% CI)
(I-V)
1
Zhu et al (2017)
0.78 (0.18, 1.38)
38.76
Li et al (2019)
0.38 (-0.36, 1.13)
24.82
I-V Subtotal (I-squared = 0.0%, p = 0.412)
0.62 (0.16, 1.09)
63.58
D+L Subtotal
0.62 (0.16, 1.09)
2
Zhu et al (2017)
-0.21 (-0.98, 0.56)
23.05
Li et al (2019)
1.08 (0.07, 2.10)
13.37
I-V Subtotal (I-squared = 74.6%, p = 0.047)
0.26 (-0.35, 0.88)
36.42
D+L Subtotal
0.39 (-0.87, 1.65)
Heterogeneity between groups: p = 0.358
I-V Overall (I-squared = 45.1%, p = 0.141)
0.49 (0.12, 0.86)
100.00
D+L Overall
0.48 (-0.03, 1.00)
-2.1
0
2.1

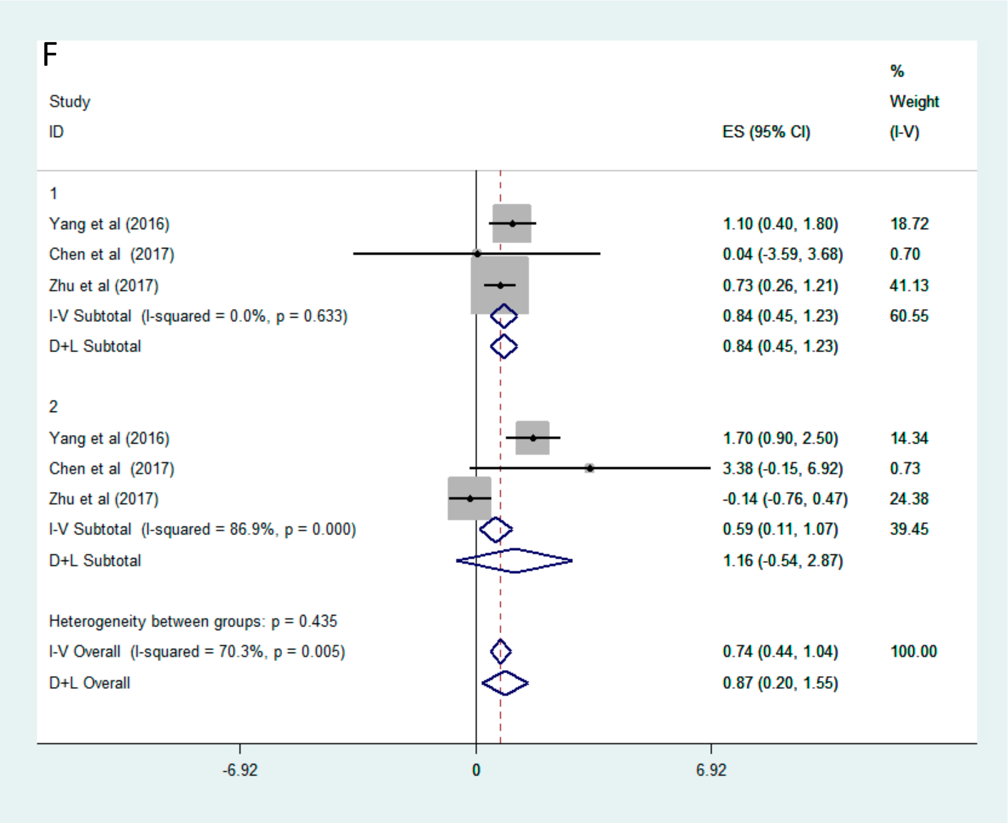
F
%
Study
Weight
ID
ES (95% CI)
(I-V)
1
Yang et al (2016)
1.10 (0.40, 1.80)
18.72
Chen et al (2017)
0.04 (-3.59, 3.68)
0.70
Zhu et al (2017)
0.73 (0.26, 1.21)
41.13
I-V Subtotal (I-squared = 0.0%, p = 0.633)
0.84 (0.45, 1.23)
60.55
D+L Subtotal
0.84 (0.45, 1.23)
2
Yang et al (2016)
1.70 (0.90, 2.50)
14.34
Chen et al (2017)
3.38 (-0.15, 6.92)
0.73
Zhu et al (2017)
-0.14 (-0.76, 0.47)
24.38
I-V Subtotal (I-squared = 86.9%, p = 0.000)
0.59 (0.11, 1.07)
39.45
D+L Subtotal
1.16 (-0.54, 2.87)
Heterogeneity between groups: p = 0.435
I-V Overall (I-squared = 70.3%, p = 0.005)
0.74 (0.44, 1.04)
100.00
D+L Overall
0.87 (0.20, 1.55)
-6.92
0
6.92

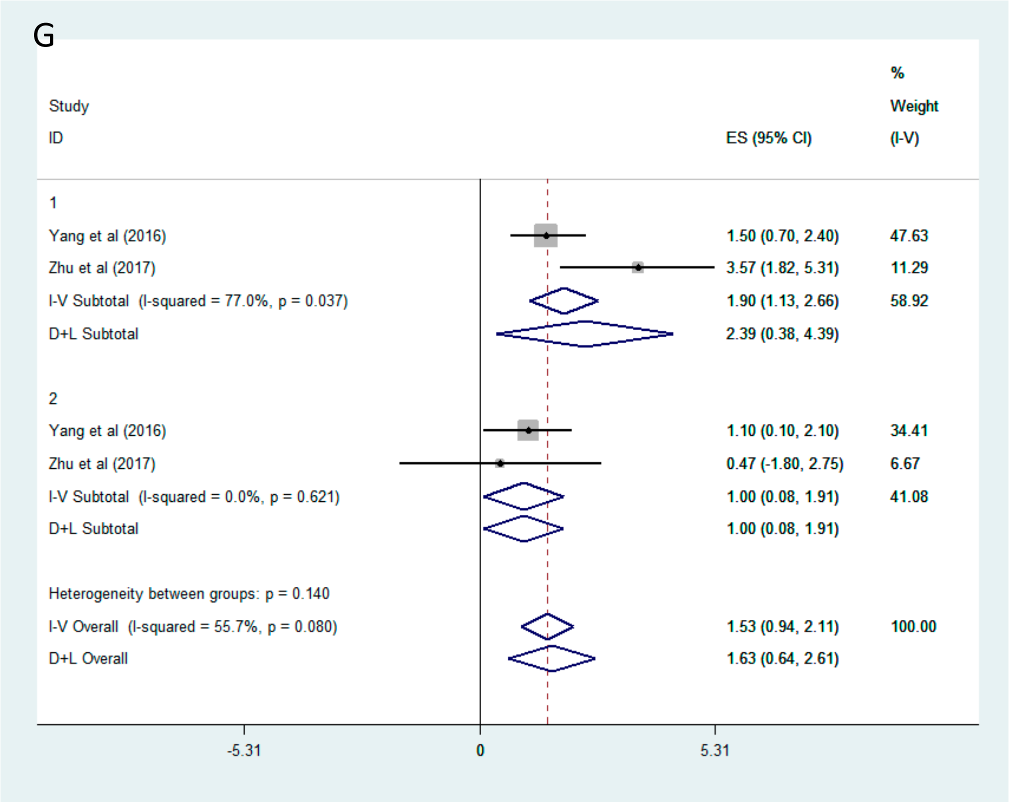
G
Study
ID
ES (95% CI)
%
Weight
(I-V)
1
Yang et al (2016)
1.50 (0.70, 2.40)
47.63
Zhu et al (2017)
3.57 (1.82, 5.31)
11.29
I-V Subtotal (I-squared = 77.0%, p = 0.037)
1.90 (1.13, 2.66)
58.92
D+L Subtotal
2.39 (0.38, 4.39)
2
Yang et al (2016)
1.10 (0.10, 2.10)
34.41
Zhu et al (2017)
0.47 (-1.80, 2.75)
6.67
I-V Subtotal (I-squared = 0.0%, p = 0.621)
1.00 (0.08, 1.91)
41.08
D+L Subtotal
1.00 (0.08, 1.91)
Heterogeneity between groups: p = 0.140
I-V Overall (I-squared = 55.7%, p = 0.080)
1.53 (0.94, 2.11)
100.00
D+L Overall
1.63 (0.64, 2.61)
-5.31
0
5.31

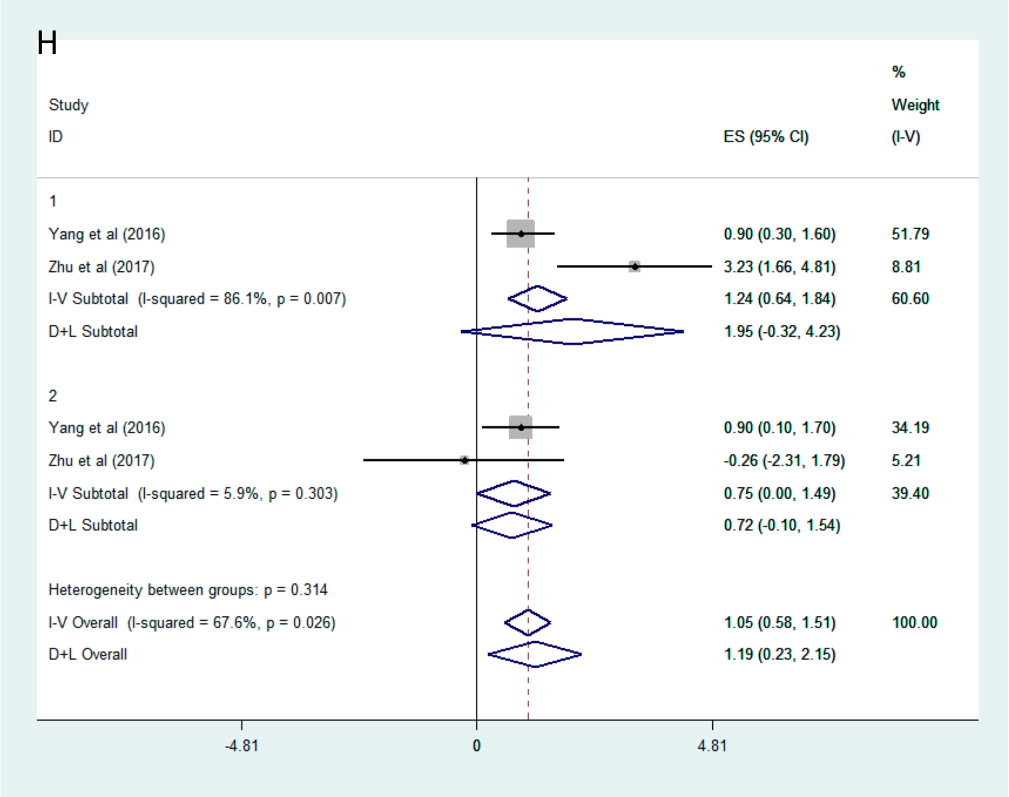
H
Study
ID
ES (95% CI)
%
Weight
(I-V)
1
Yang et al (2016)
0.90 (0.30, 1.60)
51.79
Zhu et al (2017)
3.23 (1.66, 4.81)
8.81
I-V Subtotal (I-squared = 86.1%, p = 0.007)
1.24 (0.64, 1.84)
60.60
D+L Subtotal
1.95 (-0.32, 4.23)
2
Yang et al (2016)
0.90 (0.10, 1.70)
34.19
Zhu et al (2017)
-0.26 (-2.31, 1.79)
5.21
I-V Subtotal (I-squared = 5.9%, p = 0.303)
0.75 (0.00, 1.49)
39.40
D+L Subtotal
0.72 (-0.10, 1.54)
Heterogeneity between groups: p = 0.314
I-V Overall (I-squared = 67.6%, p = 0.026)
1.05 (0.58, 1.51)
100.00
D+L Overall
1.19 (0.23, 2.15)
-4.81
0
4.81

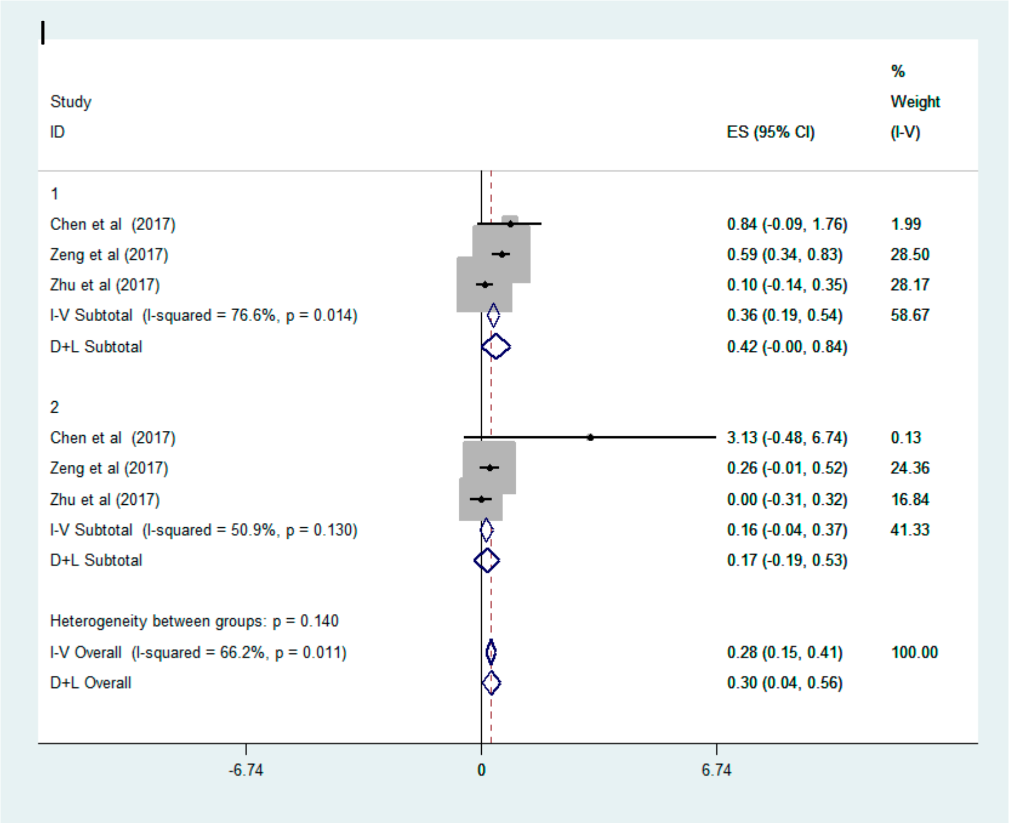

Figure S7 Forest plot and pooled effect estimates of the ambient air pollution on YLL for non-accidental diseases, cardiovascular disease (CVD) and respiratory disease (RD)stratified by gender. A. $PM_{2.5}$ and non-accidental disease; B. PM_{10} and non-accidental disease; C. SO_2 and non-accidental disease; D. NO_2 and non-accidental disease; E. $PM_{2.5}$ and CVD; F.PM_{10} and CVD; G. SO_2 and CVD; H. NO_2 and CVD; I. PM_{10} and RD.Group 1: female; group 2: Male.

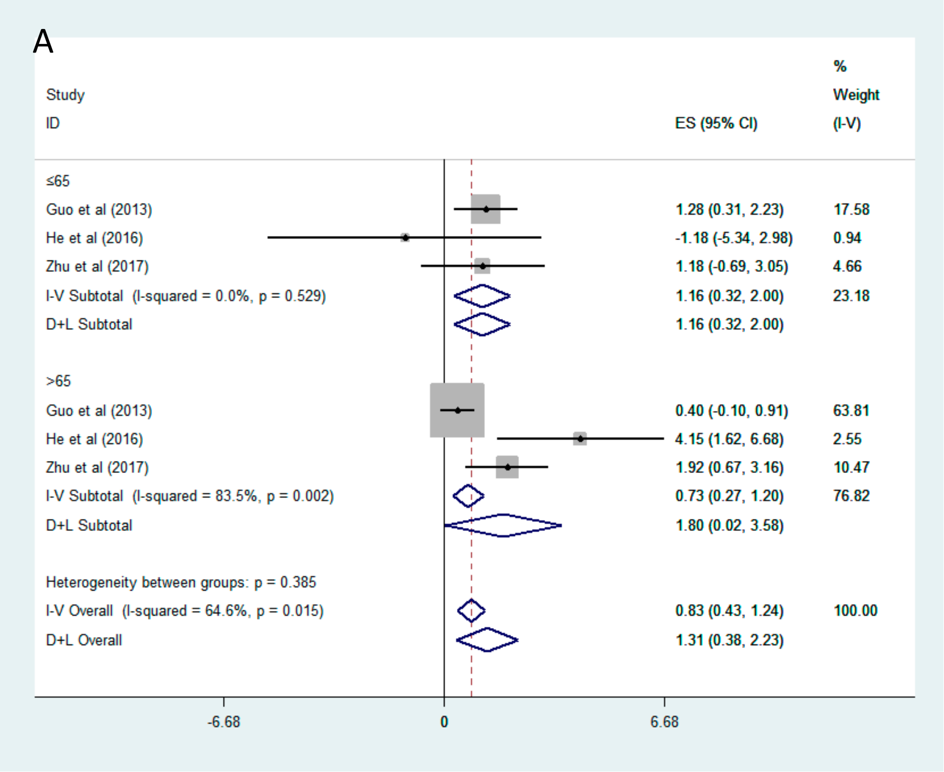
A
Study ID
ES (95% CI)
% Weight (I-V)
≤65
Guo et al (2013) 1.28 (0.31, 2.23) 17.58
He et al (2016) -1.18 (-5.34, 2.98) 0.94
Zhu et al (2017) 1.18 (-0.69, 3.05) 4.66
I-V Subtotal (I-squared = 0.0%, p = 0.529) 1.16 (0.32, 2.00) 23.18
D+L Subtotal 1.16 (0.32, 2.00)
>65
Guo et al (2013) 0.40 (-0.10, 0.91) 63.81
He et al (2016) 4.15 (1.62, 6.68) 2.55
Zhu et al (2017) 1.92 (0.67, 3.16) 10.47
I-V Subtotal (I-squared = 83.5%, p = 0.002) 0.73 (0.27, 1.20) 76.82
D+L Subtotal 1.80 (0.02, 3.58)
Heterogeneity between groups: p = 0.385
I-V Overall (I-squared = 64.6%, p = 0.015) 0.83 (0.43, 1.24) 100.00
D+L Overall 1.31 (0.38, 2.23)
-6.68
0
6.68

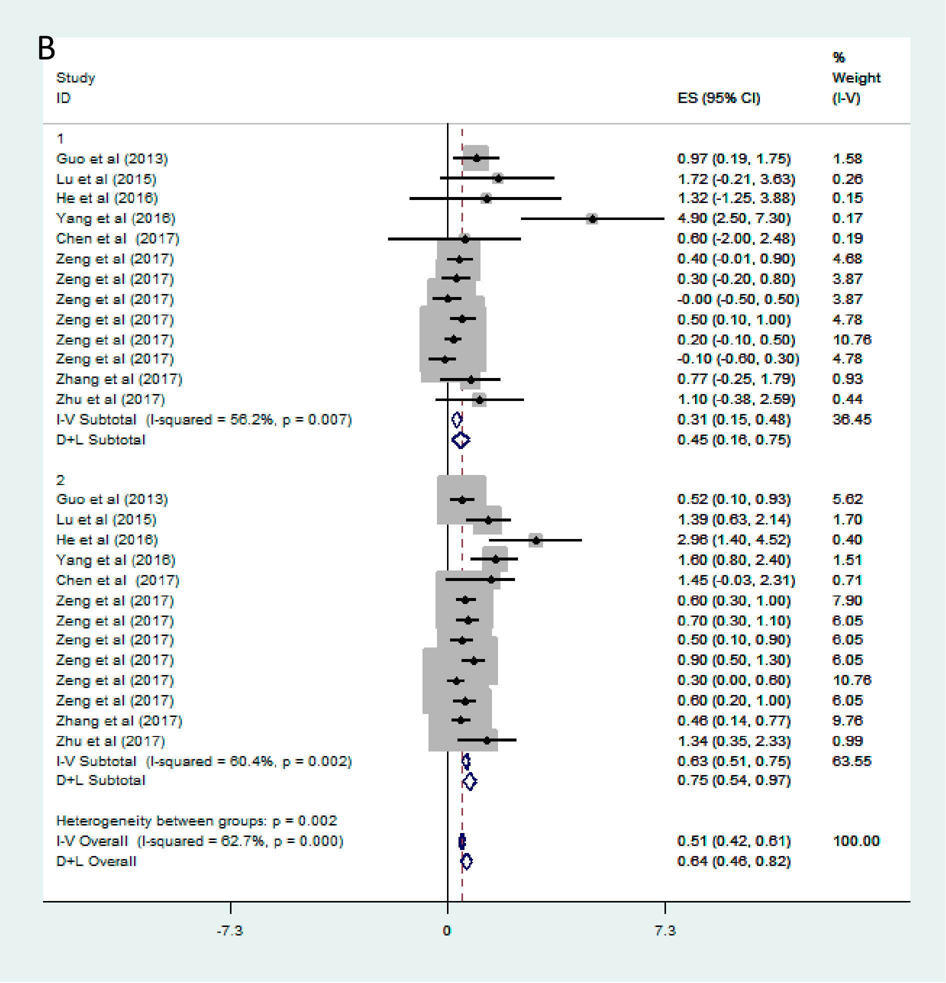
B
Study ID
ES (95% CI)
% Weight (I-V)
1
Guo et al (2013) 0.97 (0.19, 1.75) 1.58
Lu et al (2015) 1.72 (-0.21, 3.63) 0.26
He et al (2016) 1.32 (-1.25, 3.88) 0.15
Yang et al (2016) 4.90 (2.50, 7.30) 0.17
Chen et al (2017) 0.60 (-2.00, 2.48) 0.19
Zeng et al (2017) 0.40 (-0.01, 0.90) 4.68
Zeng et al (2017) 0.30 (-0.20, 0.80) 3.87
Zeng et al (2017) -0.00 (-0.50, 0.50) 3.87
Zeng et al (2017) 0.50 (0.10, 1.00) 4.78
Zeng et al (2017) 0.20 (-0.10, 0.50) 10.76
Zeng et al (2017) -0.10 (-0.60, 0.30) 4.78
Zhang et al (2017) 0.77 (-0.25, 1.79) 0.93
Zhu et al (2017) 1.10 (-0.38, 2.59) 0.44
I-V Subtotal (I-squared = 56.2%, p = 0.007) 0.31 (0.15, 0.48) 36.45
D+L Subtotal 0.45 (0.16, 0.75)
2
Guo et al (2013) 0.52 (0.10, 0.93) 5.62
Lu et al (2015) 1.39 (0.63, 2.14) 1.70
He et al (2016) 2.96 (1.40, 4.52) 0.40
Yang et al (2016) 1.60 (0.80, 2.40) 1.51
Chen et al (2017) 1.45 (-0.03, 2.31) 0.71
Zeng et al (2017) 0.60 (0.30, 1.00) 7.90
Zeng et al (2017) 0.70 (0.30, 1.10) 6.05
Zeng et al (2017) 0.50 (0.10, 0.90) 6.05
Zeng et al (2017) 0.90 (0.50, 1.30) 6.05
Zeng et al (2017) 0.30 (0.00, 0.60) 10.76
Zeng et al (2017) 0.60 (0.20, 1.00) 6.05
Zhang et al (2017) 0.46 (0.14, 0.77) 9.76
Zhu et al (2017) 1.34 (0.35, 2.33) 0.99
I-V Subtotal (I-squared = 60.4%, p = 0.002) 0.63 (0.51, 0.75) 63.55
D+L Subtotal 0.75 (0.54, 0.97)
Heterogeneity between groups: p = 0.002
I-V Overall (I-squared = 62.7%, p = 0.000) 0.51 (0.42, 0.61) 100.00
D+L Overall 0.64 (0.46, 0.82)
-7.3
0
7.3

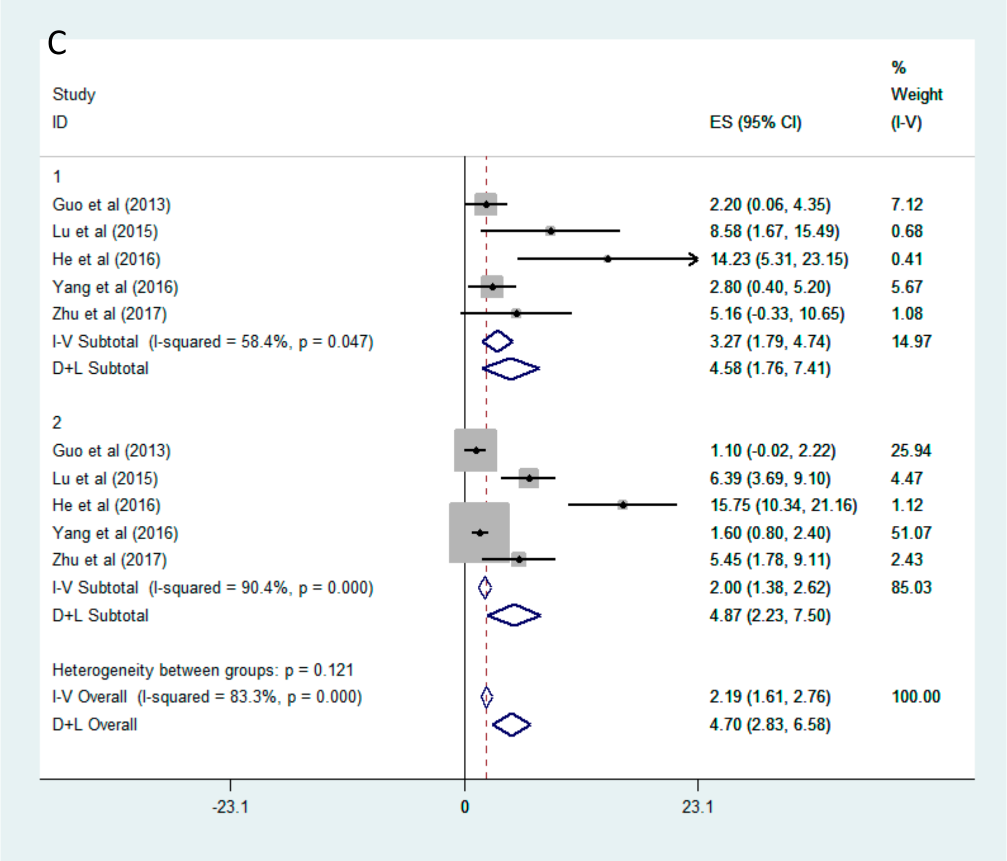
C
Study
ID
ES (95% CI)
%
Weight
(I-V)
1
Guo et al (2013) 2.20 (0.06, 4.35) 7.12
Lu et al (2015) 8.58 (1.67, 15.49) 0.68
He et al (2016) 14.23 (5.31, 23.15) 0.41
Yang et al (2016) 2.80 (0.40, 5.20) 5.67
Zhu et al (2017) 5.16 (-0.33, 10.65) 1.08
I-V Subtotal (I-squared = 58.4%, p = 0.047) 3.27 (1.79, 4.74) 14.97
D+L Subtotal 4.58 (1.76, 7.41)
2
Guo et al (2013) 1.10 (-0.02, 2.22) 25.94
Lu et al (2015) 6.39 (3.69, 9.10) 4.47
He et al (2016) 15.75 (10.34, 21.16) 1.12
Yang et al (2016) 1.60 (0.80, 2.40) 51.07
Zhu et al (2017) 5.45 (1.78, 9.11) 2.43
I-V Subtotal (I-squared = 90.4%, p = 0.000) 2.00 (1.38, 2.62) 85.03
D+L Subtotal 4.87 (2.23, 7.50)
Heterogeneity between groups: p = 0.121
I-V Overall (I-squared = 83.3%, p = 0.000) 2.19 (1.61, 2.76) 100.00
D+L Overall 4.70 (2.83, 6.58)
-23.1
0
23.1

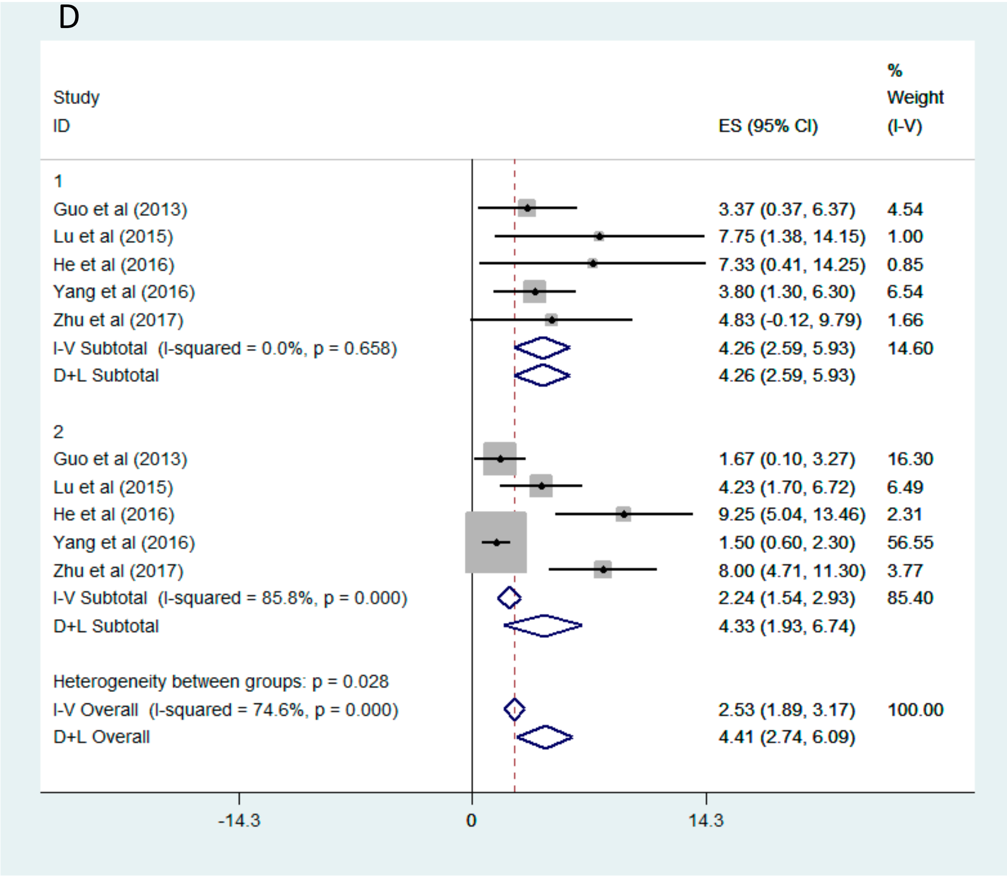
D
Study
ID
ES (95% CI)
%
Weight
(I-V)
1
Guo et al (2013) 3.37 (0.37, 6.37) 4.54
Lu et al (2015) 7.75 (1.38, 14.15) 1.00
He et al (2016) 7.33 (0.41, 14.25) 0.85
Yang et al (2016) 3.80 (1.30, 6.30) 6.54
Zhu et al (2017) 4.83 (-0.12, 9.79) 1.66
I-V Subtotal (I-squared = 0.0%, p = 0.658) 4.26 (2.59, 5.93) 14.60
D+L Subtotal 4.26 (2.59, 5.93)
2
Guo et al (2013) 1.67 (0.10, 3.27) 16.30
Lu et al (2015) 4.23 (1.70, 6.72) 6.49
He et al (2016) 9.25 (5.04, 13.46) 2.31
Yang et al (2016) 1.50 (0.60, 2.30) 56.55
Zhu et al (2017) 8.00 (4.71, 11.30) 3.77
I-V Subtotal (I-squared = 85.8%, p = 0.000) 2.24 (1.54, 2.93) 85.40
D+L Subtotal 4.33 (1.93, 6.74)
Heterogeneity between groups: p = 0.028
I-V Overall (I-squared = 74.6%, p = 0.000) 2.53 (1.89, 3.17) 100.00
D+L Overall 4.41 (2.74, 6.09)
-14.3
0
14.3

E

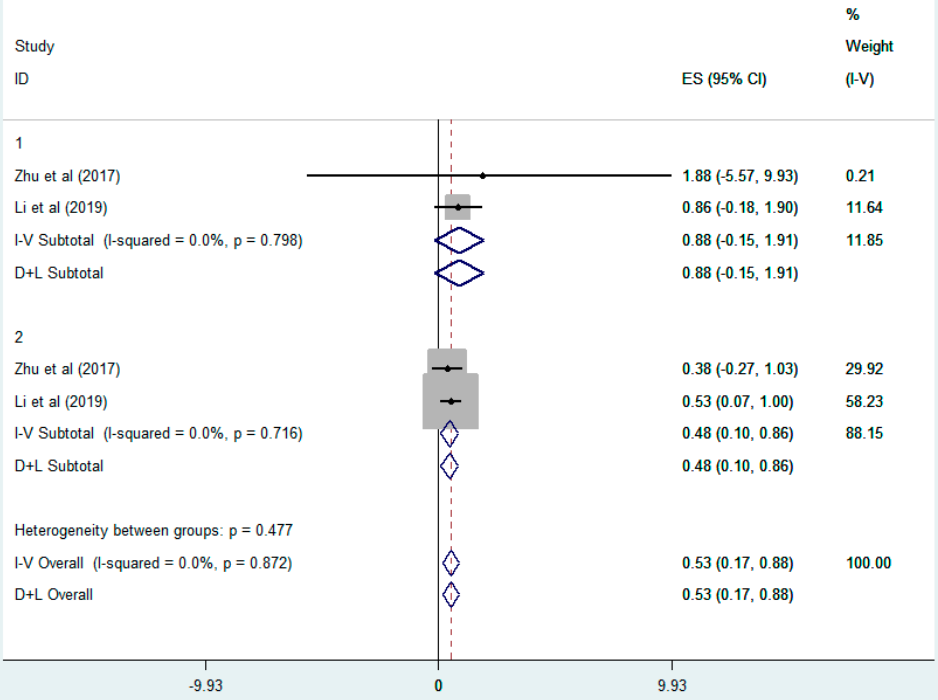
Study
ID
ES (95% CI)
%
Weight
(I-V)
1
Zhu et al (2017) 1.88 (-5.57, 9.93) 0.21
Li et al (2019) 0.86 (-0.18, 1.90) 11.64
I-V Subtotal (I-squared = 0.0%, p = 0.798) 0.88 (-0.15, 1.91) 11.85
D+L Subtotal 0.88 (-0.15, 1.91)
2
Zhu et al (2017) 0.38 (-0.27, 1.03) 29.92
Li et al (2019) 0.53 (0.07, 1.00) 58.23
I-V Subtotal (I-squared = 0.0%, p = 0.716) 0.48 (0.10, 0.86) 88.15
D+L Subtotal 0.48 (0.10, 0.86)
Heterogeneity between groups: p = 0.477
I-V Overall (I-squared = 0.0%, p = 0.872) 0.53 (0.17, 0.88) 100.00
D+L Overall 0.53 (0.17, 0.88)
-9.93
0
9.93

F

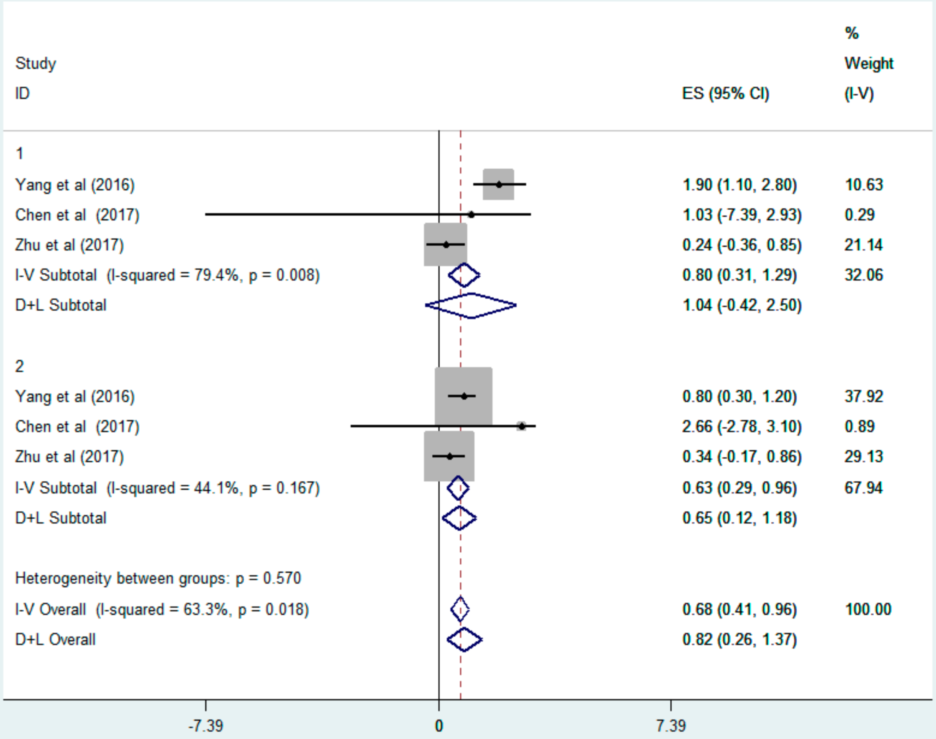
Study
ID
ES (95% CI)
%
Weight
(I-V)
1
Yang et al (2016) 1.90 (1.10, 2.80) 10.63
Chen et al (2017) 1.03 (-7.39, 2.93) 0.29
Zhu et al (2017) 0.24 (-0.36, 0.85) 21.14
I-V Subtotal (I-squared = 79.4%, p = 0.008) 0.80 (0.31, 1.29) 32.06
D+L Subtotal 1.04 (-0.42, 2.50)
2
Yang et al (2016) 0.80 (0.30, 1.20) 37.92
Chen et al (2017) 2.66 (-2.78, 3.10) 0.89
Zhu et al (2017) 0.34 (-0.17, 0.86) 29.13
I-V Subtotal (I-squared = 44.1%, p = 0.167) 0.63 (0.29, 0.96) 67.94
D+L Subtotal 0.65 (0.12, 1.18)
Heterogeneity between groups: p = 0.570
I-V Overall (I-squared = 63.3%, p = 0.018) 0.68 (0.41, 0.96) 100.00
D+L Overall 0.82 (0.26, 1.37)
-7.39
0
7.39

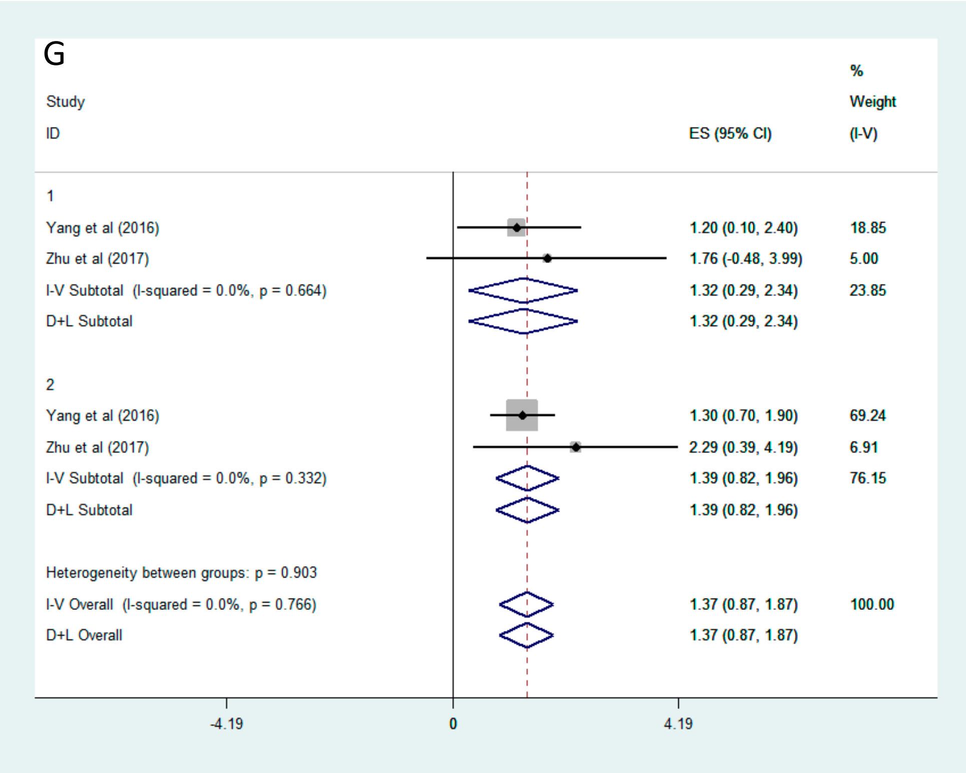
G
Study
ID
ES (95% CI)
%
Weight
(I-V)
1
Yang et al (2016) 1.20 (0.10, 2.40) 18.85
Zhu et al (2017) 1.76 (-0.48, 3.99) 5.00
I-V Subtotal (I-squared = 0.0%, p = 0.664) 1.32 (0.29, 2.34) 23.85
D+L Subtotal 1.32 (0.29, 2.34)
2
Yang et al (2016) 1.30 (0.70, 1.90) 69.24
Zhu et al (2017) 2.29 (0.39, 4.19) 6.91
I-V Subtotal (I-squared = 0.0%, p = 0.332) 1.39 (0.82, 1.96) 76.15
D+L Subtotal 1.39 (0.82, 1.96)
Heterogeneity between groups: p = 0.903
I-V Overall (I-squared = 0.0%, p = 0.766) 1.37 (0.87, 1.87) 100.00
D+L Overall 1.37 (0.87, 1.87)
-4.19
0
4.19

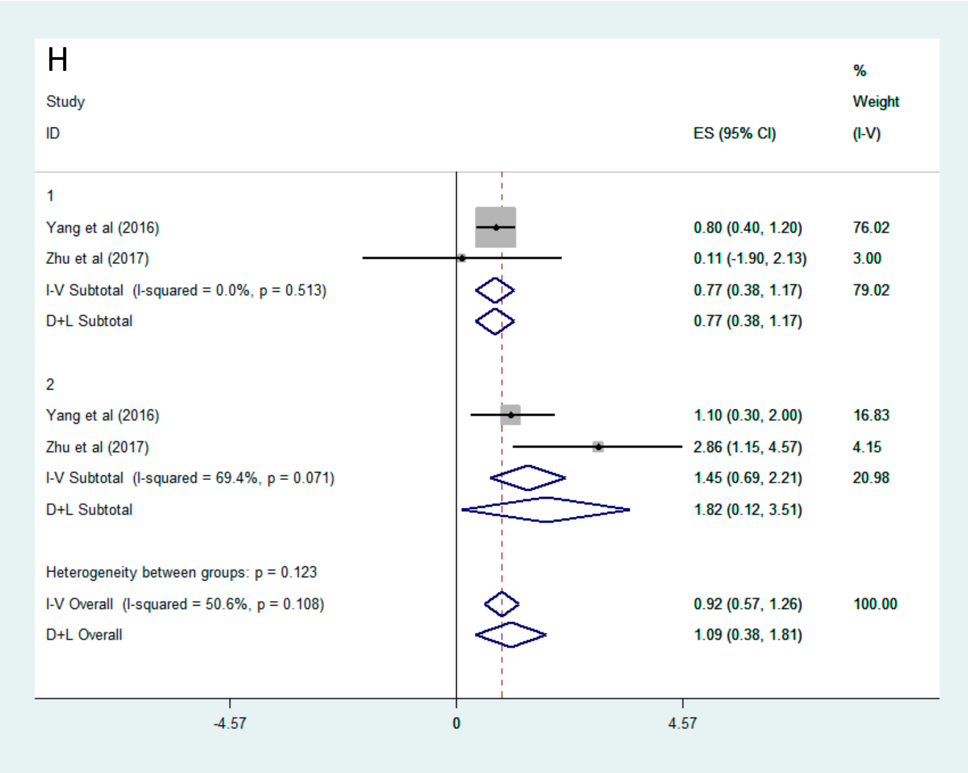
H
Study
ID
ES (95% CI)
%
Weight
(I-V)
1
Yang et al (2016) 0.80 (0.40, 1.20) 76.02
Zhu et al (2017) 0.11 (-1.90, 2.13) 3.00
I-V Subtotal (I-squared = 0.0%, p = 0.513) 0.77 (0.38, 1.17) 79.02
D+L Subtotal 0.77 (0.38, 1.17)
2
Yang et al (2016) 1.10 (0.30, 2.00) 16.83
Zhu et al (2017) 2.86 (1.15, 4.57) 4.15
I-V Subtotal (I-squared = 69.4%, p = 0.071) 1.45 (0.69, 2.21) 20.98
D+L Subtotal 1.82 (0.12, 3.51)
Heterogeneity between groups: p = 0.123
I-V Overall (I-squared = 50.6%, p = 0.108) 0.92 (0.57, 1.26) 100.00
D+L Overall 1.09 (0.38, 1.81)
-4.57
0
4.57

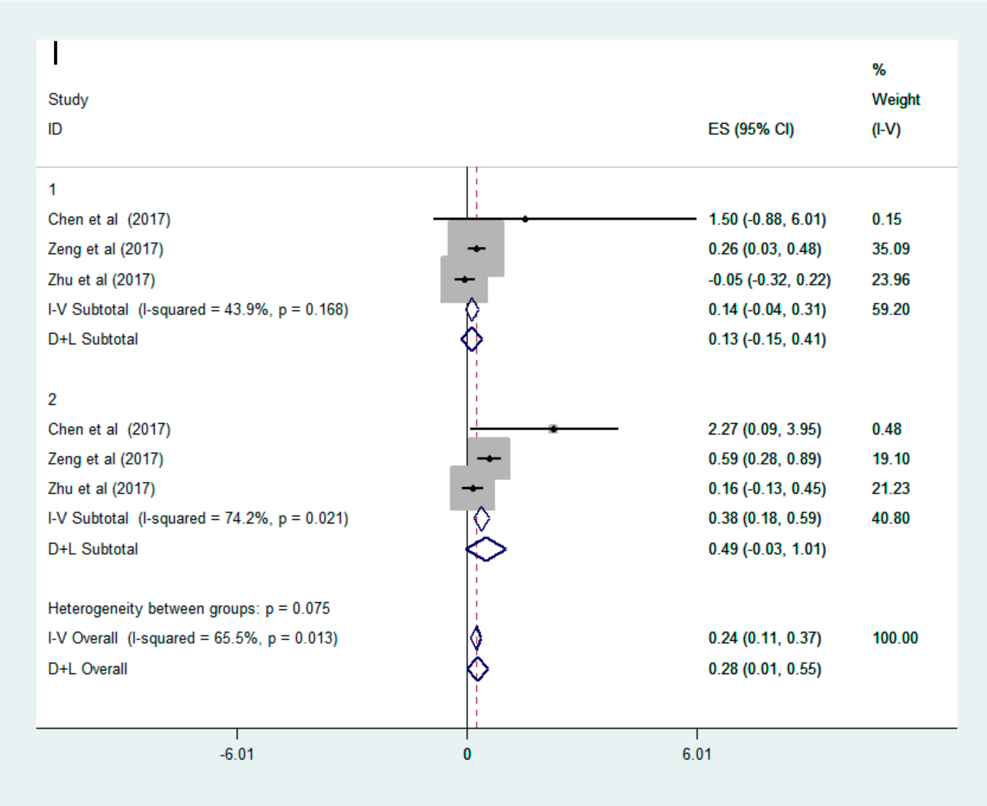

Figure S8 Forest plot and pooled effect estimates of the ambient air pollution on YLL for non-accidental diseases, cardiovascular disease (CVD) and respiratory disease (RD)stratified by age. A. $PM_{2.5}$ and non-accidental disease; B. PM_{10} and non-accidental disease; C. SO_2 and non-accidental disease; D. NO_2 and non-accidental disease; E. $PM_{2.5}$ and CVD; F.PM_{10} and CVD; G. SO_2 and CVD; H. NO_2 and CVD; I. PM_{10} and RD.Group 1: younger people; group 2: elder people.

A

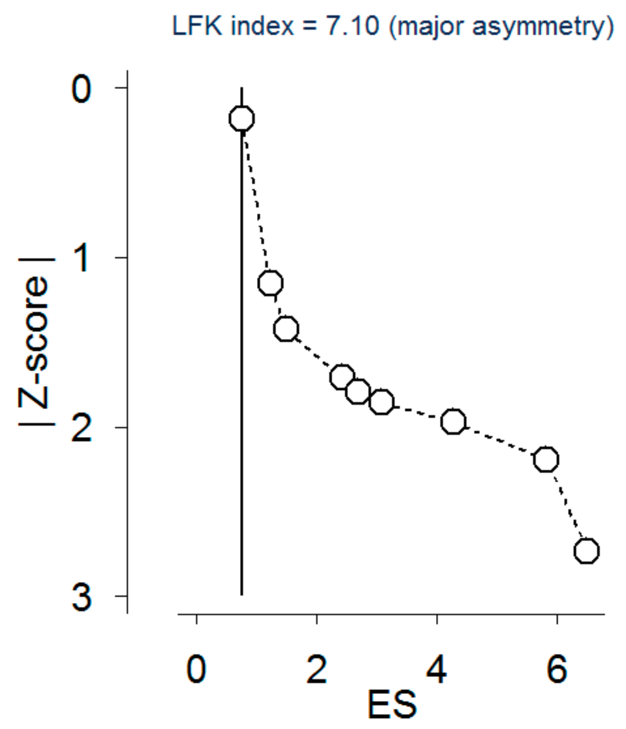
LFK index = 7.10 (major asymmetry)
| Z-score |
0
1
2
3
0
2
4
6
ES

B

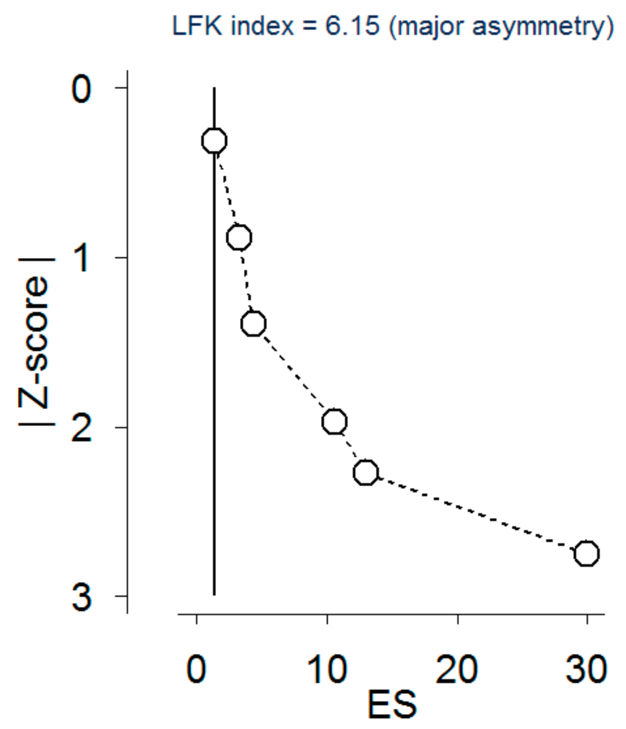
LFK index = 6.15 (major asymmetry)
| Z-score |
0
1
2
3
0
10
20
30
ES

C

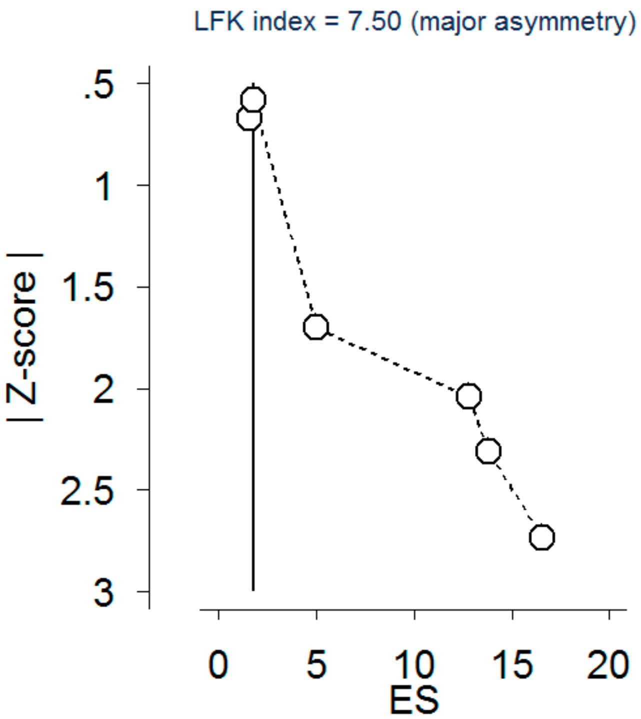

D

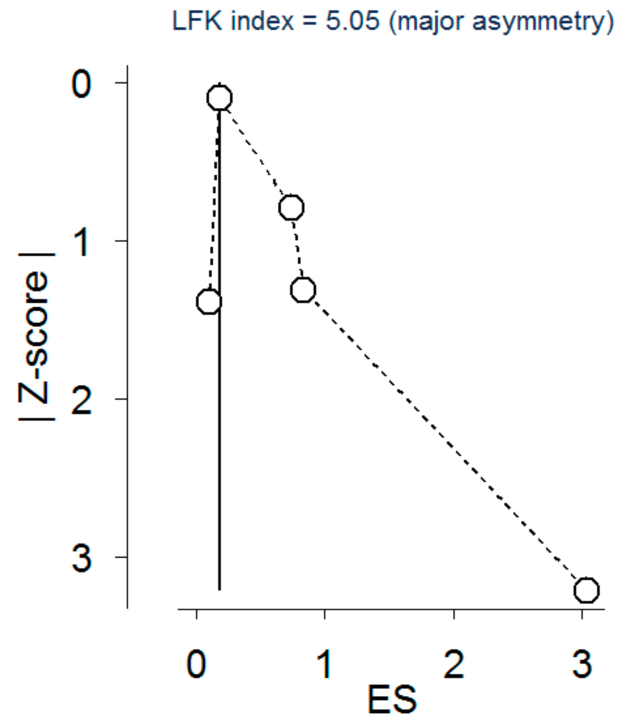

E

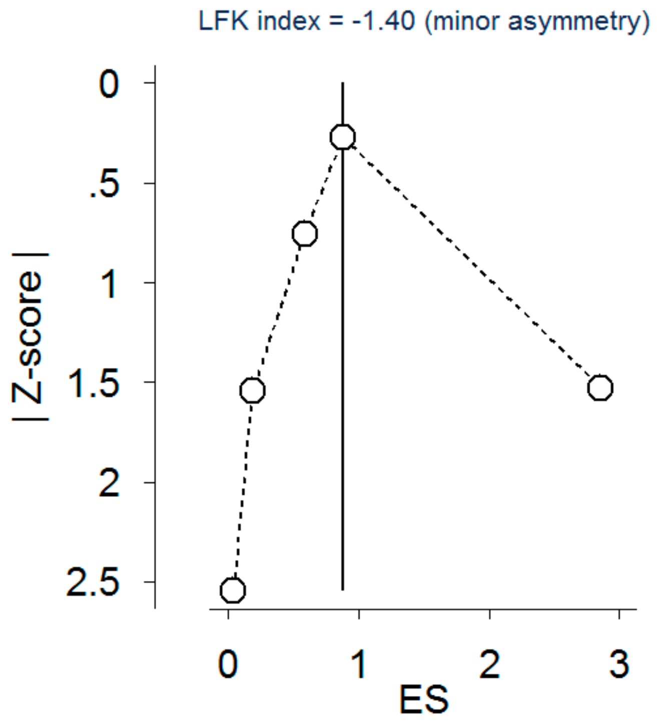

Figure S9 Doi plot and LFK index of the ambient air pollution on the

disease-specific YLL. A. PM_{10} and non-accidental diseases; B. SO_2 and non-accidental diseases; C. NO_2 and non-accidental diseases; D. PM_{10} and RD; E.PM_{10} and CVD.

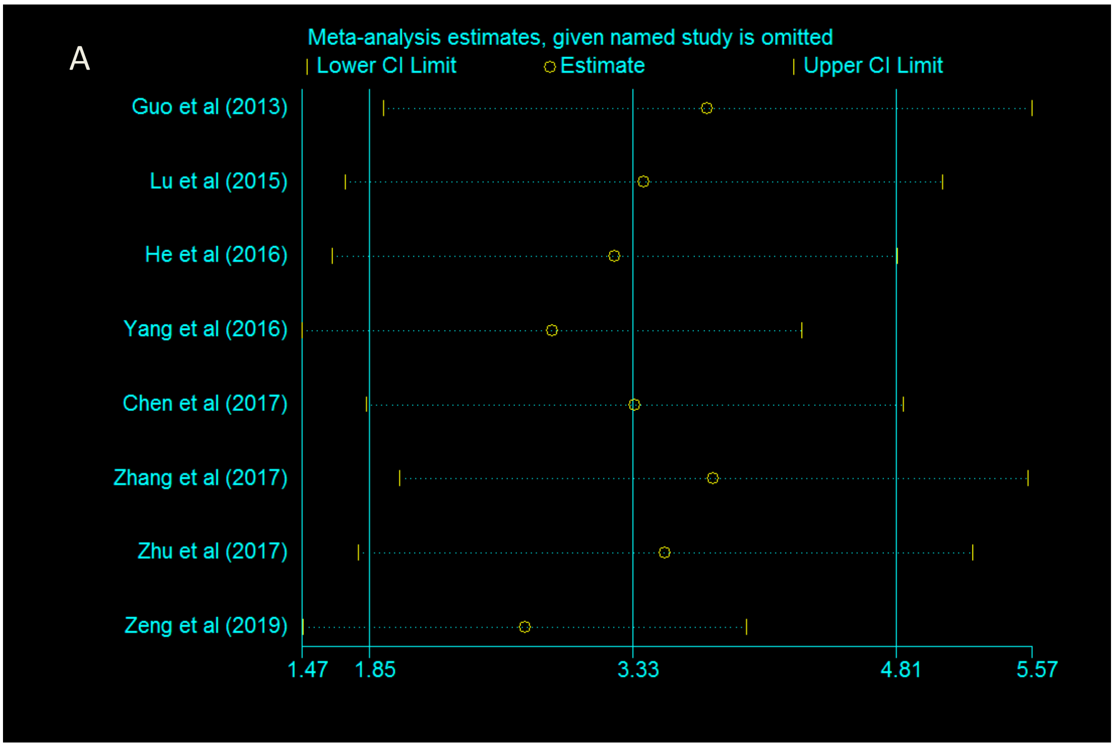

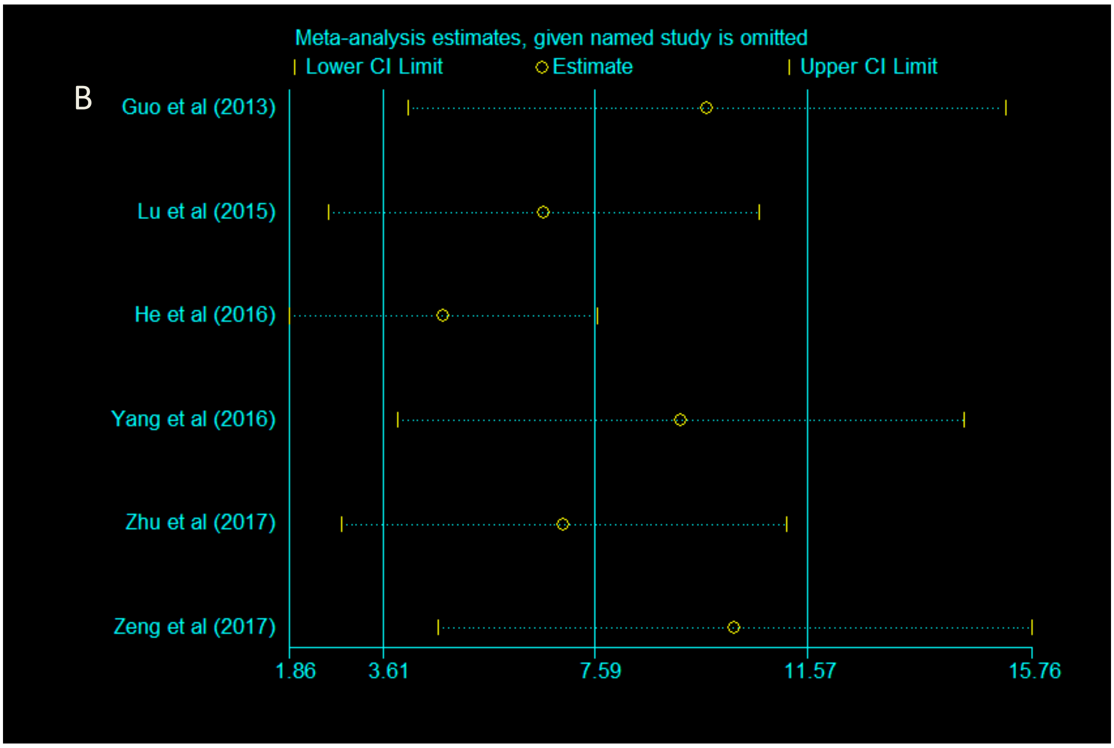

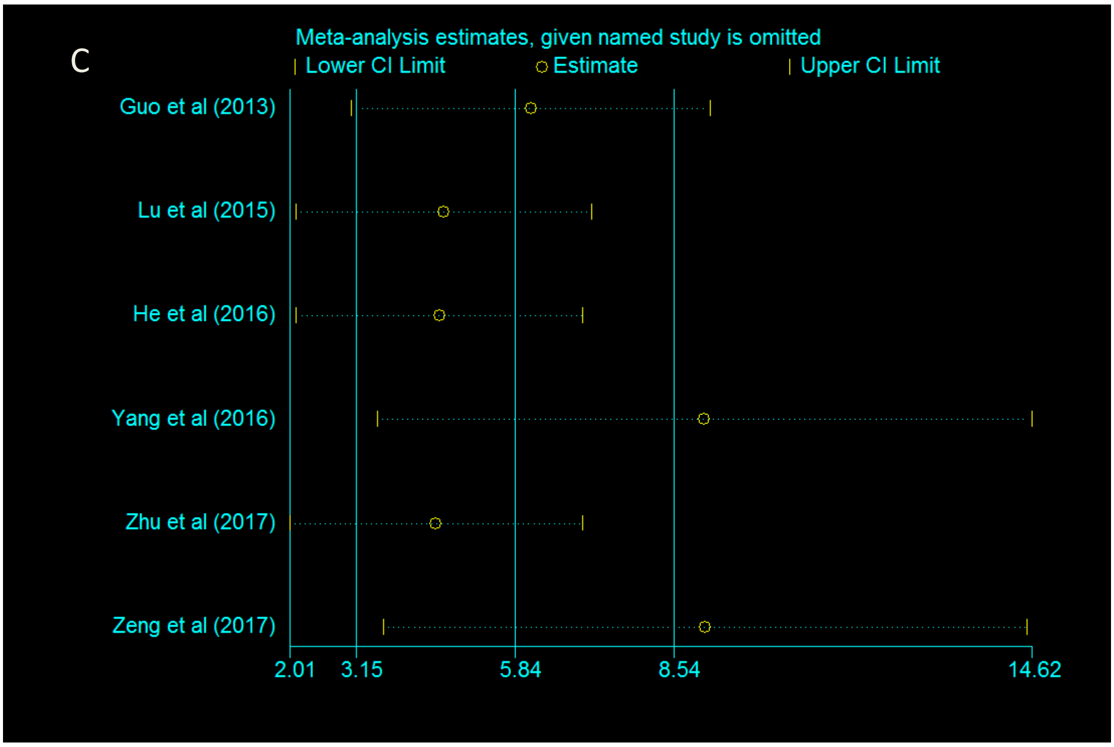
C
Meta-analysis estimates, given named study is omitted
Lower CI Limit
Estimate
Upper CI Limit
Guo et al (2013)
Lu et al (2015)
He et al (2016)
Yang et al (2016)
Zhu et al (2017)
Zeng et al (2017)
2.01
3.15
5.84
8.54
14.62

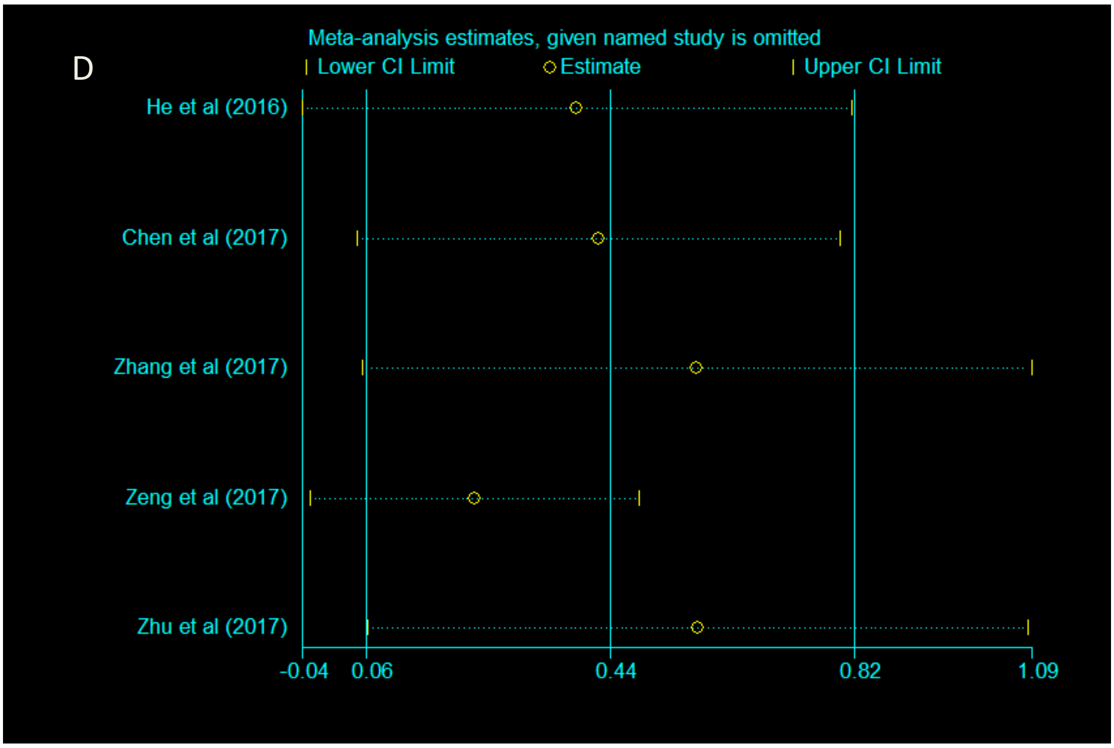
D
Meta-analysis estimates, given named study is omitted
Lower CI Limit
Estimate
Upper CI Limit
He et al (2016)
Chen et al (2017)
Zhang et al (2017)
Zeng et al (2017)
Zhu et al (2017)
-0.04
0.06
0.44
0.82
1.09

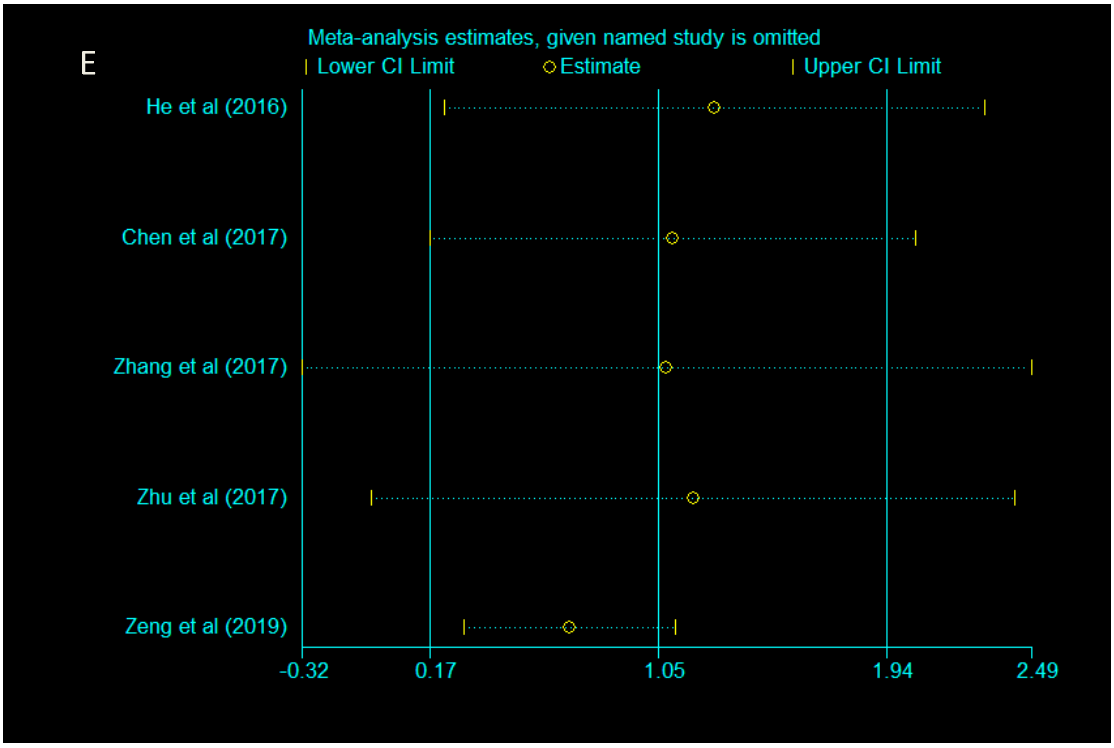

Figure S10 Sensitivity analyses of the ambient air pollution on the disease-specific YLL. A. PM_{10} and non-accidental diseases; B. SO_2 and non-accidental diseases; C. NO_2 and non-accidental diseases; D. PM_{10} and RD; E.PM_{10} and CVD.
